# Effectiveness and safety of follitropin delta in routine clinical practice in the Nordics and Switzerland (the NORSOS study): a prospective non-interventional study

**DOI:** 10.3389/fendo.2025.1613680

**Published:** 2025-09-05

**Authors:** Anja Pinborg, Negjyp Sopa, Erling Ekerhovd, Stavros I. Iliadis, June Kaspersen, Enrica Gravotta, Alexander Quaas

**Affiliations:** ^1^ Fertility Clinic, Department of Gynecology, Fertility and Obstetrics, Copenhagen University Hospital - Rigshospitalet, Copenhagen, Denmark; ^2^ Department of Clinical Medicine, University of Copenhagen, Copenhagen, Denmark; ^3^ Aleris Fertility, Aleris Hospital, Copenhagen, Denmark; ^4^ Fertility Department, Telemark Hospital, Porsgrunn, Norway; ^5^ Department of Women’s and Children’s Health, Uppsala University, Uppsala, Sweden; ^6^ Department of Obstetrics & Gynecology, Uppsala University Hospital, Uppsala, Sweden; ^7^ Ferring Lægemidler A/S, Kastrup, Denmark; ^8^ Global Research & Medical, Ferring Pharmaceutical A/S, Kastrup, Denmark; ^9^ Division of Reproductive Medicine and Gynecological Endocrinology, University Hospital Basel, Basel, Switzerland; ^10^ Shady Grove Fertility Center, San Diego, CA, United States

**Keywords:** follitropin delta, *in vitro* fertilization, assisted reproductive technology, anti-Müllerian hormone, pregnancy, ovarian stimulation, OHSS

## Abstract

**Background:**

Follitropin delta is the first approved human recombinant follicle-stimulating hormone treatment administered through an algorithmic individualized dosing regimen based on body weight and anti-Müllerian hormone (AMH) levels. This study assesses the effectiveness and safety profile of follitropin delta in women naïve to *in vitro* fertilization or intracytoplasmic sperm injection undergoing their first assisted reproductive technology cycle in a general clinical setting.

**Study design:**

This prospective observational study was conducted from August 2022 to March 2024 across 14 fertility clinics in Denmark, Norway, Sweden, and Switzerland. Consenting women with infertility (≥18 years old) receiving their first assisted reproductive technology cycle and first follitropin delta treatment were enrolled. Both fresh and frozen embryo transfers were evaluated. Follow-up continued until confirmed pregnancy outcome, early pregnancy loss, or study withdrawal. Data on follitropin delta usage, ovarian stimulation, embryo development, and safety were collected through electronic case-report forms. Patient-reported satisfaction with the follitropin delta pre-filled pen was assessed using patient questionnaires.

**Results:**

Of the 201 women enrolled, 199 completed the study. Of these, 147 (73.9%) were aged <35 years (median 32 years). The primary reason for infertility was male factor (88/199, 44.2%). Baseline characteristics included a mean body weight of 68.9 kg, and a mean AMH baseline concentration of 21.3 pmol/L, with 130/199 (65.3%) participants having AMH concentrations >15 pmol/L. Overall, 169/199 (84.9%) participants were prescribed follitropin delta according to the calculated algorithmic dose, with a mean starting dose of 10.2 µg and a mean duration of ovarian stimulation of 9.9 days. The gonadotropin-releasing hormone antagonist protocol was used in 171/193 (88.6%) women. Almost half of the analysis population (93/194, 47.9%) achieved the algorithm-targeted response of 8–14 oocytes retrieved, and >15 oocytes were obtained in 55/194 (28.4%) women. Ongoing pregnancy rate assessed by ultrasonography 10–11 weeks after embryo transfer was 82/155 (52.9%). Ovarian hyperstimulation syndrome (all mild cases) was reported in 8/199 (4.0%) women. Almost all women (190/193, 98.4%) expressed satisfaction with the injection pen.

**Conclusions:**

The NORSOS study (NCT05499052) provides insights into the use of follitropin delta in routine clinical practice and complements previous evidence regarding its effectiveness and safety profile.

## 1 Introduction

Ovarian stimulation is used to induce the production of an adequate number of retrievable oocytes in assisted reproductive technologies, such as *in vitro* fertilization (IVF) or intracytoplasmic sperm injection (ICSI). The objective is to achieve an optimal ovarian response, leading to higher rates of successful fertilization and pregnancy with minimal adverse outcomes (1, 2). Individual variability in ovarian response is influenced by the administered gonadotropin dose, the selected stimulation protocol, and the patient-specific characteristics (2). Healthcare professionals recognize that individualized ovarian stimulation dosing is essential for improving treatment outcomes and minimizing the risk of complications, such as ovarian hyperstimulation syndrome (OHSS), a rare but critical complication associated with gonadotropin use (3, 4). Physicians rely on various parameters, such as anti-Müllerian hormone (AMH) serum concentration or antral follicle count (AFC), along with their clinical experience, to predict ovarian response and make informed treatment decisions for each patient (5).

Follitropin delta (REKOVELLE^®^, Ferring Pharmaceuticals, Switzerland) is the first recombinant follicle-stimulating hormone (FSH) derived from a human cell line designed for individualized dosing using an approved algorithm based on baseline AMH concentrations and body weight (6). This individualized dosing regimen was established in a phase 2 study conducted in 265 women receiving IVF/ICSI, using pharmacokinetic and pharmacodynamic modeling and simulation (2, 6). Unlike other commercially available FSH preparations, follitropin delta is produced using a human cell line, resulting in a glycosylation profile that more closely resembles that of endogenous human FSH compared with recombinant follitropin alpha and beta (7).

Outcomes from randomized clinical trials (RCTs) in fresh cycles have shown that individualized follitropin delta dosing resulted in efficacy and safety profiles comparable to follitropin alpha and beta. The phase 3 ESTHER-1 trial showed that individualized follitropin delta dosing is non-inferior to conventionally dosed follitropin alpha for ongoing pregnancy and ongoing implantation rates, with significantly fewer extreme ovarian responses and reduced need for OHSS preventive measures (4). Patients who did not achieve an ongoing pregnancy were eligible to participate in the ESTHER-2 phase 3 trial, which confirmed the low immunogenicity potential of follitropin delta with repeated ovarian stimulation cycles (8). The phase 3 STORK trial established that individualized follitropin delta dosing in Japanese women is non-inferior to standard follitropin beta dosing in the number of oocytes retrieved, showing a favorable benefit–risk profile with a significant reduction in OHSS incidence without compromising live birth rates (9). The phase 3 GRAPE trial in Asian women undergoing their first IVF/ICSI cycle demonstrated that individualized follitropin delta dosing resulted in a non-inferior ongoing pregnancy rate, a significantly higher live birth rate, and significantly fewer early OHSS cases compared with follitropin alpha dosing (10). Analysis of two independent datasets comparing ovarian response in Asian patients undergoing IVF/ICSI with a gonadotropin-releasing hormone (GnRH) antagonist protocol established that a daily dose of 10 μg follitropin delta provides a similar ovarian response to a dose of 150 IU/day follitropin alpha (11). Additionally, the BEYOND study showed that using an individualized fixed-dose of follitropin delta in a GnRH agonist protocol is as effective as a GnRH antagonist protocol in European and Israeli women with AMH concentrations ≤35 pmol/L, resulting in a significantly higher number of oocytes retrieved and no increased risk of OHSS (12). Taken together, these findings confirm that individualized follitropin delta dosing is as effective as conventional FSH preparations, with the added benefit of a lower incidence of OHSS.

While RCTs are the gold standard for collecting insights into drug efficacy and safety, they are conducted under strict protocols and eligibility criteria that may not fully represent everyday clinical practice (13). Notably, some limitations of follitropin delta studies – such as variability in ovarian response across AMH and ethnic subgroups, lack of cumulative live birth data in early trials, and fixed maximum dosing restricting clinician-led adjustments (14, 15) – highlight the need for broader evidence. Although individualized follitropin delta dosing achieves efficacy comparable with other FSH preparations, it has not demonstrated superiority in pregnancy or live birth outcomes. Furthermore, despite its algorithm-driven dosing optimization, follitropin delta remains associated with typical gonadotropin-related adverse effects, including headache and pelvic discomfort (14, 15). Collecting evidence under empirical conditions is therefore crucial to capture the heterogeneity of routine clinical practice and diverse patient characteristics. Previous observational studies have demonstrated the effectiveness and safety profile of individualized follitropin delta dosing in different patient populations (16, 17). The NORdics and Switzerland Prospective Multicentre Non-Interventional ObServational Study to Assess the Pattern of Use of REKOVELLE^®^ in Women Undergoing IVF or ICSI Procedures in Routine Clinical Practice (NORSOS) aims to complement existing observational evidence. It reports on the treatment patterns, effectiveness, and safety of follitropin delta in women naïve to IVF/ICSI, undergoing their first ovarian stimulation treatment cycle with follitropin delta in countries where post-market authorization assessments have not been previously conducted. This study provides a comprehensive overview of follitropin delta usage, including the application of the individualized dosing algorithm, and offers new insights into self-reported patient satisfaction with follitropin delta in Denmark, Norway, Sweden, and Switzerland.

## 2 Materials and methods

### 2.1 Study design

NORSOS was a prospective, multicenter, post-authorization, non-interventional observational cohort study conducted between August 2022 and March 2024. The study was carried out at 14 IVF clinics across Denmark, Norway, Sweden, and Switzerland, known for treating and following-up patients undergoing controlled ovarian stimulation and routinely prescribing follitropin delta. Women were enrolled in the study after the decision to treat with follitropin delta had been made. No aspect of this study interfered with or influenced routine clinical procedures and/or medication received. The study was performed in compliance with the Declaration of Helsinki, current Guidelines for Good Pharmacoepidemiology Practice, and other national laws applicable in all participating countries, including local institutional review board ethics approval. All participants provided written informed consent as part of the enrollment process. The study was registered at ClinicalTrials.gov, with identifier NCT05499052 (**Supplementary Material 1**).

### 2.2 Study population

Women aged ≥18 years, who were IVF/ICSI treatment-naïve and planned to receive follitropin delta for their first cycle of IVF/ICSI using fresh or frozen sperm from a male partner or donor, were included in the study. Women were excluded if they were participating in ongoing interventional clinical trials requiring any treatment or follow-up, had any contraindications for follitropin delta treatment, planned to become oocyte donors, or were undergoing ovarian stimulation for fertility preservation.

### 2.3 Study drug

Participants administered follitropin delta using either the dosing algorithm based on body weight and AMH or a starting dose based on clinical judgment. Baseline AMH serum concentrations were assessed using the Elecsys^®^ AMH Plus immunoassay (Roche Diagnostics), the Access AMH Advanced (Beckman Coulter, Inc.), or the Lumipulse^®^ G AMH (Fujirebio) assays. AMH concentration was reported in pmol/L. If the AMH concentration was initially measured in ng/mL, this was converted to pmol/L using a conversion factor of 7.413. For measurements in other units, the AMH concentration was assigned a missing value. According to the algorithm, for women with AMH <15 pmol/L, the approved starting daily dose is 12 µg, regardless of body weight. For women with AMH ≥15 pmol/L, the daily dose decreases from 0.19 to 0.10 µg/kg depending on increasing AMH concentration. Follitropin delta was administered subcutaneously using a pre-filled injection pen (**Supplementary Material 2**) (6).

### 2.4 Data collection

During the observation period, the investigators collected data for one stimulation cycle with follitropin delta. Data regarding transfer cycles using frozen embryos were collected only for the first transfer cycle, provided it was performed within 3 months following the cycle stimulated with follitropin delta within the observation period. Women who underwent embryo transfer were followed until confirmation of ongoing pregnancy (approximately 10–11 weeks after first embryo transfer), no conception, early pregnancy loss, or study withdrawal. Owing to the observational nature of the study, all assessments were performed based on routine clinical practice. Data were collected via electronic case-report forms by the investigators during routine clinical care visits at enrollment, for the first ovarian stimulation cycle with follitropin delta, for follow-up, and in case of study withdrawal or treatment discontinuation. No visits were mandated or prescheduled as part of the study. Follow-up information was collected by the physicians at one single visit scheduled as a part of routine care or was collected via a telephone interview if a visit did not occur.

Baseline data collected at enrollment included sociodemographic data: age, body weight, height, most recent AMH concentration (within the last 12 months), AFC, laboratory assessments (e.g., FSH concentration), reproductive history, recent pelvic ultrasound results, and any other relevant medical history. Data collected for the first ovarian stimulation cycle with follitropin delta were: individualized dosing regimen, algorithm usage, ovarian stimulation protocol, cycle cancellation (and reason for cancellation), and adverse drug reactions. Follow-up data included ovarian response (number of oocytes retrieved and fertilized), freeze-all strategy, embryo transfer procedures, pregnancy outcomes, pregnancy loss, cycle and transfer cancellation (and reason for cancellation), adverse drug reactions, OHSS occurrence, preventive measures for OHSS, luteal-phase support (specifically whether a treatment was administered and the type of drug used), and recent ultrasound results (since last visit).All data were collected at the participating site in accordance with local clinical protocols or, when collected elsewhere, were obtained by the investigating clinician directly from the patient. Embryo quality was evaluated by the local clinical team, and transfers were generally performed 2–5 days after oocyte retrieval across all sites.

Patient satisfaction was assessed through questionnaires completed by the participants at the clinic after cycle cancellation, study withdrawal, or before oocyte retrieval. The questionnaire examined the ease of understanding the instructions and dosage preparation, the convenience of using the REKOVELLE^®^ pre-filled pen, and overall satisfaction. Responses were collected using a Likert-scale format, where participants rated their convenience and satisfaction on a scale ranging from “Very Difficult” to “Very Easy” and “Extremely Unsatisfied” to “Extremely Satisfied,” respectively (**Supplementary Table 1**).

### 2.5 Study outcomes

The primary objective was to observe and document the treatment patterns of follitropin delta in routine clinical practice, which included use of the algorithm-based individualized dosing regimen (starting daily dose and total dose of follitropin delta administered, duration of treatment), deviations from approved dosing algorithm, luteinizing hormone surge-suppression protocol, and the use of luteal-phase support drugs. Secondary objectives focused on ovarian stimulation and embryo development outcomes, such as the number of oocytes retrieved, the number of embryos frozen, the number of embryos transferred, the quality of fresh embryos transferred (defined as excellent, good, fair, or other), and the number of women with cycle cancellation before or after oocyte pickup (including reasons for cancellation). Pregnancy outcomes, such as human chorionic gonadotropin test, clinical pregnancy (at least one gestational sac 5–6 weeks post-transfer), ongoing pregnancy (≥1 intrauterine viable fetus 10–11 weeks post-transfer), and pregnancy loss in women with embryo transfer (defined as biochemical pregnancy, spontaneous abortion, or unknown) were also documented.

Both primary and secondary endpoints were documented for all women and for four subcategories based on age at baseline (<35 years, ≥35 and ≤37 years, >37 and ≤40 years, and >40 years).

The safety outcomes comprised the occurrence and severity of OHSS, the use of preventive interventions for risk of early OHSS, and any adverse drug reaction. These endpoints were reported using the Medical Dictionary for Regulatory Activities lowest-level terms.

Additionally, patient satisfaction was assessed, focusing on overall patient experience and convenience.

### 2.6 Statistical analysis

Sample size calculation indicated that 200 participants would be sufficient to provide meaningful estimates for the description of follitropin delta patterns of use, effectiveness, safety, and patient satisfaction at the first stimulation cycle. As the statistical analyses were purely descriptive, the sample size calculation was based on the precision of the confidence intervals (CIs) for observed frequencies. Each site planned to enroll up to 15 patients in a competitive manner during the predefined inclusion period, until the total planned 200 subjects were enrolled. The study enrollment was monitored and locked once 199 women initiated follitropin delta treatment.

All the analyses were conducted on available data from enrolled women who met eligibility criteria and received at least one dose of follitropin delta (study population).

All primary and secondary endpoints were summarized using descriptive statistics and stratified by age category. Categorical variables were summarized by frequency counts (n) and percentages (%) of total participants in each category, unless otherwise specified. Continuous variables included the number of non-missing observations (n) and the mean and standard deviation. Two-sided 95% CIs were considered as the default (α = 0.05%).

As the statistical analyses were purely descriptive, the sample size was not based on any inferential statistical considerations. A planned study sample size of 200 participants was considered sufficient to ensure meaningful data were obtained for the description of follitropin use patterns, effectiveness, safety, and patient satisfaction at the first follitropin delta cycle.

## 3 Results

### 3.1 Patient demographics and baseline characteristics

**Figure 1:**
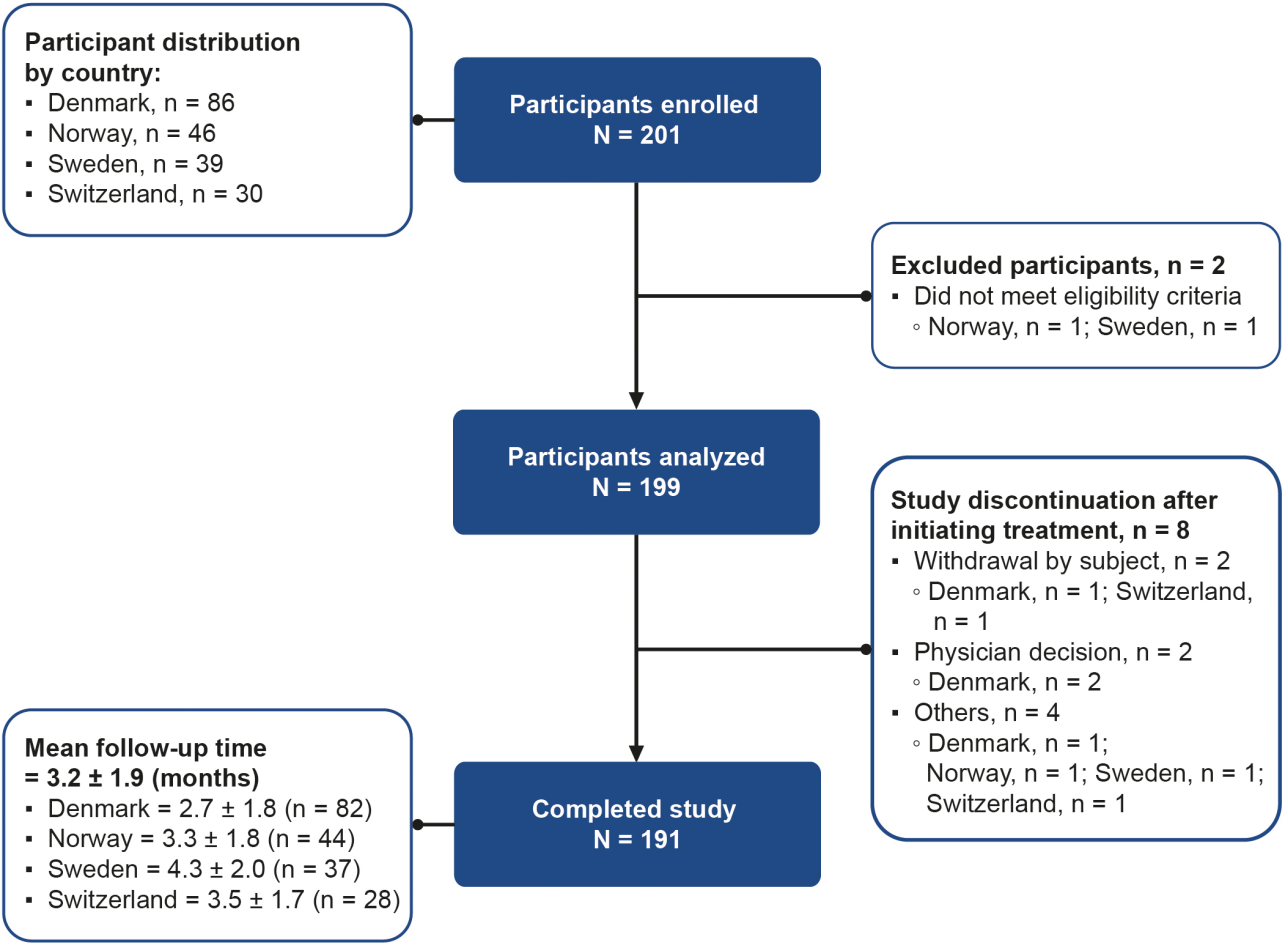
Participant flow diagram from enrollment to study completion. Participants who completed the study are those who became pregnant or had undergone one cycle of ovarian stimulation with follitropin delta and finished follow-up. Participants who discontinued are those with any reason for study termination after initiating treatment, except for pregnancy or undergoing one cycle of ovarian stimulation with follitropin delta. “Others” includes spontaneous pregnancy before treatment (n = 3), and elective abortion due to multiple fetal malformations (n = 1). N, total number of participants; n, number of participants per country.

A total of 201 women were enrolled across Denmark (n = 86, 42.8%), Norway (n = 46, 22.9%), Sweden (n = 39, 19.4%), and Switzerland (n = 30, 14.9%). Of the 201 enrolled participants, two women were excluded because they did not meet the inclusion criteria; therefore, 199 women received ≥1 dose of follitropin delta (study population). Eight participants were discontinued from the study after initiating treatment. Among these, the reasons for discontinuation included participant’s decision (2/199, 1.0%), physician’s decision (2/199, 1.0%), natural conception before treatment (3/199, 1.5%), or elective abortion due to multiple fetal malformations (1/199, 0.5%). A total of 191/199 (96.0%) women completed the study after a mean follow-up period of 3.2 ± 1.9 months (**Figure 1**).

At baseline, the mean age of the study population (n = 199) was 32.0 ± 4.0 years. Of these, 147 (73.9%) women were <35 years of age, 31 (15.6%) women were ≥35 and ≤37 years, 18 (9.0%) women were >37 and ≤40 years, and 3 (1.5%) women were >40 years. The age distribution varied across countries, as a higher percentage of participants in Sweden (19/38, 50.0%) and Switzerland (10/30, 33.3%) were older than 35 years compared with those in Denmark (17/86, 19.8%) and Norway (6/45, 13.3%) (**Table 1**).

**Table 1:** Patient demographics and baseline characteristics by country.

Patient characteristics	Overall	By country			
		Denmark	Norway	Sweden	Switzerland
Number of participants, N (%)	199 (100.0)	86 (43.2)	45 (22.6)	38 (19.1)	30 (15.1)
Age at baseline (years)	32.0 ± 4.0	31.3 ± 4.0	30.8 ± 3.2	33.9 ± 3.6	33.4 ± 4.5
<35	147 (73.9)	69 (80.2)	39 (86.7)	19 (50.0)	20 (66.7)
≥35 and ≤37	31 (15.6)	11 (12.8)	5 (11.1)	12 (31.6)	3 (10.0)
>37 and ≤40	18 (9.0)	6 (7.0)	1 (2.2)	7 (18.4)	4 (13.3)
>40	3 (1.5)	0	0	0	3 (10.0)
Height (m)	1.67 ± 0.06	1.69 ± 0.06	1.66 ± 0.06	1.67 ± 0.06	1.65 ± 0.06
Body weight (kg)	68.9 ± 11.6	69.2 ± 10.9	68.4 ± 12.0	69.8 ± 10.6	67.4 ± 14.2
Body mass index (kg/m^2^)	24.6 ± 4.2	24.3 ± 3.9	24.7 ± 4.1	25.1 ± 4.1	24.7 ± 5.2
AMH concentration (pmol/L)	21.3 ± 12.5	21.4 ± 11.4	23.8 ± 12.4	16.5 ± 9.5	23.4 ± 17.0
AMH (in categories) (pmol/L)^a^					
<7	15 (7.5)	6 (7.0)	0	5 (13.2)	4 (13.3)
≥7 and <15	54 (27.1)	21 (24.4)	10 (22.2)	14 (36.8)	9 (30.0)
≥15 and ≤35	104 (52.3)	47 (54.7)	28 (62.2)	19 (50.0)	10 (33.3)
>35	26 (13.1)	12 (14.0)	7 (15.6)	0	7 (23.3)
Antral follicle count					
Women measured	185 (93.0)	85 (98.8)	33 (73.3)	37 (97.4)	30 (100.0)
Mean count	19.2 (9.7)	18.3 (8.9)	20.1 (9.9)	18.5 (7.5)	21.5 (13.7)
FSH concentration					
Women measured	110 (55.3)	26 (30.2)	45 (100.0)	10 (26.3)	29 (96.7)
Mean concentration (IU/L)	6.7 ± 2.1	6.3 ± 1.6	6.4 ± 1.9	6.9 ± 1.7	7.4 ± 2.7
Infertility history^b^					
Duration of infertility (years)	2.4 ± 1.6	2.2 ± 1.7	2.7 ± 1.3	2.7 ± 2.1	2.6 ± 1.2
Primary infertility	136 (68.7)	61 (70.9)	30 (66.7)	26 (70.3)	19 (63.3)
Reason(s) for infertility					
Male factor	88 (44.2)	43 (50.0)	19 (42.2)	10 (26.3)	16 (53.3)
Unexplained infertility	71 (35.7)	29 (33.7)	18 (40.0)	17 (44.7)	7 (23.3)
Tubal infertility	11 (5.5)	5 (5.8)	2 (4.4)	3 (7.9)	1 (3.3)
Anovulatory infertility (WHO Groups I and II)	10 (5.0)	2 (2.3)	5 (11.1)	1 (2.6)	2 (6.7)
Endometriosis	6 (3.0)	0	1 (2.2)	1 (2.6)	4 (13.3)
Other	13 (6.5)	7 (8.1)	0	6 (15.8)	0

Data are mean ± SD or n (%), unless stated otherwise. Percentages are calculated based on the total number of women in the study (n = 199) and the number of participants per country (N).

a Conversions: 7 pmol/L = 0.98 ng/mL; 15 pmol/L = 2.1 ng/mL; 35 pmol/L = 4.9 ng/mL.

b There is a missing data point in the infertility history data for the Sweden cohort (n = 37).

AMH, anti-Müllerian hormone; FSH, follicle-stimulating hormone; N, number of participants; SD, standard deviation; WHO, World Health Organization.

The mean body weight of the study population (n = 199) was 68.9 ± 11.6 kg, the mean body mass index (BMI) was 24.6 ± 4.2 kg/m^2^, and the mean baseline AMH serum concentration was 21.3 ± 12.5 pmol/L. Approximately half of the participants (104/199, 52.3%) had an AMH concentration between ≥15 and ≤35 pmol/L, followed by 54/199 (27.1%) participants who had AMH concentrations between ≥7 and <15 pmol/L. A total of 26/199 (13.1%) participants had AMH concentrations >35 pmol/L, while 15/199 (7.5%) participants had low AMH concentrations (<7 pmol/L). Participants from Sweden had numerically lower AMH concentrations (16.5 ± 9.5 pmol/L) compared with other countries. Notably, none of the Swedish participants had AMH concentrations above 35 pmol/L (**Table 1**).

The mean AFC was 19.2 ± 9.7 among 185/199 (93.0%) of the participants for whom AFC was recorded. The mean FSH value was 6.7 ± 2.1 IU/L for participants with recorded FSH concentrations (110/199, 55.3%). Both the AFC and FSH levels were relatively consistent across countries (**Table 1**).

The mean duration of infertility among all participants was 2.4 ± 1.6 years, with primary infertility observed in 136/198 (68.7%) participants. Male factor was the most common reason for infertility in 88/199 (44.2%) women, followed by unexplained infertility in 71/199 (35.7%) women (**Table 1**).

### 3.2 Follitropin delta treatment patterns

Overall, the mean starting dose of follitropin delta was 10.2 ± 2.3 µg (**Table 2**). Follitropin delta starting dose was prescribed according to the calculated algorithmic dose for 169/199 (84.9%) women, with only minor differences observed between the average daily starting dose of follitropin delta prescribed by the physician and the dose calculated by the approved algorithm (−0.01 ± 1.0 µg). Among the 30 participants whose starting dose deviated from the dosing algorithm, a significant majority (22/30, 73.3%) were younger than 35 years old. The difference in the starting dose calculated by the physician was more than 0.33 µg (one click of the pen) for 17/199 (8.5%) participants and less than 0.33 µg for 13/199 (6.5%) participants.

**Table 2:** Follitropin delta treatment patterns per age category.

Treatment patterns	Overall	By age group (years)			
		<35	≥35 and ≤37	>37 and ≤40	>40
Number of participants, N	199	147	31	18	3
Daily starting dose prescribed (µg)	10.2 ± 2.3	9.99 ± 2.3	10.9 ± 2.6	10.5 ± 2.1	10.7 ± 2.3
Calculated algorithmic dose (µg)	10.2 ± 2.1	10.1 ± 2.2	10.5 ± 1.8	10.5 ± 2.1	12.0 ± 0.0
Used the dosing app (Yes)	57 (28.6)	42 (28.6)	6 (19.4)	6 (33.3)	3 (100.0)
Dose deviation between starting dose prescribed and calculated dose (µg)	−0.01 ± 1.0	−0.07 ± 0.8	0.44 ± 1.6	−0.07 ± 0.8	−1.33 ± 2.3
Starting-dose comparison between prescribed and calculated dose					
Lower dose (difference <0.33 µg)	13 (6.5)	10 (6.8)	0	2 (11.1)	1 (33.3)
Same dose (± 0.33 µg)	169 (84.9)	125 (85.0)	28 (90.3)	14 (77.8)	2 (66.7)
Higher dose (difference >0.33 µg)	17 (8.5)	12 (8.2)	3 (9.7)	2 (11.1)	0
Daily dose adjusted during ovarian stimulation^a^	12 (6.0)	7 (4.8)	1 (3.2)	2 (11.1)	2 (66.7)
If starting dose adjusted, type of adjustment					
Any	12 (6.0)	7 (4.7)	1 (3.2)	2 (11.1)	2 (66.7)
Increased	4 (2.0)	3 (2.0)	1 (3.2)	0 (0.0)	0 (0.0)
Decreased	8 (4.0)	4 (2.7)	0	2 (11.1)	2 (66.7)
Total dose administered, based on starting and adjusted dose(s) (µg)	101.2 ± 31.0	99.8 ± 28.4	110.2 ± 43.0	100.5 ± 27.4	80.0 ± 13.9
Duration of ovarian stimulation, including adjusted dose (days)^b^	9.9 ± 1.7	9.9 ± 1.7	10.3 ± 1.8	9.8 ± 1.5	8.3 ± 1.5

Data are mean ± SD or n (%), unless stated otherwise. Percentages calculated based on the total number of women in the study and the number of participants per age group (N).

a All adjustments were considered in this variable, including participants with multiple dose adjustments, but each participant is counted only once.

b Duration of treatment = (stimulation day end with follitropin delta – stimulation day start with follitropin delta) + 1.

N, number of participants; SD, standard deviation.

During ovarian stimulation, daily dose adjustments were reported in 12/199 (6.0%) participants (**Table 2**). The daily dose was increased in 4/12 (33.3%) women and decreased in 8/12 (66.7%) women. The mean total dose of follitropin delta administered was 101.2 ± 31.0 µg for all participants over a mean stimulation period of 9.9 ± 1.7 days. In the small cohort of women above 40 years of age (n = 3), the mean total dose of follitropin delta and the duration of ovarian stimulation were 80.0 ± 13.9 µg and 8.3 ± 1.5 days, respectively. A GnRH antagonist protocol was used in 171/193 (88.6%) women.

### 3.3 Ovarian response

**Figure 2:**
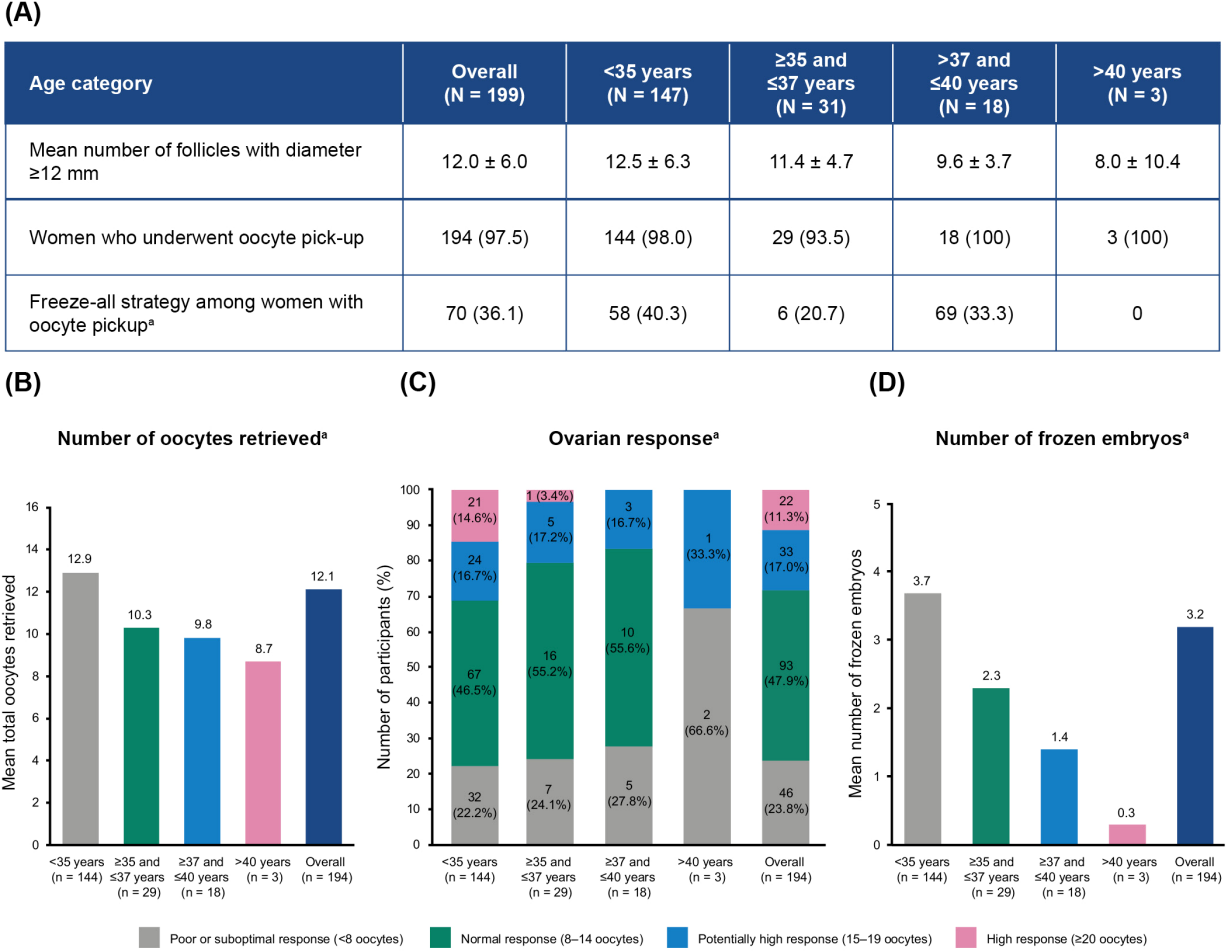
Oocyte retrieval and cryopreservation in participants without cancellation before oocyte pickup, by age category. **(A)** Freeze-all strategy. **(B)** Mean total number of oocytes retrieved. **(C)** Distribution of ovarian responses. **(D)** Mean number of frozen embryos obtained. Values represented as mean ± SD or n (%), unless stated otherwise. ^a^Percentages calculated based on the total number of women in the study who underwent oocyte pickup. N, total number of participants; n, number of participants per subgroup; SD, standard deviation.

On average, 12.5 ± 6.3 follicles reached a diameter of at least 12 mm (**Figure 2A**). A total of 194/199 (97.5%) women underwent oocyte pickup with a mean number of 12.1 ± 6.9 oocytes retrieved per woman (**Figure 2A**). Women aged <35 years had an average of 12.9 ± 7.4 oocytes retrieved, while women >40 years reported 8.7 ± 8.3 oocytes retrieved (**Figure 2B**). Moreover, women with AMH levels >35 pmol/L had an average of 15.1 ± 10.3 oocytes retrieved, compared with 7.0 ± 5.1 oocytes for women with AMH levels <7 pmol/L (**Supplementary Table 2**). The algorithm-targeted response of 8–14 retrieved oocytes after a fresh transfer was obtained in 93/194 (47.9%) of the women (**Figure 2C**). An oocyte yield of >15 oocytes was observed in 55/194 (28.4%) participants across all ages.

#### 3.3.1 Cycle cancellation and cryopreservation

Overall, cycle cancellation was reported in 5/199 (2.5%) women, all of whom were ≤37 years (**Supplementary Table 3**). The reasons for cancellation were poor ovarian response (n = 2), participant choice (n = 2), and participant not taking ovulation triggering at correct time (n = 1). A freeze-all strategy was implemented for 70 (36.1%) of the women who underwent oocyte pickup (n = 194). The mean overall number of frozen embryos was 3.2 ± 3.8 per woman (**Figure 2D**).

A total of 106/199 (53.3%) women had a fresh embryo transfer, most of which were single transfers, while 88/199 (44.2%) participants had their fresh transfer canceled after oocyte retrieval (**Supplementary Table 3**). The most common reason for fresh embryo transfer cancellation was “Other” (62/88, 70.5%), which included implementation of a freeze-all strategy as a preventive measure for OHSS, failure of the blastocyst to reach day 5, or pending results from embryo genetic testing. The following most common reasons were no embryo development (10/88, 11.4%), abnormal embryo development (6/88, 6.8%), and early OHSS (6/88, 6.8%). Four women (4/88, 4.5%) had their transfer canceled due to either a lack of fertilized oocytes or abnormal fertilization.

### 3.4 Embryo transfer procedures

Transfer procedures outcomes by age category are represented in **Table 3**. Among the 199 women included in the study, 155 (77.9%) underwent at least one embryo first transfer (fresh or frozen).

**Table 3:** Embryo transfer procedures per age group.

Transfer Procedures	Overall	By age group (years)			
		<35	≥35 and ≤37	>37 and ≤40	>40
Number of participants, N	199	147	31	18	3
Women without embryo transfers^a^	44 (22.1)	30 (20.4)	9 (29.0)	4 (22.2)	1 (33.3)
Women with one embryo transfer^a^	122 (61.3)	98 (66.7)	12 (38.7)	10 (55.6)	2 (66.7)
Women with two embryo transfers^a^	33 (16.6)	19 (12.9)	10 (32.3)	4 (22.2)	0
Women with first fresh transfer^a^	106 (53.3)	74 (50.3)	19 (61.3)	11 (61.1)	2 (66.7)
Single^b^	98 (92.5)	67 (90.5)	18 (94.7)	11 (100)	2 (100)
Excellent quality embryo^c^	71 (72.4)	52 (77.6)	13 (72.2)	6 (54.5)	0
Good quality embryo^c^	21 (21.4)	14 (20.9)	4 (22.2)	2 (18.2)	1 (50.0)
Fair quality embryo^c^	5 (5.1)	1 (1.5)	1 (5.6)	3 (27.3)	0
Other quality embryo^c^	1 (1.0)	0	0	0	1 (50.0)
Double^b^	8 (7.5)	7 (9.5)	1 (5.3)	0	0
Women with first frozen transfer^a^	49 (24.6)	43 (29.3)	3 (9.7)	3 (16.7)	0
Women with frozen transfer after a fresh transfer^a^	33 (16.6)	19 (12.9)	10 (32.3)	4 (22.2)	0

Values represented as n (%), unless stated otherwise.

a Percentages calculated based on the total number of women in the study (N).

b Percentages calculated from number of women with a fresh transfer. Reasons for double transfer included factors such as embryo quality or patient preference.

c Percentages calculated from number of women with a single fresh transfer.

N, number of participants.

Approximately half of all the participants (106/199, 53.3%) had a first fresh transfer. This was observed across the different age groups (**Table 3**). Of the 98/106 (92.5%) women who underwent a single fresh transfer, 71/98 (72.4%) had an excellent quality embryo and 21/98 (21.4%) had a good quality embryo. This finding was relatively consistent among most age groups, with slightly lower values observed among women aged >37 years (**Table 3**). A small proportion of women received a double embryo transfer (8/199, 7.5%), with nearly all (7/147, 9.5%) being under the age of 35.A first frozen transfer was reported for 49/199 (24.6%) participants, while 44/199 (22.1%) women did not undergo any transfer (**Table 3**).

Luteal-phase support drugs were administered to 125/194 (64.4%) women, with progesterone being the most common luteal-phase drug administered to nearly all (124/125, 99.2%) of them (**Supplementary Table 4**).

### 3.5 Pregnancy outcomes

Cumulatively, ongoing pregnancy occurred in 82/199 (41.2%) of the analysis population. Among all women who underwent a first transfer (fresh or frozen; n = 155), 70 (45.2%) achieved an ongoing pregnancy (**Table 4**). The proportion of this group achieving an ongoing pregnancy was higher among women under 35 years of age (59/117; 50.4%), and lower among women aged 37 years or above (4/16; 25.0%). A total of 10/155 (6.5%) women experienced a spontaneous abortion after a first transfer, while 9/155 (5.8%) experienced a biochemical pregnancy (**Table 4**).

**Table 4:** Pregnancy outcomes per age category.

Pregnancy Outcomes	Overall	By age group (years)			
		<35	≥35 and ≤37	>37 and ≤40	>40
Women with first transfer^a^, N (fresh or frozen)	155	117	22	14	2
Positive hCG	89 (57.4)	74 (63.2)	8 (36.4)	6 (42.9)	1 (50.0)
Clinical pregnancy	77 (49.7)	64 (54.7)	7 (31.8)	5 (35.7)	1 (50.0)
Ongoing pregnancy	70 (45.2)	59 (50.4)	7 (31.8)	3 (21.4)	1 (50.0)
Pregnancy loss					
Biochemical pregnancy	9 (5.8)	8 (6.8)	1 (4.5)	0	0
Spontaneous abortion	10 (6.5)	7 (6.0)	0	3 (21.4)	0
Unknown	1 (0.6)	0	1 (4.5)	0	0
Women with fresh transfer, N	106	74	19	11	2
Positive hCG	54 (50.9)	43 (58.1)	5 (26.3)	5 (45.5)	1 (50.0)
Clinical pregnancy	46 (43.4)	36 (48.6)	5 (26.3)	4 (36.4)	1 (50.0)
Ongoing pregnancy	43 (40.6)	35 (47.3)	5 (26.3)	2 (18.2)	1 (50.0)
Pregnancy loss					
Biochemical pregnancy	5 (4.7)	5 (6.8)	0	0	0
Spontaneous abortion	6 (5.7)	3 (4.1)	0	3 (27.3)	0
Unknown	1 (0.9)	0	1 (5.3)	0	0
Women with multiple transfers^b^, N	155	117	22	14	2
Positive hCG	103 (66.5)	82 (70.1)	12 (54.5)	8 (57.1)	1 (50.0)
Clinical pregnancy	91 (58.7)	72 (61.5)	11 (50.0)	7 (50.0)	1 (50.0)
Ongoing pregnancy	82 (52.9)	67 (57.3)	9 (40.9)	5 (35.7)	1 (50.0)
Pregnancy loss					
Biochemical pregnancy	9 (5.8)	8 (6.8)	1 (4.5)	0	0
Spontaneous abortion	12 (7.7)	7 (6.0)	2 (9.1)	3 (21.4)	0
Unknown	1 (0.6)	0	1 (4.5)	0	0

Values represented as n (%), unless stated otherwise. Percentages calculated based on the total number of women with transfer per respective age group (N).

a Includes any patient receiving either a fresh or frozen transfer during their first stimulation cycle.

b Includes any patients who had more than one transfer (fresh or frozen, including a frozen transfer after an initial fresh transfer). In this case, only the latest transfer was considered.

hCG, human chorionic gonadotropin; N, number of participants.

Among the women with a fresh transfer (n = 106), 43 (40.6%) had an ongoing pregnancy (**Table 4**). Of the women who underwent at least one transfer of any kind (n = 155), 82 (52.9%) had an ongoing pregnancy (**Table 4**).

### 3.6 Safety and OHSS occurrence

OHSS occurred in 8/199 (4.0%) women. There were no adverse drug reactions reported in this study that led to either temporary or permanent discontinuation, nor were any deaths reported.

All OHSS cases were mild in severity, and the majority (6/8, 75.0%) had an early onset (<9 days after triggering). Preventive measures for risk of early OHSS were taken for 58/199 (29.1%) women, with each participant reporting one or more preventive interventions, which were not mutually exclusive. The most common preventive measures were embryo transfer cancellation for 53/58 (91.4%) women and triggering of final follicular maturation with a GnRH agonist followed by cryopreservation of all oocytes or embryos for 44/58 (75.9%) women. OHSS and preventive measures are presented in **Table 5**.

**Table 5:** OHSS and preventive measures.

Safety outcomes	Overall
Number of participants, N	199
Women with OHSS^a^	8 (4.0)
OHSS by severity^b^	
Mild	8 (100)
OHSS by onset^b^	
Early	6 (75.0)
Late	1 (12.5)
All	7 (87.5)
Missing	1
Preventive intervention for risk of early OHSS^a^	58 (29.1)
Type of intervention taken^c^	
Embryo transfer cancellation	53 (91.4)
Triggering of final follicular maturation with GnRH agonist and fresh embryo transfer	4 (6.9)
Triggering of final follicular maturation with GnRH agonist and with cryopreservation of all oocytes/embryos	44 (75.9)
Administration of dopamine agonist	2 (3.4)
Other^d^	14 (24.1)

Values represented as n (%), unless stated otherwise.

a Percentages calculated based on the total number of women in the study (N).

b Percentages calculated among participants with an OHSS.

c Percentages calculated among participants with at least one preventive intervention of early OHSS. More than one preventive intervention can be selected, so the total number of participants may not correspond to the sum of each category.

d This category includes administration of dalteparin or triggering of final follicular maturation with hCG and with cryopreservation of all blastocysts.

GnRH, gonadotropin-releasing hormone; hCG, human chorionic gonadotropin; N, number of participants; OHSS, ovarian hyperstimulation syndrome; SD, standard deviation.

### 3.7 Self-reported satisfaction with follitropin delta

**Figure 3:**
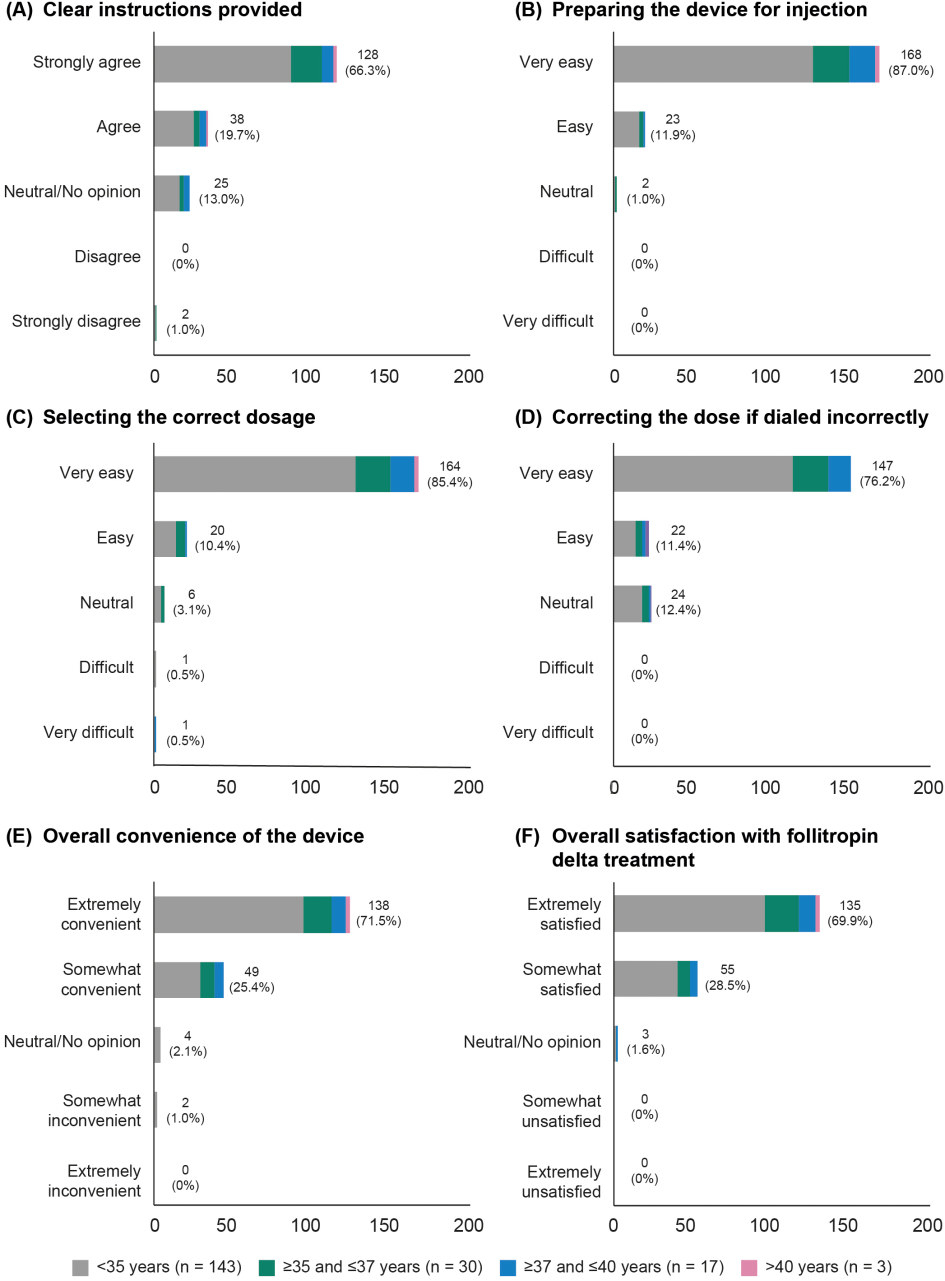
Patient overall satisfaction, categorized by age group. **(A)** Clear instructions provided. **(B)** Preparing the device for injection. **(C)** Selecting the correct dosage. **(D)** Correcting the dose if dialed incorrectly. **(E)** Overall convenience of the device. **(F)** Overall satisfaction with follitropin delta treatment.

Nearly all participants (193/199, 97.0%) completed the questionnaire (**Supplementary Table 1**). Among these, 128/193 (66.3%) women reported that they “strongly agreed,” and 38/193 (19.7%) women reported that they “agreed,” that the instructions provided with the pen were clear (**Figure 3**). There were 138/193 (71.5%) women who found the device “extremely convenient,” while 49/193 (25.4%) reported it to be “somewhat convenient.” Additionally, 135/193 (69.9%) women expressed being “extremely satisfied” with follitropin delta treatment, while 55/193 (28.5%) women reported being “somewhat satisfied” (**Figure 3**). These patterns were consistently observed across all age groups.

## 4 Discussion

The NORSOS study documented the use of follitropin delta in women undergoing their first IVF/ICSI cycle in a general clinical setting. The findings revealed that 47.9% (93/194) of participants achieved the algorithm-targeted ovarian response of 8–14 oocytes retrieved, which aligned with RCTs and other observational studies. The incidence of OHSS was low, and patient satisfaction with the follitropin delta pre-filled pen was high.

Follitropin delta is the first FSH preparation to utilize an approved algorithm for calculating individualized daily dosing based on a woman’s body weight and serum AMH concentration. In this study, 169/199 (84.9%) participants received treatment with a fixed daily dose, calculated using the dosing algorithm. The mean starting dose was 10.2 µg, comparable with the PROFILE study (10.4 µg) and slightly lower than the dose reported in the DELTA study (11.4 µg) (16, 17). Only a small proportion of participants (12/199, 6.0%) had their starting dose adjusted during ovarian stimulation, with one-third of those having an increase in dose and two-thirds having a decrease. A total of 171/193 (88.6%) participants received luteinizing hormone surge suppression with GnRH antagonist. The limited use of GnRH agonist therapy agrees with recent evidence showing that agonist and antagonist approaches can reach similar efficacy, but GnRH antagonist treatment is often preferred as it is associated with a lower incidence of OHSS (18).

Ovarian response rates in the NORSOS study were in line with other European observational studies. The mean number of oocytes retrieved per woman in this study was 12.1, similar to what was previously reported in the PROFILE and DELTA studies (16, 17). A normal ovarian response (8–14 oocytes) after a fresh transfer was achieved by 93/194 (47.9%) of women, while a high response (>15 oocytes) was observed in 55/194 (28.4%) of this study’s participants. These findings align with the ovarian response rates reported in the prospective DELTA study, where 46.1% of French women reached the target response of 8–14 oocytes (17). Similarly, a retrospective sub-analysis conducted in Germany documented that 42.1% of women achieved the target response after individualized follitropin delta treatment (19).

Being a non-interventional study, NORSOS study sites were free to implement their own clinical approaches based on local clinical practices, from freeze-all strategy to cycle cancellation. In this study, a freeze-all strategy was implemented for 70/194 (36.1%) of the enrolled women. These results were slightly higher than those previously reported (16, 17), which might be explained by differences observed in patient characteristics between populations. In the current study, the mean participant age was 32.0 years, with 147/199 (73.9%) of women being under 35 years of age. Participants were slightly younger than those enrolled in the non-interventional PROFILE and DELTA studies (mean age: 33.5 and 33.0 years, respectively) (16, 17). Our study also included a higher proportion of women with a median AMH concentration ≥15 pmol/L (130/199, 65.3%) compared with the PROFILE (55.0%) and DELTA (59.9%) cohorts (16, 17). This, combined with the younger age of the NORSOS participants, might have led to the decision to implement a higher proportion of freeze-all strategies as a more cautious approach to mitigate any potential risk of OHSS. These findings illustrate how variations in patient characteristics, along with country-specific clinical practice, may contribute to differences in the clinical approaches used at the sites. Nonetheless, real-world evidence shows that despite these differences, follitropin delta treatment ultimately leads to adequate ovarian responses (42.1–46.1% of women retrieving 8–14 oocytes) and good clinical pregnancy rates per embryo transfer (30.7–38.2%) (16, 17, 19).

In the present study, among the 155/199 (77.9%) women who underwent at least one transfer (fresh and/or frozen), around half had a fresh transfer (106/199, 53.3%), and one-quarter underwent a first frozen transfer (49/199, 24.6%). While the total number of women undergoing embryo transfer was comparable, differences in proportions of fresh transfers and pregnancy outcomes were observed compared with other European studies. These differences are likely attributable to the previously mentioned variations in patient characteristics and the specific clinical practices unique to each country. The proportion of fresh transfers reported in the present study was lower than that of the PROFILE and DELTA studies (62.7% and 77.6% respectively) (16, 17). On the other hand, overall rates per transfer of ongoing pregnancy in participants with a fresh transfer in the NORSOS study were relatively higher (43.4% and 40.6%, respectively) compared with those reported in the PROFILE study (30.7% and 27.0%, respectively). For women undergoing any transfers (frozen and/or fresh) after ovarian stimulation with follitropin delta, the current study reported an ongoing pregnancy rate of 82/155 (52.9%), which was also particularly high compared with the ongoing pregnancy rates described for French women in the DELTA trial (29.6% per initiated cycle). Overall, pregnancy loss (i.e., spontaneous abortion, biochemical pregnancy) in participants with a fresh transfer was reported in 12/106 (11.3%) women; this was lower than existing retrospective-based evidence on general miscarriage rates after IVF/ICSI treatment, which ranged from 13% to 20% (17, 20, 21).

As for all other clinical procedures, the NORSOS study protocol did not specify any criteria for determining the application of preventive measures for OHSS risk, leaving each clinician free to implement their own clinical approach on preventive interventions. This approach was aligned with the 2024 American Society for Reproductive Medicine guidelines (22), which support clinician-led decisions for OHSS risk mitigation, even if not uniformly applied across all sites. This flexibility ensured that the study reflects routine clinical practice as accurately as possible. In addition, recent international guidelines established a threshold of >15–18 oocytes collected to indicate any risk of OHSS, while a retrospective analysis from the Swedish National Quality Registry of Assisted Reproduction (Q-IVF) indicated a cut-off of >18 oocytes as a risk for severe OHSS (22, 23). In this study, where 29.1% (58/199) of participants received preventive measures for OHSS, only 14.6% (29/199) retrieved >15 oocytes, and an even smaller proportion (10.6%, 21/199) retrieved >18 oocytes. These findings align with previously described observational evidence (PROFILE: 16.5%; DELTA: 14.8%) (16, 17), highlighting that the decision to apply preventive measures for OHSS below the recommended thresholds is primarily at the clinician’s discretion.

The NORSOS study is the first to assess patient-reported satisfaction with follitropin delta treatment in a general clinical setting. Overall, most participants were extremely satisfied with follitropin delta treatment (135/193, 69.9%) and found the device extremely convenient to use (138/193, 71.5%). In comparison, a previous questionnaire-based study conducted in Japanese women reported an overall satisfaction rate of approximately 80.0% with follitropin alpha, with a similar proportion of women finding the follitropin alpha injection pen easy to use (75.1%) (24). It should be noted that the NORSOS study questionnaire did not include a comparator since women were in their first assisted reproductive technology cycle with no prior pen experience, whereas the Japanese-based study did include comparators. These findings suggest that follitropin delta ensures a level of patient satisfaction and ease of administration comparable with other gonadotropins, which are crucial factors for improving compliance and optimizing treatment outcomes.

The NORSOS study investigated the usage, effectiveness, and safety profile of follitropin delta through a prospective design with primary data collection, lowering the risk of missing data compared with other assisted reproductive technology studies that used a retrospective review of electronic medical records. This study utilized observational data from different countries, with clinical decisions made at the discretion of the treating physician, reflecting local practices. The heterogeneous nature of the study population mirrors a broader patient population, which supports the generalizability of the findings. However, there are some limitations to consider when interpreting this study’s results. First, the population mainly included young women under 35 years of age, limiting generalizability to women of more advanced fertile ages, especially those over 40 (n = 3). This age distribution may partly reflect real-world access criteria, as many clinical settings can often impose age and BMI-related restrictions, even though the study itself did not apply such exclusions. Second, while greater heterogeneity in study participants reflects broader patient populations, differences in patient characteristics between this study and other observational studies or RCTs have led to variations in outcomes like freeze-all strategy, fresh embryo transfer cancellations, and OHSS preventive measures, complicating cross-study comparisons. Third, as with all observational studies, owing to their reliance on routinely collected clinical data, some variables may be missing or inconsistently recorded, and differences in treatment decisions made by physicians may further contribute to biased results. Finally, the use of an *ad hoc* questionnaire to assess patient satisfaction may affect the reliability of the findings. Future research should use validated questionnaires to confirm and complement these results.

### 4.1 Conclusion

This prospective study further supports the use of follitropin delta in routine clinical practice, complementing data from previous randomized clinical studies conducted in other countries and highlighting the importance of individualized dosing based on each patient’s characteristics to optimize IVF/ICSI outcomes. Most participants received follitropin delta for ovarian stimulation according to the algorithm-recommended dose, with only a few dose deviations occurring during stimulation. Nearly half of the participants had 8–14 oocytes retrieved, with a similar proportion of women who underwent a fresh embryo transfer achieving clinical pregnancy rates comparable with those observed in clinical trials. These findings, combined with no additional safety concerns reported and a low OHSS incidence, suggest the effectiveness and positive safety profile of individualized follitropin delta dosing, supporting its use in routine clinical practice and offering a flexible and patient-friendly approach to ovarian stimulation. The study also assessed, for the first time, patient-reported satisfaction with follitropin delta, revealing that most women considered the pre-filled pen convenient and were satisfied with it overall. Future studies should aim to include a broader age range and diverse patient populations.

## Data Availability

The original contributions presented in the study are included in the article/**Supplementary Material**. Further inquiries can be directed to the corresponding author.
