# Supplementary material for: Effectiveness and safety of follitropin delta in routine clinical practice in the Nordics and Switzerland (the NORSOS study): a prospective non-interventional study
Source: Front Endocrinol (Lausanne). 2025 Sep 5;16:1613680. doi: 10.3389/fendo.2025.1613680 (PMC12446022; doi:10.3389/fendo.2025.1613680)
Supplement: Supplementary file 1 [file DataSheet1.pdf]

**Document No:**	25533
**Document Name:**	E-Study Protocol-25533(2.0)
**Document Title:**	FE 999049 000411 (NORSOS) Non-Interventional Trial Protocol
**Description:**	FE 999049 000411 (NORSOS) Non-Interventional Trial Protocol
**Document State:**	Effective
**Document Version:**	2.0
**Effective Date:**	27 Apr 2022 10:09:36 (GMT+01:00)
**Print Information:**	This is a controlled print from REAL

Details:
Owning Departments: Ferring
Cross Ref Departments:

Signatures:

Signed By : Lene Melchiorsen (LENM)
Decision : Approved
Decision Date : 13 Apr 2022 08:19:27 (GMT+02:00)
Role : Approver
Purpose : New update due to typo
Meaning Of Signature : I approve this document for use

Signed By : Pernille Hjarnaa (PEHJ)
Decision : Approved
Decision Date : 13 Apr 2022 13:41:48 (GMT+02:00)
Role : Approver
Purpose : New update due to typo
Meaning Of Signature : I approve this document for use

Signed By : Anette Oestergaard (ANOE)
Decision : Approved
Decision Date : 18 Apr 2022 17:40:24 (GMT+02:00)
Role : Approver
Purpose : New update due to typo
Meaning Of Signature : I approve this document for use

Signed By : Bjarke Mirner Klein (bemk)
Decision : Approved
Decision Date : 18 Apr 2022 20:54:40 (GMT+02:00)
Role : Approver
Purpose : New update due to typo
Meaning Of Signature : I approve this document for use

Signed By : Bernadette Mannaerts (btm)
Decision : Approved
Decision Date : 27 Apr 2022 10:09:30 (GMT+02:00)
Role : Approver
Purpose : New update due to typo
Meaning Of Signature : I approve this document for use

Printed For: **Isabel Kristoffersen (ISKR)** By **Isabel Kristoffersen (ISKR)**
Print Reason: **External Distribution**

Printed On: 27 Apr 2022 (GMT+02:00) @ 1:44:51 PM (GMT+02:00) **Print Type:** No Watermark Print

NON-INTERVENTIONAL TRIAL PROTOCOL

Nordics and Switzerland Prospective Multicentre Non-Interventional Observational Trial to Assess the Pattern of Use of REKOVELLE® in Women Undergoing In Vitro Fertilisation (IVF) or Intracytoplasmic Sperm Injection (ICSI) Procedures in Routine Clinical Practice

Trial Code: 000411

(NORSOS)

**Medicinal Product:**	*Follitropin delta (REKOVELLE®)*
**Indication:**	*Controlled ovarian stimulation (COS) for the development of multiple follicles in women undergoing assisted reproductive technologies (ART) such as an in vitro fertilisation (IVF) or intracytoplasmic sperm injection (ICSI) cycle.*
**Phase:**	*Phase 4, Non-Interventional Trial*
**Name and Address of Sponsor:**	*Ferring International Center SA*<br>*Chemin de la Vergognausaz 50*<br><br>*CH-1162, Saint-Prex*<br>*Switzerland*

The information in this document is confidential and is proprietary to Ferring Pharmaceuticals A/S or another company within the Ferring Group. It is understood that information in this document shall not be disclosed to any third-party, in any form, without prior written consent of an authorised officer of Ferring Pharmaceuticals A/S or another company within the Ferring Group.

PROTOCOL OUTLINE

TRIAL TITLE

Nordics and **S**witzerland, Prospective Multi-centre Non-Interventional **O**b**S**ervational Trial to Assess the Pattern of Use of REKOVELLE® in Women Undergoing In Vitro Fertilisation (IVF) or Intracytoplasmic Sperm Injection (ICSI) Procedures in Routine Clinical Practice (NORSOS)

TRIAL SITES

15 active sites in Sweden, Denmark, Norway, Switzerland

Trial coordinator: Dr. Ani Azroyan, Ph. D

Address: 7 rue Jean Baptiste Clement, 94250 Gentilly

Email: ani.azroyan@ferring.com

PLANNED TRIAL PERIOD

The total duration of the trial will be approximately 24 months (from Ethics Submission to LSLV).

Trial Milestone	Estimated Date
Ethics submission	Q2 2022
Site Initiation visits	Q3 2022
Start of data collection (first subject first visit)	Q3 2022
Last subject inclusion	Q4 2023
End of data collection (last subject last visit)	Q1 2024
Data base lock	Q1 2024
Final Trial Report	Q2 2024

OBJECTIVES

Primary objective

To observe the usage patterns of REKOVELLE®, in women naïve to in vitro fertilisation (IVF) and intracytoplasmic sperm injection (ICSI), undergoing their first COS treatment cycle with REKOVELLE® in routine clinical practice.

Secondary objectives

- To observe the ovarian stimulation and embryo development outcomes of REKOVELLE® in routine clinical practice after the 1st treatment cycle.
- To observe the safety profile of REKOVELLE® in routine clinical practice after the 1st treatment cycle.

- To observe the subject satisfaction of overall REKOVELLE® treatment and REKOVELLE® pre-filled pen usage in naïve subjects in routine clinical practice after 1st treatment cycle.

ENDPOINTS

Primary endpoint

Treatment patterns in first cycle with REKOVELLE®:

- Use of the algorithm-based individualised dosing regimen (daily dose of REKOVELLE® administered), number of days of treatment, dose changes; use of the Algorithm dosing Application; use of GnRH protocol, type of GnRH protocol, triggering of follicle maturation; luteal phase support.

Secondary endpoints

Ovarian stimulation and embryo development outcomes of REKOVELLE® in routine clinical practice:

- Rate of ongoing pregnancy (≥1 intrauterine viable fetus 10-11 weeks after embryo transfer) in the fresh cycle
- Rate of ongoing pregnancy (≥1 intrauterine viable fetus 10-11 weeks after embryo transfer) following the fresh cycle, including also the first frozen embryo transfer
- Number of oocytes retrieved
- Number of oocytes/embryos/blastocysts frozen
- Number of embryos/blastocysts transferred
- Quality of fresh embryos or blastocysts transferred (excellent, good, fair, other)
- Positive signs of pregnancy (hCG test or urine pregnancy test) and clinical pregnancy (at least one gestational sac 5-6 weeks after transfer)
- Pregnancy loss in women with embryo/blastocyst transfer (biochemical pregnancy, spontaneous/elective abortion, ectopic pregnancy, vanishing twin)
- Cycle cancellation before or after oocyte pick-up and reason for cycle cancellation

Safety profile of REKOVELLE® in routine clinical practice:

- Adverse drug reactions (ADRs)
- OHSS (including mild/moderate/severe)
- Preventive interventions for early ovarian hyperstimulation syndrome (OHSS): Triggering of final follicular maturation with GnRH agonist, cancellation of fresh embryo/blastocyst transfer

Subject satisfaction with REKOVELLE® in routine clinical practice:

- Assessment of overall subject experience and convenience with REKOVELLE® by the Subject Questionnaire (assessed by the subject)

METHODOLOGY

This is a European trial including Nordic Countries (Sweden, Denmark, Norway) and Switzerland. A Prospective, multi-centre, post-authorisation, non-interventional observational cohort trial conducted under routine clinical practice in fertility clinics to collect information from 200 naïve women treated with REKOVELLE® and undergoing IVF or ICSI procedures.

As the trial is non-interventional, the decision to treat subjects with REKOVELLE® will be independent from the decision to enrol the subject into the trial. Subjects will be enrolled only after the treatment decision has been made and no aspect of this trial will interfere with or influence the routine medical procedures and/or medications received. No trial medications will be provided.

In each site, during the enrolment period, consecutive women who are prescribed REKOVELLE® for IVF/ICSI cycle according to the approved label, will be invited to participate in the trial. The trial enrolment will be monitored, and the enrolment will be locked once 200 women have initiated treatment. Each woman will contribute only for one stimulation cycle over the observation period.

During the observation period, the investigators will collect data for 1 stimulation cycle with REKOVELLE® or until early withdrawal. Data about cycles using frozen embryos/blastocysts will be collected only for the first transfer cycle if they are performed within 3 months after the cycle stimulated with REKOVELLE® within the observation period.

Women with embryo/blastocyst transfer will be followed until ongoing pregnancy (approximately 10-11 weeks after the first fresh or frozen transfer), or no pregnancy is confirmed or early pregnancy loss, or trial withdrawal. Ultrasound results must be obtained if not performed at the participating site.

Baseline, COS treatment and follow-up assessments will be performed by the investigators during routine local clinical care visits.

Subject questionnaire will be completed by the subjects at the clinic after cycle cancellation, trial withdrawal or before the oocyte retrieval.

NUMBER OF SUBJECTS

There will be 200 subjects initiated with REKOVELLE® treatment and enrolled (signs Informed Consent Form (ICF) to participate in the study)

CRITERIA FOR INCLUSION / EXCLUSION

Subjects who meet **all** of the following inclusion criteria and who do not meet **any** of the following exclusion criteria are eligible for participation.

Inclusion criteria

- Females aged 18 years or older at enrolment
- Who have never been treated with (naïve) *in vitro* fertilisation (IVF) or intracytoplasmic sperm injection (ICSI) treatment using fresh or frozen ejaculated sperm from male partner or sperm donor
- Who are prescribed REKOVELLE® for the first time, using the dosing algorithm-based on AMH test result and body weight to define the 1st cycle dose regimen with REKOVELLE® according to the approved label
- Who have been informed verbally and in writing about this trial content, signed the inform consent and who do not object to their data being electronically processed

Exclusion criteria

- Currently participating in an interventional clinical trial in which any treatment or follow-up is mandated
- Women with a contraindication for prescription of REKOVELLE® treatment
    - Hypersensitivity to the active substance or to any of the excipients
    - Tumours of the hypothalamus or pituitary gland
    - Ovarian enlargement or ovarian cyst not due to polycystic ovarian syndrome
    - Gynaecological haemorrhages of unknown aetiology
    - Ovarian, uterine, or mammary carcinoma
    - Primary ovarian failure
    - Malformations of sexual organs incompatible with pregnancy
    - Fibroid tumours of the uterus incompatible with pregnancy
    - Pregnancy and breast feeding
- Women who undergo ovarian stimulation for fertility preservation
- Women placed under judicial protection, guardianship, or supervision
- Women who are considered as vulnerable population

MEDICINAL PRODUCT

REKOVELLE® (follitropin delta), solution for injection - delivered in a pre-filled injection pen.

Indicated dose according to approved label. Subjects must be treated in the usual manner in accordance with the terms of the marketing authorisation.

The REKOVELLE® dose should be based on a recent determination of body weight and AMH (i.e., within the last 12 months) measured by one of the alternative assays for dose selection; ELECSYS AMH Plus Immunoassay from Roche, or ACCESS AMH Advanced from Beckman Coulter or LUMIPULSE G AMH from Fujirebio.

DURATION OF PARTICIPATION

Women will be enrolled in each site over the enrolment period and will contribute for one stimulation cycle of REKOVELLE® in routine clinical practice, over the observation period. Women with embryo/blastocyst transfer will be followed until the ongoing pregnancy (approximately 10-11 weeks after the first fresh or frozen transfer).

First transfer cycles with frozen embryo (or blastocyst) will be followed if they are performed within 3 months after the stimulation cycle with REKOVELLE® within the enrolment period.

Non-pregnant women will be followed for 1 stimulation cycle with REKOVELLE® or until early withdrawal.

STATISTICAL METHODS

Statistical analysis

This is a descriptive trial. There are no hypotheses to be tested and no inferential statistics will be applied. All descriptions of data will be based on all subjects enrolled in the trial who meet the inclusion/exclusion criteria and who receive at least one dose of REKOVELLE®. Therefore, pregnancy rates will be calculated for all subjects starting stimulation with REKOVELLE®, and mean number of oocytes, embryos, blastocysts etc. will be the mean for all subjects starting stimulation with REKOVELLE®.

Missing data for pregnancy outcomes will be regarded as “no pregnancy”. Missing data for number of oocytes, number of embryos, number of blastocysts, and similar endpoints will be regarded as zero.

The primary endpoint, real-life treatment patterns in first cycle with REKOVELLE® will be extensively described.

Sample size

The sample size is not based on any inferential statistical considerations. This trial will be conducted in Nordic Countries (Sweden, Denmark, Norway) and Switzerland in Europe, with the activation of 15 sites, known to follow-up women undergoing ART and to regularly prescribe REKOVELLE®. Each site will enrol maximum 15 subjects during the predefined inclusion period, until the total 200 naïve women initiated with REKOVELLE® treatment are enrolled and enrolment target is achieved in a competitive manner.

Considering the total number of fertility centres in those countries and knowing that all these centres prescribe REKOVELLE®, the 15 sites expected to participate in the trial should be sufficiently representative to reflect the variety of practices in those contributing European Countries.

TABLE OF CONTENTS

PROTOCOL OUTLINE 2
TABLE OF CONTENTS 7
LIST OF TABLES AND FIGURES INCLUDED IN THE TEXT 9
LIST OF ABBREVIATIONS AND DEFINITIONS 10
1 INTRODUCTION 12
1.1 Background 12
1.2 Trial Rationale 14
2 TRIAL OBJECTIVES 14
2.1 Primary Objective 14
2.2 Secondary Objectives 14
3 ENDPOINTS 15
3.1 Primary Endpoint 15
3.2 Secondary Endpoints 15
4 METHODS 15
4.1 Trial Design 15
4.2 Medicinal Product 17
4.3 Limitations of Trial Design 17
4.4 Scientific Committee 18
5 SELECTION OF TRIAL POPULATION 18
5.1 Trial Population 18
5.2 Subject Eligibility 18
5.2.1 Inclusion Criteria 18
5.2.2 Exclusion Criteria 19
5.3 Trial Withdrawal/Treatment Discontinuation Criteria 19
5.4 Site Selection 19
5.5 Duration of Trial 20
6 ASSESSMENTS 20
6.1 Schedule of Assessments 20
6.2 Data collected in the e-CRF 22
6.2.1 Baseline Data 22
6.2.2 COS Treatment Data 22
6.2.3 Follow-up 23
6.2.4 Trial Termination 23
6.2.5 Subject Questionnaire 24
7 SAFETY REPORTING 24
7.1 Definitions 24
7.1.1 Adverse Event 24
7.1.2 Serious Adverse Events 24
7.1.3 Causal Relationship to REKOVELLE® 25
7.2 Adverse Event Resulting in Fatal Outcome 26
7.3 Congenital Abnormality 26
7.4 Collection and Recording of Adverse Drug Reactions 26
7.5 Collection, Recording and Reporting of Serious Adverse Drug Reactions 28
7.6 Follow-up of Adverse Drug Reactions and Serious Adverse Drug Reactions 29

7.6.1 Follow-up of Adverse Drug Reactions ... 29

8 STATISTICAL METHODS ... 29
8.1 Sample Size ... 29
8.2 Statistical Analysis ... 29
8.2.1 General Considerations ... 29
8.2.2 Analysis Sets ... 30
8.3 Data Analysis ... 30
8.3.1 Subject Disposition ... 30
8.3.2 Demographics and Other Baseline Characteristics ... 30
8.3.3 Analysis of the Primary Endpoint ... 30
8.3.4 Analysis of the Secondary Endpoints ... 30

9 DATA HANDLING ... 32
9.1 Data Abstraction ... 32
9.2 Electronic Case Report Forms and Data Capture System ... 32
9.3 Subject Questionnaire ... 32
9.4 Data Management Plan ... 33
9.5 Data Coding ... 33

10 MONITORING PROCEDURES ... 33
10.1 Training ... 33
10.2 Periodic Monitoring ... 33
10.3 Confidentiality of Subject Data ... 33

11 CHANGES IN CONDUCT OF THE TRIAL ... 34
11.1 Protocol Amendments ... 34
11.2 Premature Termination of Trial Sites ... 34

12 REPORTING AND PUBLICATION ... 34
12.1 Non-interventional Trial Report ... 34
12.2 Confidentiality and Ownership of Trial Data ... 34
12.3 Publication Policy ... 34
12.4 Public Disclosure Policy ... 35

13 ETHICAL AND REGULATORY ASPECTS ... 35
13.1 Institutional Review Board (IRB)/Independent Ethics Committee Approval (IEC) .. 35
13.2 Regulatory Authority Authorisation/Approval/Notification ... 35
13.3 End of Trial ... 35
13.4 Subject Information and Consent ... 36
13.5 Data Collected on Investigators ... 36

14 LIABILITIES AND INSURANCE ... 36
14.1 Compliance Reference Documents ... 36
14.2 Liabilities and Insurance ... 36

15 ARCHIVING ... 37
15.1 Site File ... 37
15.2 Trial Master File ... 37

16 REFERENCES ... 37

17 APPENDICES ... 40
Appendix 1 LIST OF CONTACTS ... 40
Appendix 2 SUBJECT QUESTIONNAIRE ... 41

LIST OF TABLES AND FIGURES INCLUDED IN THE TEXT

TABLE 1 TRIAL FLOWCHART 20

LIST OF ABBREVIATIONS AND DEFINITIONS

ADR	Adverse Drug Reaction
AE	Adverse Event
AMH	Anti-Müllerian Hormone
ART	Assisted Reproductive Technologies
ATC	Anatomical Therapeutic Chemical
COS	Controlled Ovarian Stimulation
CRO	Clinical Research Organization
e-CRF	electronic Case Report Form
EDC	Electronic Data Capture
FET	Frozen Embryo/blastocyst Transfer
FSH	Follicle Stimulating Hormone
GnRH	Gonadotropin-Releasing Hormone
GPP	Good Pharmacoepidemiology Practice
hCG	human Chorionic Gonadotropin
ICF	Informed Consent Form
ICMJE	International Committee of Medical Journal Editors
ICSI	IntraCytoplasmic Sperm Injection
IEC	Independent Ethics Committee
IRB	Institutional Review Board
IU	International Unit
IVF	In Vitro Fertilisation
LH	Luteinizing Hormone
MedDRA	Medical Dictionary for Regulatory Activities
OHSS	Ovarian Hyperstimulation Syndrome
rFSH	recombinant Follicle Stimulating Hormone
SADR	Serious Adverse Drug Reactions
SAP	Statistical Analysis Plan
WHO	World Health Organization
Total Trial Period	Total trial duration time for the trial (approximately 2 years from First Patient First Visit (FPFV) to Last Patient Last Visit (LPLV).
Enrolment period	Period in which a given site may enrol new subjects (after the completion of the subject consent process) and may capture data for subject's 1st REKOVELLE® cycles)

Observation period	For a subject:<br>• From informed consent through 1st cycle with REKOVELLE®;<br>• In case of pregnancy, up to 11 weeks gestation (pregnancy follow-up)<br><br>For a site:<br>Enrolment period+ subject observation period (through ongoing cycle and pregnancy follow-up if applicable)
Controlled Ovarian Stimulation Period<br>(Refer to Table 1)	Stimulation preparation and stimulation period until oocyte retrieval.
Follow-up up period<br>(Refer to Table 1)	From oocytes retrieval through<br>• 11-week pregnancy outcomes, or<br>• Negative hCG result, or<br>• Negative clinical pregnancy<br>• Pregnancy loss<br>• Cycle cancellation or<br><br>Frozen embryo transfer cycle(s) performed within 3 months after the stimulation cycle with REKOVELLE® within the enrolment period
Trial Withdrawal	Discontinuation of subject's participation in the trial irrespective of the choice, reasons for discontinuation.
Treatment discontinuation	Subjects who discontinue REKOVELLE® or IVF/ICSI.
Lost to follow-up up	Refers to Subjects who were enrolled, signed ICF and actively participating in the study but have become lost at follow-up.
Cycle cancellation	Cycle cancelled before or after oocyte pick-up-up or Transfer cancellation.
Freeze All strategy	Decision to freeze all oocytes or embryos

1 INTRODUCTION

1.1 Background

Infertility is often defined as the failure to achieve a pregnancy after 12 months or more of regular unprotected sexual intercourse. Many factors (age, gynaecological problems, lifestyle factors) can cause infertility and may involve the male, the female or both. Global estimates suggest that the 12-month prevalence rate of infertility ranged from 3.5% to 16.7% in developed countries, with a median prevalence of 9% worldwide for women aged 20 - 44 years. Mascarenhas et al. estimated that 1.9% of child-seeking women aged 20 - 44 years experienced primary infertility (unable to have a live birth) and 10.5% secondary infertility (at least one live birth). The proportion of infertile couples seeking any infertility medical care ranges from 51% in less developed countries to 56% in developed countries, and 22% actually received infertility treatment.

Among fertility treatment, controlled ovarian stimulation (COS) with recombinant or urinary follicle stimulating hormone (FSH) and human menotropin gonadotropin (hMG) aims to obtain an adequate number of competent oocytes to be used for assisted reproductive technologies (ART) such as an in vitro fertilisation (IVF) or intracytoplasmic sperm injection (ICSI), with minimum risks for the woman.

The ovarian response is influenced by the dose of gonadotropin, but there is a large variability across subjects for the same dose of gonadotropin. A standard starting dose of gonadotropin in women with a low ovarian reserve may result in a poor ovarian response. In women with a high ovarian reserve, the same dose may result in an excessive response and therefore increases the risk of complications such as the ovarian hyperstimulation syndrome (OHSS). OHSS is a rare but critical complication associated with gonadotropin use. Severe OHSS occurs in approximately 1.4 % of all COS cycles.

Individualizing COS regimens is therefore crucial to ensure an appropriate dosing from the start of stimulation to reduce the risk of cycle cancellation due to poor response and minimize the iatrogenic risks due to an excessive response. The use of biomarkers, which can predict ovarian response to exogenous FSH stimulation, has been extensively investigated. Among the different biomarkers, the serum level of anti-Müllerian hormone (AMH) is currently considered as the most robust marker of the ovarian reserve. In addition, AMH serum levels show relative stability and consistency during the menstrual cycle and can therefore be measured at any time of the menstrual cycle.

In December 2016, Ferring received Marketing Authorisation approval from the European Commission for follitropin delta (FE 999049) under the trade name REKOVELLE®, a new human recombinant FSH (rFSH) available in pre-filled pen. The indication is: “COS for the development of multiple follicles in women undergoing ART such as an in vitro fertilisation (IVF) or intracytoplasmic sperm injection (ICSI) cycle.” This is the first rFSH treatment to be administered with an individualised dosing regimen, based on a woman's serum AMH level as well as her body weight.

This individualised dosing regimen was established in a phase-2 AMH-stratified trial conducted in 265 IVF/ICSI subjects using pharmacokinetic (PK) and pharmacodynamic (PD) modelling and simulation. A robust assay was developed in collaboration with Roche Diagnostics to ensure a reliable assessment of AMH levels at the standards intended for companion diagnostics.

The efficacy and safety of follitropin delta was first of all evaluated in the ESTHER-1 (Evidence-based Stimulation Trial with Human rFSH in Europe and Rest of World) phase-3 trial, which compared the individualized follitropin delta dosing based on AMH level and body weigh with conventional follitropin alfa (Gonal-F®) dosing (IU). Subjects who did not achieve an ongoing pregnancy could continue in the ESTHER-2 phase-3 trial, which evaluated the immunogenicity in repeated COS cycles. The results of ESTHER-1 showed that individualized follitropin delta was non-inferior to conventional follitropin alfa for ongoing pregnancy and ongoing implantation rates (co-primary endpoints). Overall, for secondary endpoints, individualized follitropin delta resulted in more women with target response (8-14 oocytes) (43.3% vs. 38.4%), with fewer cases of extreme ovarian responses, and a reduced need for OHSS preventive measures.

Phase 2 and Phase 3 STORK trial established the efficacy and safety of ovarian stimulation with individualised follitropin delta dosing based on serum anti-Müllerian hormone (AMH) and body weight versus conventional follitropin beta dosing in a population of subjects in Japan. In the Phase 3 trial non-inferiority was established between the individualized follitropin delta (AMH <15 pmol/l: 12 µg/day; AMH ≥15 pmol/l: 0.10–0.19 µg/kg/day; minimum 6 µg/day; maximum 12 µg/day) and conventional follitropin beta (150 IU/day for the first 5 days, with potential subsequent dose adjustments) based on number of oocytes retrieved. The individualised follitropin delta dosing approach demonstrated a favourable benefit-risk profile providing a statistically significant and clinically relevant reduction in the incidence of OHSS, without compromising live birth rates.

Randomised, controlled, multi-centre, assessor blind Phase 3 GRAPE Trial, conducted in 1009 Asian subjects from mainland China, South Korea, Vietnam, Taiwan, undergoing their first IVF/ICSI cycle established non-inferior ongoing pregnancy rate, a significantly higher live birth rate and a significantly lower incidence of early ovarian hyperstimulation syndrome (OHSS) and/or preventive interventions with individualised follitropin delta dosing compared to conventional follitropin alfa dosing.

A post-hoc analysis of the ovarian response data from IVF/ICSI subjects included in two randomized, assessor- blind, controlled trials in the development programme for follitropin delta run to determine the daily follitropin delta dose that would provide the same ovarian response as 150 IU of follitropin alfa (11 mcg defined in the Gonal-f SPC): a phase II dose-response trial and a phase III efficacy trial. Analysis of two independent datasets comparing ovarian response in IVF/ICSI subjects undergoing a GnRH antagonist protocol established that a daily dose of 10 µg follitropin delta provides a similar ovarian response to 150 IU/day follitropin alfa.

The Roche ELECSYS Plus AMH immunoassay was the first companion diagnostic approved for individualisation of the gonadotrophin starting dose for ovarian stimulation and is stipulated in the label for follitropin delta in accordance with regulatory guidelines, since its approval.

EMA approved an update (Type II variance) to the follitropin delta label by January 2021 including the use of two more alternative assays for algorithmic dose selection – 'ACCESS AMH Advanced' from Beckman Coulter, and LUMIPULSE G AMH' from Fujirebio.

1.2 Trial Rationale

The phase III clinical programs have demonstrated that REKOVELLE® is an effective and well-tolerated treatment for COS. Nevertheless, while clinical trials provide crucial information about drug efficacy and safety under controlled conditions in a selected group of subjects, broader information is needed to explore how the individualized dosing regimen of REKOVELLE® is used in routine clinical practice and to investigate the ovarian stimulation and embryo development outcomes and safety of REKOVELLE® under real-world conditions. Non-interventional, observational studies PROFILE and DELTA were designed to observe efficacy and safety of REKOVELLE®, describing the use of algorithm for the dose selection during 1st cycle and subject satisfaction with the pen usage, under real-life conditions [PROFILE (Prospective Multicentre non-Interventional Study to Assess the Patterns of Use of REKOVELLE® in Women Undergoing In Vitro Fertilisation or Intracytoplasmic Sperm Injection Procedures in Routine Clinical Practice. ClinicalTrials.gov Identifier: NCT03393780,) and DELTA (French Prospective Multicentre Non-Interventional Study to Assess the Design of Use of REKOVELLE® in Women Undergoing In Vitro Fertilisation or Intracytoplasmic Sperm Injection Procedures in Routine Clinical Practice. ClinicalTrials.gov Identifier: NCT04503707)].

The NORSOS trial will complement other Non-Interventional Studies, assessing use of REKOVELLE® in Women Undergoing In Vitro Fertilisation or Intracytoplasmic Sperm Injection Procedures in Routine Clinical Practice, under real-life conditions.

2 TRIAL OBJECTIVES

2.1 Primary Objective

To observe the usage patterns of REKOVELLE®, in women naïve to in-vitro fertilisation (IVF) and intracytoplasmic sperm injection (ICSI), undergoing their first COS treatment cycle with REKOVELLE® in routine clinical practice.

2.2 Secondary Objectives

- To observe the ovarian stimulation and embryo development outcomes of REKOVELLE® in routine clinical practice after the 1st treatment cycle.
- To observe the safety profile of REKOVELLE® in routine clinical practice after the 1st treatment cycle.
- To observe the subject satisfaction of overall REKOVELLE® treatment and REKOVELLE® pre-filled pen usage in naïve subjects in routine clinical practice after 1st treatment cycle.

3 ENDPOINTS

3.1 Primary Endpoint

Treatment patterns in first cycle with REKOVELLE®:

- Use of the algorithm-based individualised dosing regimen (daily dose of REKOVELLE® administered), number of days of treatment, dose changes ; use of the Algorithm dosing Application; use of GnRH protocol, type of GnRH protocol, triggering of follicle maturation; luteal phase support.

3.2 Secondary Endpoints

Ovarian stimulation and embryo development outcomes of REKOVELLE® in routine clinical practice will be observed:

- Rate of ongoing pregnancy (≥1 intrauterine viable fetus 10-11 weeks after embryo transfer) in the fresh cycle
- Rate of ongoing pregnancy (≥1 intrauterine viable fetus 10-11 weeks after embryo transfer) following the fresh cycle, including also the first frozen embryo transfer
- Number of oocytes retrieved
- Number of oocytes/embryos/blastocysts frozen
- Number of embryos/blastocysts transferred
- Quality of fresh embryos or blastocysts transferred (excellent, good, fair, other)
- Positive signs of pregnancy (hCG test or urine pregnancy test) and clinical pregnancy (at least one gestational sac 5-6 weeks after transfer)
- Pregnancy loss in women with embryo/blastocyst transfer (biochemical pregnancy, spontaneous/elective abortion, ectopic pregnancy, vanishing twin)
- Cycle cancellation before or after oocyte pick-up and reason for cycle cancellation

Safety profile of REKOVELLE® in routine clinical practice:

- Adverse drug reactions (ADRs)
- OHSS (including mild/moderate/severe)
- Preventive interventions for early ovarian hyperstimulation syndrome (OHSS): Triggering of final follicular maturation with GnRH agonist, cancellation of fresh embryo/blastocyst transfer

Subject satisfaction with REKOVELLE® in routine clinical practice:

- Assessment of overall subject experience and convenience with REKOVELLE® by the Subject Questionnaire (assessed by the subject)

4 METHODS

4.1 Trial Design

This is a European trial including Nordic Countries (Sweden, Denmark, Norway) and Switzerland. A Prospective, multi-centre, post-authorisation, non-interventional observational cohort trial conducted in routine clinical practice under real-life conditions in fertility clinics to

collect information from 200 naïve women treated with REKOVELLE® and undergoing IVF or ICSI procedures.

As the trial is non-interventional, the decision to treat subjects with REKOVELLE® will be independent from the decision to enrol the subject into the trial. Subjects will be enrolled only after the treatment decision has been made and no aspect of this trial will interfere with or influence the routine medical procedures and/or medications received. No trial medications will be provided.

In each site, during the enrolment period, consecutive women who are prescribed REKOVELLE® for IVF/ICSI cycle according to the approved label, will be invited to participate in the trial. The trial enrolment will be monitored, and the enrolment will be locked once 200 women initiated with REKOVELLE® have been enrolled. Each woman will contribute only for one stimulation cycle over the observation period.

During the observation period, the investigators will collect data for one stimulation cycle with REKOVELLE® or until early withdrawal. Data about transfer cycles using frozen embryos/blastocysts will be collected only for the first transfer cycle if they are performed within 3 months after the cycle stimulated with REKOVELLE® within the observation period.

Women with embryo/blastocyst transfer will be followed until the ongoing pregnancy (approximately 10-11 weeks after the first fresh or frozen transfer) or no pregnancy is confirmed or early pregnancy loss or trial withdrawal. Ultrasound results must be obtained if not performed at the participating site.

Baseline, COS treatment and follow-up assessments will be performed by the investigators during routine local clinical care visits.

Subject questionnaire will be completed by the subjects at the clinic after cycle cancellation, trial withdrawal or before the oocyte retrieval.

At the enrolment visit and for each eligible woman, the investigator will:

- Explain the trial to the woman, in particular, the objective of the trial and its epidemiological nature
- Give her the Informed Consent Form (ICF) and ask the subject to read and sign it to confirm her agreement to participate

The investigators will be requested to record information on enrolled women in the electronic Case Report Form (e-CRF) 1) at enrolment, 2) for the 1st cycle with REKOVELLE®, during subject follow-up at the time of routine clinic visits and 3) in case of trial withdrawal or treatment discontinuation.

The investigators will ask the enrolled women to complete the subject questionnaire on site at the clinic after cycle cancellation, trial withdrawal or before the oocyte retrieval.

In the event of trial withdrawal, the investigator will be asked to document the date and the reason for trial withdrawal (reasons to cancel participation in the trial and/or discontinue REKOVELLE® or IVF/ICSI cycle) in the e-CRF.

4.2 Medicinal Product

As the trial is non-interventional, the decision to treat subjects with REKOVELLE® will be independent from the decision to enrol the subject into the trial. Subjects will be enrolled only after the treatment decision has been made and no aspect of this trial will interfere with or influence the routine medical procedures and/or medications received. No trial medications will be provided.

REKOVELLE® (follitropin delta), solution for injection - delivered with a pre-filled injection pen, ATC code G03GA10, is a recombinant human FSH approved for the COS for the development of multiple follicles in women undergoing ART such as an in vitro fertilisation (IVF) or intracytoplasmic sperm injection (ICSI) treatments.

Subjects must be treated according to the routine clinical practice and REKOVELLE® must be prescribed in accordance with the terms of the marketing authorisation of each participating country.

According to the label the REKOVELLE® dose should be based on a recent determination of body weight and a recent determination of AMH (i.e. within the last 12 months) measured by the following diagnostic tests: ELECSYS AMH Plus immunoassay from Roche or alternatively the ACCESS AMH Advanced from Beckman Coulter or LUMIPULSE G AMH from Fujirebio.

4.3 Limitations of Trial Design

The trial is associated with some methodological limitations commonly found in observational studies:

- Selection Bias

Subjects will be included in the trial by approximately 15 sites known to follow-up women undergoing ART. The participating sites will be selected from private and public practice to represent all existing treatment policies and will be spread across the territory to represent as much as possible the variety of populations in the countries involved. This number of sites expected to participate in the trial may be too limited to be representative and to reflect the variety of practices in the countries involved.

Selection bias is a distortion of evidence or data that arises from the way data are collected. In order to limit bias in the selection of subjects, investigators will be asked to consecutively enrol all subjects who are prescribed REKOVELLE® for IVF/ICSI cycle, have signed inform consent and meet the selection criteria, regardless of other considerations.

To promote consecutive inclusions by investigators, this request will be regularly reminded. In addition, investigators will be asked to include all their eligible subjects to a limit of 15 during the predefined inclusion period, until the objective of 200 inclusions is reached. This procedure aims to have a representative sample of subjects treated with REKOVELLE®, based on real prescriptions, which could vary according to centres, while limiting a centre effect.

- Information Bias

Relying on investigators to fill out the assessment forms might induce the presence of missing data, which can result in bias. Entry of data via e-CRFs will minimise missing or incorrect data thanks to automated queries. Clear instructions and engagement with the trial staff, including appropriate training will minimise the amount of missing data.

- Confounding Bias

Confounding bias occurs when the effects of a treatment or the exposition effect of the disease vary by presence/level of another factor (effect modifier). Effect modifiers may be controlled by using stratification in the statistical analysis (subgroup analyses).

- Lost to follow-up

In order to assess the existence of attrition bias, the main baseline characteristics of subjects lost to follow-up will be contrasted with subjects having completed the trial. Because of the duration of the trial, the proportion of lost to follow-up subjects might not be high in proportion.

To understand the quality of life, impact, tolerability to the ART procedure with REKOVELLE® and subjects' experience of use of the REKOVELLE® Pen (ease of use, ease of training and administration) for fertility treatment, subjects will be administered a questionnaire at the clinic after cycle cancellation, trial withdrawal or before the oocyte retrieval.

Reasons for trial withdrawal will be also collected, and the characteristics of these subjects will be compared with the trial completers to detect any potential bias.

4.4 Scientific Committee

A Scientific Committee, composed by specialists in the field, will be responsible for providing advise related to trial data analysis and interpretation of results.

5 SELECTION OF TRIAL POPULATION

5.1 Trial Population

A total of 200 subjects who are initiated REKOVELLE® treatment for COS will be enrolled (signs ICF to participate in the study) in the trial.

This trial is intended to be conducted in a non-vulnerable population. No vulnerable subjects will be enrolled in the trial.

5.2 Subject Eligibility

Subjects who meet **all** of the following inclusion criteria and who do not meet **any** of the following exclusion criteria are eligible for participation:

5.2.1 Inclusion Criteria

- Females aged 18 years or older at enrolment

- Who have never treated with (naïve) in vitro fertilisation (IVF) or intracytoplasmic sperm injection (ICSI) treatment using fresh or frozen ejaculated sperm from male partner or sperm donor
- Who are prescribed REKOVELLE® for the first time, using the dosing algorithm-based on AMH test result and body weight to define the 1st cycle dose regimen with REKOVELLE®, according to the approved label
- Who have been informed verbally and in writing about this trial content, signed the inform consent and who do not object to their data being electronically processed

5.2.2 Exclusion Criteria

- Currently participating in an interventional clinical trial in which any treatment or follow-up is mandated
- Women with a contraindication for prescription of REKOVELLE® treatment
  - Hypersensitivity to the active substance or to any of the excipients
  - Tumours of the hypothalamus or pituitary gland
  - Ovarian enlargement or ovarian cyst not due to polycystic ovarian syndrome
  - Gynaecological haemorrhages of unknown aetiology
  - Ovarian, uterine, or mammary carcinoma
  - Primary ovarian failure
  - Malformations of sexual organs incompatible with pregnancy
  - Fibroid tumours of the uterus incompatible with pregnancy
  - Pregnancy and breast feeding
- Women who undergo ovarian stimulation for fertility preservation
- Women placed under judicial protection, guardianship, or supervision
- Women who are considered as vulnerable population.

5.3 Trial Withdrawal/Treatment Discontinuation Criteria

Participation in this trial is voluntary and women are free to withdraw from the trial at any time and for any reason without prejudice to their drug prescriptions or therapeutic management. Missing routine care visits at one or more time points or missing questionnaires will not be considered as criteria for Subject withdrawal.

If a woman withdraws from the trial, regardless of cause, the date and the reason for trial withdrawal will be documented in the e-CRF, if this information is available. Subjects who withdraw from the trial will be informed that data collected up to that point will be used in the trial analyses but that after withdrawal no further data will be collected for them.

5.4 Site Selection

This trial will be conducted in Nordic Countries (Sweden, Denmark, Norway) and Switzerland in Europe, at 15 sites known to treat and follow-up subjects undergoing COS and routinely prescribe REKOVELLE®. Fifteen IVF centres will be activated for the trial.

This trial will be proposed to public hospitals and private clinics, spread evenly across the territory, with the capacity to perform this observational trial and that use the ELECSYS AMH Plus Immunoassay from Roche or ACCESS AMH Advanced from Beckman Coulter or LUMIPULSE G AMH from Fujirebio to measure woman's serum AMH concentration.

5.5 Duration of Trial

The total duration of the trial will be approximately 24 months (from first subject first visit to data base lock). The duration of treatment is per investigators' judgement.

Trial Milestone	Estimated Date
Ethics submission	Q2 2022
Site Initiation visits	Q3 2022
Start of data collection (first subject first visit)	Q3 2022
Last subject inclusion	Q4 2023
End of data collection (last subject last visit)	Q1 2024
Data base lock	Q1 2024
Final Trial Report	Q2 2024

6 ASSESSMENTS

Due to the observational nature of the trial, all assessments will be performed per routine clinical practice.

6.1 Schedule of Assessments

Only data available in subject's records and needed to fulfil the trial objectives will be collected.

Visits will be done in accordance with routine clinical practice.

The flowchart of data collected by the investigators is provided in Table 1.

Table 1 Trial Flowchart

**Data**	**Baseline data**	**COS Cycle data**[@]	**Follow-up data** [$]
**Data Collected by the Investigators**			
Informed consent[§]	**X**		
Inclusion/exclusion criteria	**X**		
Socio-demographic data	**X**		
Infertility / menstrual / reproductive history	**X**		
Antral follicle count if available			**X**
Most recent AMH measurement (within the last 12 months) performed with ELECSYS AMH Plus Immunoassay from Roche or ACCESS AMH Advanced from Beckman Coulter or LUMIPULSE G AMH from Fujirebio	**X**		

**Data**	**Baseline data**	**COS Cycle data**[@]	**Follow-up data** [$]
Other laboratory measurements, as available (FSH, LH, estradiol, progesterone, TSH, prolactin as available)	**X**		
Results from imaging techniques, as available	**X**		
Results of most recent pelvic ultrasound as available	**X**		**X**
REKOVELLE® dosing regimen prescribed, number of days of treatment[&], dose changes		**X**	
Usage of algorithm		**X**	
Usage of dosing App		**X**	
Gonadotropin dosing regimen: initial dose prescribed		**X**	
Ovarian stimulation protocol used		**X**	
Stimulation day, Triggering of final follicular maturation		**X**	
Monitoring of the REKOVELLE® treatment (number and intents of each visit)		**X**	
Luteal support drugs used			**X**
Number of oocytes retrieved			**X**
Number of frozen oocytes/embryos/blastocysts			**X**
Number of embryos/blastocytes transferred			**X**
Quality of fresh/frozen embryos/blastocysts transferred			**X**
Pregnancy outcomes[+]			**X**
Pregnancy loss			**X**
Cycle cancelled and reason for cycle cancellation		**X**	**X**
ADRs serious and non-serious	**X**	**X**	**X**
Fatal events, related and non-related	**X**	**X**	**X**
OHSS (mild/moderate/severe)			**X**
Preventive measures for OHSS			**X**
Trial termination form			**X**
Medical history and concomitant treatment*	**X**	**X**	**X**
**Data collected by the Subjects**			
Subject Questionnaire[#]		**X**	

[@]Data to collect until oocyte retrieval or early withdrawal.
[$] Data to collect after oocyte retrieval, including transfer cycle or early withdrawal.
[&] REKOVELLE® treatment gathers all the treatment procedures related to the stimulation preparation and the stimulation period up to the triggering of the final follicular maturation.
[§] Written informed consent must be obtained prior to any Trial related data collection.

[+] Ultrasound results must be obtained if not performed at the participating site. Data about cycles using frozen embryos/blastocysts will be collected only for the first transfer cycle if they are performed within 3 months after the cycle stimulated with REKOVELLE® within the observation period.
[+] Pregnancy outcomes described with Positive signs of pregnancy (hCG test or urine pregnancy test) and clinical pregnancy (at least one gestational sac 5-6 weeks after transfer)
* Concomitant medication will be collected for all ADRs, both concomitant medication and medical history will be collected for all serious ADRs and fatal AEs.
[#] Subject questionnaire will be completed by the subjects at the clinic after cycle cancellation or before oocyte retrieval.
Appendix 2 SUBJECT QUESTIONNAIRE

6.2 Data collected in the e-CRF

6.2.1 Baseline Data

The following baseline data will be collected at the enrolment visit:

- Socio-demographic data: age, body weight, height, education level, socioeconomic status, professional categories, country of residence
- Reproductive history
  - Menstrual history
  - Infertility history: primary infertility, duration of infertility, primary reason for infertility, IVF or ICSI for the current parental project,
- Antral follicle count, as available
- Most recent AMH test result performed with ELECSYS AMH Plus from Roche or ACCESS AMH Advanced from Beckman Coulter or LUMIPULSE G AMH from Fujirebio Immunoassay, characteristics of the AMH test (e.g., type of laboratory, date of test)
- Baseline laboratory measurements: follicle stimulating hormone (FSH), Luteinizing Hormone (LH), estradiol, progesterone, thyroid-stimulating hormone (TSH), prolactin, as available
- Any results from imaging techniques and/or pelvic ultrasound as per local protocols, as available

6.2.2 COS Treatment Data

The following data about REKOVELLE® treatment will be collected:

- Individualized dosing regimen: daily dose of REKOVELLE® administered, number of days of treatment
- Use of the algorithm
- Use of the dosing App
- Ovarian stimulation protocol used
- Stimulation day and triggering method for final follicular maturation
- Monitoring of the REKOVELLE® treatment (number and intents of each visit)

6.2.3 Follow-up

No visits are mandated or prescheduled as part of the trial. Follow-up information will be collected by the physicians at one single visit scheduled as a part of routine care or may be collected via a telephone interview if a visit does not occur.

The following data will be collected for the 1st cycle with REKOVELLE®:

- Ultrasound results since last visit, as available
- Antral follicle count, as available
- Luteal phase support
- OHSS (including mild/moderate/severe)
- Preventive measures for OHSS:
  - Triggering of final follicular maturation with GnRH agonist and cancellation of fresh embryo/blastocyst transfer
- Oocytes retrieval: number of oocytes retrieved, number of metaphase II (MII) oocytes
- Number of frozen oocytes/embryos/blastocysts
- Freeze all strategy: reasons for freeze all strategy as alternative to fresh embryo transfer
- Fresh embryo/blastocyst transfer: day of embryo/blastocyst transfer, number and quality of embryos/blastocysts transferred
- Frozen embryo/blastocyst transfer (FET): endometrial preparation protocol, date of FET, number and quality of embryos/blastocysts transferred
- Pregnancy outcome: positive hCG, clinical pregnancy, vital pregnancy, ongoing pregnancy
- Pregnancy loss: biochemical pregnancy, spontaneous abortion, elective abortion, ectopic pregnancy, vanishing twin
- Cycle cancelled before oocyte pick-up, Transfer Cancellation, and reason for cycle cancellation:
  - Cancellation before oocyte pick-up (poor ovarian response, excessive ovarian response, any illness which prevents oocyte collection procedure, subject not taking hCG injection at the correct time, subject choice, other)
  - Transfer Cancellation after oocyte pick-up (no oocytes collected, no oocytes fertilised, abnormal fertilisation, abnormal embryo development, no embryo development, OHSS, subject choice, other)
- ADRs serious and non-serious, related / non-related fatal events

6.2.4 Trial Termination

The date of trial completion will be recorded in the trial termination form of the e-CRF.

In the event of trial withdrawal, the investigators will be asked to document the date and the reason for trial withdrawal in the trial termination form of the e-CRF.

6.2.5 Subject Questionnaire

The subject questionnaire will be completed by the subjects at the clinic after cycle cancellation or before oocyte retrieval.

7 SAFETY REPORTING

7.1 Definitions

7.1.1 Adverse Event

An AE is any untoward medical occurrence in a subject participating in a trial. It includes:

- Any unfavourable and unintended sign, symptom or disease temporally associated with the use of the REKOVELLE®, whether considered to be caused by the REKOVELLE®.
- Adverse events commonly observed and adverse events anticipated based on the pharmacological effect of the REKOVELLE®.
- Any laboratory abnormality, vital sign or finding from physical or gynaecological examination assessed as clinically significant by the investigator [note: findings from assessments and examinations done during screening are not adverse events, but are recorded as medical history].
- Accidental injuries, reasons for any change in medication (drug and/or dose), reasons for any medical, nursing or pharmacy consultation, or reasons for admission to hospital or surgical procedures.

7.1.2 Serious Adverse Events

Serious Adverse Events during the trial:

An event is defined as a serious adverse event if it:	Guidance
Results in **death**	Any event resulting in a fatal outcome must be fully documented and reported, including deaths occurring within four weeks after the treatment ends and irrespective of the causal relationship to REKOVELLE®. The death of a subject enrolled in a trial is per se not an event, but an outcome.
Is **life-threatening**	The term life-threatening refers to an adverse event in which the subject was at immediate risk of death at the time of the event. It does not refer to an event, which may have caused death if it were more severe.
Requires in-patient **hospitalisation** or prolongation of existing hospitalisation	The term hospitalisation means that the subject was admitted to hospital or that existing hospitalisation was extended as a result of an event.<br>Hospitalisation describes a period of at least 24 hours. Over-night stay for observation, stay at emergency room or treatment on an out-patient basis do not constitute a hospitalisation. However, medical judgement must always be exercised and when in doubt the case should be considered serious (i.e., if case fulfils the criterion for a medically important event).

An event is defined as a serious adverse event if it:	Guidance
	Hospitalisations for administrative or social purposes do not constitute a serious adverse event. Hospital admissions and/or surgical operations planned before trial inclusion are not considered adverse events, if the illness or disease existed before the subject was enrolled in the trial, provided that the condition did not deteriorate during the trial.
Results in persistent or significant **disability / incapacity**	Disability / incapacity means a substantial disruption of a person's ability to conduct normal life functions. In doubt, the decision should be left to medical judgement by the investigator.
Is a **congenital anomaly / birth defect**	Congenital anomaly / birth defect observed in any offspring of the subject conceived during treatment with REKOVELLE® (please refer to section 7.3)
Is an **important medical event**	Important medical events are events that may not be immediately life- threatening or result in death or hospitalization but may jeopardize the subject or may require intervention to prevent one of the other outcomes listed in the definition above. Examples of important medical events include adverse events that suggest a significant hazard, contraindication or precaution, occurrence of malignancy or development of drug dependency or drug abuse. Medical and scientific judgement should be exercised in deciding whether events qualify as medically important.<br>Important medical events include any suspected transmission of an infectious agent via a medicinal product. Any organism virus or infectious particle, pathogenic or non-pathogenic, is considered an infectious agent. A transmission of an infectious agent may be suspected from clinical symptoms or laboratory findings indicating an infection in a subject exposed to a medicinal product.

7.1.3 Causal Relationship to REKOVELLE®

The possibility of whether the REKOVELLE® caused the AE must be classified as follows:

- Reasonable possibility: There is evidence or argument to suggest a causal relationship between REKOVELLE® and the AE. The AE may occur as part of the pharmacological action of the REKOVELLE® or may be unpredictable in its occurrence.

Examples:

- o AEs that are uncommon but are known to be strongly associated with REKOVELLE® exposure.
- o AEs that are not commonly associated with REKOVELLE® exposure, but the event occurs in association with other factors strongly suggesting causation, such as a strong

temporal association with REKOVELLE® or the event recurs on rechallenge with REKOVELLE®.

- No reasonable possibility: There is no reasonable evidence or argument to suggest a causal relationship between REKOVELLE® and the adverse event.

Examples:

- Known consequences of the underlying disease or condition under investigation.
- AEs common in the trial population, which are also anticipated to occur with some frequency during the course of the trial, regardless of REKOVELLE® exposure.

An Adverse Drug Reaction (ADR), in contrast to an AE, is characterized by the fact that a causal relationship between REKOVELLE® and the AE is at least a reasonable possibility.

7.2 Adverse Event Resulting in Fatal Outcome

Any adverse event resulting in a fatal outcome must be fully documented and reported, including deaths occurring within four weeks after the treatment ends and irrespective of the causal relationship to REKOVELLE®. The death of a subject enrolled in the trial is per se not an event, but an outcome. See section 7.5 for reporting.

7.3 Congenital Abnormality

Any congenital abnormality discovered in the embryo or fetus during the trial must be fully documented, recorded and reported as a serious adverse event regardless of causality including any congenital anomaly which the Investigator may become aware of after end of trial. See section 7.5 for reporting.

7.4 Collection and Recording of Adverse Drug Reactions

REKOVELLE® (follitropin delta) is a recombinant human FSH with a well characterised safety profile documented in pivotal clinical trials. REKOVELLE® has been approved in EU via centralised procedure in December 2016 with a positive benefit-risk balance. Based on this, the scope of the safety data collection of adverse events in this non-interventional observational trial should be limited to collection of adverse drug reactions, both serious and non-serious [19].

This in addition to the report requirements stated in sections 7.2 and 7.3 in the protocol.

All ADRs to REKOVELLE®, whether serious or non-serious, are to be captured in the e-CRF and investigator must record the ADRs in the ADR Log provided in each subject's e-CRF with, as a minimum required, the following information:

- Adverse Drug Reaction
- Date of onset
- Intensity
- Action taken to REKOVELLE®
- Other action taken
- Date of outcome
- Outcome

- Seriousness criteria if applicable
- Short description of ADR (if not serious)

The data collected in the e-CRF for the ADRs is described in detail in the following sections.

Concomitant medication will be collected for all ADRs and Medical History will be collected for all serious ADRs.

Adverse Drug Reactions

Adverse Drug Reaction ADRs should be recorded as diagnoses, if available. If not, separate signs and symptoms should be recorded. One diagnosis / symptom should be entered per record.

If a subject suffers from the same ADR more than once and the subject fully recovers in between the events, the ADR should be recorded separately. If an ADR changes in intensity, a worst-case approach should be used when recording the ADR, i.e., the highest intensity and the longest duration of the event.

Note: A procedure is not an adverse event; the reason for conducting the procedure is. Hospitalisation is not an adverse event; the reason for hospitalisation is. Death is not an adverse event, but the cause of death is (an exception is sudden death of unknown cause, which is an adverse event).

Date of Onset

The date of onset is the date when the first sign(s) or symptom(s) were first noted. If the ADR is an abnormal clinically significant laboratory test or outcome of an examination, the onset date is the date the sample was taken, or the examination was performed.

Intensity

The intensity of ADRs must be classified using the following 3-point scale:

- Mild: Awareness of signs or symptoms, but no disruption of usual activity
- Moderate: Event sufficient to affect usual activity (disturbing)
- Severe: Inability to work or perform usual activities (unacceptable)

Action Taken to REKOVELLE®

The action taken to the REKOVELLE® in response to an ADR must be classified as one of the following:

- No change (medication schedule maintained or no action taken)
- Withdrawn
- Dose reduced
- Dose increased
- Not applicable

Other Action Taken

ADRs requiring therapy must be treated with recognised standards of medical care to protect the health and well-being of the subject. Appropriate resuscitation equipment and medicines must be available to ensure the best possible treatment of an emergency situation.

Date of Outcome

The date the subject recovered or recovered with sequelae.

Outcome

The outcome of an ADR must be classified as one of the following:

- Recovered (fully recovered or the condition has returned to the level observed at initiation of trial treatment)
- Recovered with sequelae (resulted in persistent or significant disability / incapacity)
- Recovering
- Not yet recovered
- Fatal

7.5 Collection, Recording and Reporting of Serious Adverse Drug Reactions

Serious Adverse Drug Reaction reporting by the Investigator

All serious ADRs (SADRs) must be reported immediately to Ferring Global Pharmacovigilance as soon as it becomes known to the investigator and not later than within 24 hours of their knowledge of the occurrence of a SADR.

The investigator is responsible for submitting the completed SADR Report Form with the fullest possible details within 3 calendar days of his/her knowledge of the SADR.

Serious Adverse Drug Reaction Report Form

The SADR Report Form is included in the e-CRF system and must be completed and submitted according to the instructions provided on the form. In case the e-CRF cannot be accessed and hence the SADR Report Form cannot be filled in within the e-CRF system, a paper SADR Report Form should be used and sent to Ferring Global Pharmacovigilance using the contact details below.

Global Pharmacovigilance, Ferring Pharmaceuticals A/S

mail to: safety.mailbox@ferring.com
Fax: (+45) 88380147

SADRs will be reported to the regulatory authorities according to local legislation. The investigator should comply with local legislation for safety reporting.

Additional Information

Additional information relevant to the SADR such as hospital records, results from investigations, e.g., laboratory parameters (that are not already uploaded in the e-CRF), invasive procedures, scans and x-rays, and autopsy results should be included in SADR narrative text.

The investigator will supply Ferring and the ethics committee with any additional requested information such as results of post-mortem examinations and hospital records.

7.6 Follow-up of Adverse Drug Reactions and Serious Adverse Drug Reactions

7.6.1 Follow-up of Adverse Drug Reactions

The investigator must follow-up on each ADR until it is resolved or until the medical condition of the subject is stable. All such relevant follow-up information must be reported to Ferring. If the event is a chronic condition, the investigator and Ferring may agree that further follow-up is not required.

7.6.2 Collection of Serious Adverse Drug Reactions with Onset after End of Trial

If an investigator becomes aware of a SAE after the end of the trial, and he/she assesses the SAE to have a reasonable possible causality to REKOVELLE® or another product where Ferring is Marketing Authorisation Holder, the case will have to be reported to Ferring, regardless how long after the end of the trial this takes place.

8 STATISTICAL METHODS

8.1 Sample Size

The sample size was planned to ensure that the trial obtains meaningful estimates for the description of REKOVELLE® patterns of use, ovarian stimulation and embryo development outcomes, and safety and subject satisfaction at the 1st cycle with REKOVELLE®.

This trial will be conducted in Nordic Countries (Sweden, Denmark, Norway) and Switzerland with the activation of 15 sites, known to follow-up women undergoing ART and to regularly prescribe REKOVELLE®. Each site will enrol maximum 15 subjects during the predefined inclusion period, until the total 200 subjects initiated with REKOVELLE® treatment enrolment target achieved in a competitive manner.

As the statistical analyses will be purely descriptive, the sample size is not based on any inferential statistical considerations.

8.2 Statistical Analysis

Full details of the statistical methods and the analyses will be provided in a Statistical Analysis Plan (SAP), which will be finalised prior to the database lock.

8.2.1 General Considerations

All data will be summarized using descriptive statistics:

- Continuous variables will be described (distribution) by their mean, standard deviation, median, quartiles 1 and 3, extreme values (minimum and maximum) and the number of missing data.
- Categorical variables will be described (counts) by the number and percentage of each response and the number of missing data.

8.2.2 Analysis Sets

Analysis Population: The Analysis Population will be defined as all subjects enrolled in the trial (signs ICF to participate in the study) who meet the inclusion/exclusion criteria and who receive at least one dose of REKOVELLE®.

Safety Population: The Safety Population will be defined as all subjects enrolled in the trial who received at least one dose of REKOVELLE®.

8.3 Data Analysis

8.3.1 Subject Disposition

A summary of all subjects enrolled into the trial, as well as subjects of the Analysis Population will be provided (including reasons for exclusion from the Analysis Population).

All trial withdrawals will be summarised. The reason for trial withdrawals will be also described.

8.3.2 Demographics and Other Baseline Characteristics

All baseline data will be presented using descriptive summary statistics on the Analysis Population.

Subject characteristics will be analysed in several subgroups, if sample size allows, including but not limited to: age in classes; AMH level; country.

8.3.3 Analysis of the Primary Endpoint

The REKOVELLE® treatment patterns will be described based on the Analysis Population:

Use of the algorithm-based individualised dosing regimen (daily dose of REKOVELLE® administered, number of days of treatment); use of the Algorithm dosing Application; use of GnRH protocol, type of GnRH protocol, triggering of follicle maturation; luteal phase support. The distribution of AMH test results will be described. Characteristics of the AMH test (test type, test date) will be described.

8.3.4 Analysis of the Secondary Endpoints

Assessment of the Ovarian stimulation and embryo development outcomes of REKOVELLE®: the following descriptive analyses will be performed based on the Analysis Population:

- Rate of ongoing pregnancy (≥1 intrauterine viable fetus 10-11 weeks after embryo transfer) in the fresh cycle will be calculated

- Rate of ongoing pregnancy (≥1 intrauterine viable fetus 10-11 weeks after embryo transfer) following the fresh cycle, including also the first frozen embryo transfer, will be calculated
- The distribution of the number of oocytes retrieved will be described
- The distribution of the number of oocytes/embryos/blastocysts frozen will be described
- The type of embryos/blastocysts transferred (fresh or frozen), the number and quality of embryos/blastocysts transferred (excellent, good, fair, other), and the day of embryo/blastocyst transfer will be described
- The Positive signs of pregnancy (hCG test or urine pregnancy test) and clinical pregnancy (at least one gestational sac 5-6 weeks after transfer) will be calculated
- The rate of pregnancy loss (biochemical pregnancy, spontaneous / elective abortion, ectopic pregnancy, vanishing twin) will be calculated
- The rate of cycles cancelled before or after oocyte pick-up will be calculated and the reason for cancellation will be described

Assessment of REKOVELLE® safety: the following descriptive analyses will be performed on the Safety Population:

- Description and rates of ADRs and SADRs to REKOVELLE®: all events will be coded by SOC (System Organ Class) and PT (Preferred Term) using MedDRA lowest level term (LLT)

The following descriptions will be made:

  - Number and proportion of subjects experiencing at least one ADR/SADR, and corresponding number of events
  - Number and proportion of subjects experiencing each ADR/SADR, and corresponding number of events
  - ADR/SADR severity and outcomes (fatal, temporary / permanent discontinuations)
  - All fatal AEs regardless of causality

- The rate of mild, moderate, severe early and late OHSS will be calculated. The time to OHSS occurrence and the OHSS duration will be described. The duration of hospitalisation, the medical and surgical interventions and the OHSS outcomes will be described
- The rate of preventive interventions for early OHSS will be calculated
- The drop-out rate will be calculated and the reasons for drop-out as reported by the women will be described

Assessment of REKOVELLE® subject satisfaction: the following descriptive analyses will be performed on the Analysis Population:

- The items of the subject satisfaction questionnaire will be described, subject experience and convenience score will be calculated with REKOVELLE®

Primary and secondary endpoints will be described in several subgroups including but not limited to: age in classes; AMH level; ovarian response; country.

9 DATA HANDLING

9.1 Data Abstraction

An electronic data capture (EDC) system provided by an independent third-party contract research organization (CRO) will be used. Data from the subject's medical record and collected during the routine visits will be entered into the e-CRF according to the schedule presented in the Table 1.

Subjects will be identified by use of the trial identification number assigned to them when they enrol in the trial.

The investigator and the trial centre's personnel will be trained on the EDC system by the sponsor and/or designee.

9.2 Electronic Case Report Forms and Data Capture System

Only authorized personnel will have access to the EDC system. Data will be entered into e-CRFs in accordance with instructions from the sponsor and/or designee. Each investigator will be responsible for ensuring that accurate data are entered into the e-CRF in a timely manner.

On-line logic checks will be built into the system, so that missing or illogical data are not submitted. In the event that inconsistent data persist, queries may be issued electronically to the trial centre and answered electronically by that trial centre's personnel. The identifying information (assigned username, date, and time) for both the originator of the query and the originator of the data change (if applicable), as well as the investigator's approval of all changes performed on the data, will be collected.

The investigator will be responsible for reviewing e-CRFs, resolving data queries generated by the sponsor and/or designee via the system, providing missing or corrected data, approving all changes performed on the subject data, and endorsing these data within the e-CRF. This approval method will include applying an electronic signature, a uniquely assigned username, and a password that together will represent a traditional handwritten signature.

The EDC system and the database will be hosted at the independent third-party CRO. After the trial database is declared clean, a final copy of the database will be stored at Ferring Clinical Database.

All data collected in the context of this trial will be stored and evaluated in accordance with regulatory requirements and applicable guidance for electronic records

9.3 Subject Questionnaire

Subject questionnaire will be completed on site after cycle cancellation or before the retrieval of oocytes, in the clinic. Only the data that are directly answered by the subject will be processed. The subject's name will not be used or entered.

9.4 Data Management Plan

A data management plan will be developed before the start of data collection and will describe all functions, processes, and specifications for data collection, cleaning, and validation.

9.5 Data Coding

Medications will be coded with the World Health Organization Drug Dictionary (WHO Drug), using the latest available version at the time of coding activities. ADRs will be coded with the most recent version of the Medical Dictionary for Regulatory Activities (MedDRA), using Lowest Level Terms.

10 MONITORING PROCEDURES

10.1 Training

Designated trial personnel will participate in a training program that will encourage consistency of process and procedures at the investigative site and ensure collection of high-quality data for this trial. All sites will be trained on the protocol, trial logistics and the e-CRF. Investigators will also be reminded of the processes and importance of reporting all ADRs.

10.2 Periodic Monitoring

The follow-up of this observational trial will be mainly conducted via remote monitoring. Sites will be managed on a regular basis with email and telephone contacts.

In addition, the sponsor or designee will monitor the trial at the site according to the Monitoring Plan. At the monitoring visits, the progress of the trial and any procedural or data issues will be discussed with the investigator and/or designee. Subject source documents should be available for review; the investigator will permit the sponsor, sponsor designee, the IRB/IEC, or regulatory authorities to inspect facilities and original records relevant to this trial.

In addition, clinical research associates (CRAs) will perform on site monitoring visits for data quality checking according to the monitoring plan. The aim of these monitoring visits is to check that information and Informed consent form has been signed by the subject, to verify key data from the e-CRF against the source data for a defined percentage of subjects, to confirm the correct reporting of adverse reactions according to the protocol, and to communicate with the investigator. At these on site monitoring visits, the progress of the trial and any procedural or data issues will be discussed with the investigator and/or designee.

10.3 Confidentiality of Subject Data

The investigator will ensure that the confidentiality of the subjects' data will be preserved. In the e-CRF or any other documents submitted to the sponsor or sponsor affiliate, the subjects will not be identified by their names, but by an identification system, which consists of an assigned number in the trial. Documents that are not for submission to the sponsor, e.g., the confidential subject identification code and the signed information and non-opposition form, will be maintained by the investigator in strict confidence.

In addition, no demographic data that could lead to the identification of an individual subject will be collected (e.g. the subject date of birth will neither be provided to the sponsor nor its designee, and only the subject's age will be collected).

However, data generated by this trial must be available for inspection upon request by representatives of national and local health authorities, sponsor monitors, representatives, and collaborators, and the IRB/EC for each trial site, as appropriate.

11 CHANGES IN CONDUCT OF THE TRIAL

11.1 Protocol Amendments

Any change to this Protocol will be documented in a Protocol Amendment, issued by the sponsor. Amendments will be submitted for consideration to the approving Independent Ethics Committees (IECs) and/or Regulatory Authority, as applicable. Changes to the protocol to eliminate immediate hazard(s) which could affect the safety of the subjects, may be implemented prior to ethics committee approval or favourable opinion.

11.2 Premature Termination of Trial Sites

The sponsor reserves the rights to terminate the participation of individual trial sites. Conditions that may warrant termination include but are not limited to, insufficient adherence to protocol requirements and failure to enrol subjects at an acceptable rate.

12 REPORTING AND PUBLICATION

12.1 Non-interventional Trial Report

The data and information collected during this trial will be reported in a Non-Interventional Trial Report prepared by the sponsor or its designee.

12.2 Confidentiality and Ownership of Trial Data

Any confidential information relating to the medicinal product or the trial, including any data and results from the trial will be the exclusive property of the sponsor. The investigator and any other persons involved in the trial will protect the confidentiality of this proprietary information belonging to Ferring.

12.3 Publication Policy

At the end of the trial, one or more manuscripts for joint publication may be prepared in collaboration between the Scientific Committee, the principal investigator or investigator(s) offered authorship and Ferring. Any publication of results must acknowledge all sites. Results from this multi-centre trial must be reported in entirety in a responsible and coherent manner and results from subsets should not be published in advance or without clear reference to the primary publication of the entire trial.

Authorship is granted based on the International Committee of Journal Medical Editors (ICMJE) criteria (see current official version: http://www.ICMJE.org). The total number of authors is based on the guideline from the relevant journal or congress. In the event of any disagreement in the content of a publication, both the investigator´s and Ferring's opinion will be fairly and sufficiently represented in the publication.

Any external CRO or laboratory involved in the conduct of this Non-Interventional Trial has no publication rights regarding this trial.

If the investigator wishes to independently publish/present any results from the trial, the draft manuscript/presentation must be submitted in writing to Ferring for comments prior to submission. Comments will be given within four weeks from receipt of the draft manuscript. This statement does not give Ferring any editorial rights over the content of a publication, other than to restrict the disclosure of Ferring's intellectual property and confidential information. If the matter considered for publication is deemed patentable by Ferring, Ferring will discuss how to make redactions and request that the scientific publication be delayed until after a filed patent application is published. Under such conditions the publication will be modified or delayed, to allow sufficient time for Ferring to seek patent protection of the invention.

12.4 Public Disclosure Policy

Ferring will register non-interventional studies in an appropriate registry, i.e., www.ClinicalTrials.gov, (a website maintained by the National Library of Medicine at the U.S. National Institutes of Health) and any local registries as required by local/national legislation and Ferring Internal SOPs.

13 ETHICAL AND REGULATORY ASPECTS

13.1 Institutional Review Board (IRB)/Independent Ethics Committee Approval (IEC)

The protocol and any amendments, any written materials given to the subjects and the information and non-opposition form should be reviewed and approved by the trial site's IRB/IEC before the trial is initiated in accordance with applicable national regulatory requirements. The investigator is then responsible for informing the IRB/IEC of the completion of the trial and should provide any required trial status and/or safety report(s) in accordance with applicable national regulatory requirements.

13.2 Regulatory Authority Authorisation/Approval/Notification

The regulatory permission to perform the trial will be obtained in accordance with applicable national regulatory requirements. All ethical and regulatory approvals must be available before the trial is initiated.

13.3 End of Trial

The total duration of the trial will be approximately 24 months (from Ethics Submission to Database Lock). Last subject out will correspond to the last ongoing pregnancy (10-11 weeks after the embryo/blastocyst transfer) among the women included during the enrolment period.

13.4 Subject Information and Consent

After the investigator has made the decision to treat the subject with REKOVELLE®, the investigators will inform the subject about the trial content. The subject must be given ample time to consider participation in the trial. In addition, the subject will be informed about her right of access, objection and correction of data recorded during this trial. Subject will also be informed about her right about oblivion and digital erasure. These rights may be exercised at any time through the investigator.

The investigator will explain that the subjects are completely free to refuse to enter in the trial or to withdraw from it at any time, without any consequences for their further care and without the need to justify their decision. In case the subject decides to early withdraw from the trial the data collected up to this point will be used (except if the subject expressly requires during the trial period to erase of all her data from the trial database).

The subject will receive a copy of the Subject Information and Consent Sheet.

Each subject will be informed that representatives from Ferring, IEC/IRB or regulatory authorities, in accordance with applicable regulatory requirements, may review her source records and data. Data protection will be handled in compliance with national/local regulations.

Individual subject data included in the trial database will be treated in compliance with all applicable laws and regulations regarding privacy protection.

This trial is intended to be conducted in non-vulnerable population. No vulnerable subject will be enrolled in the trial.

13.5 Data Collected on Investigators

Information related to participating investigators will be declared and the investigators will be informed – within the framework of their financial agreement – of their right to access, object to and correct this information.

14 LIABILITIES AND INSURANCE

14.1 Compliance Reference Documents

The Helsinki Declaration, Guidelines for Good Pharmacoepidemiology Practice (GPP; Initially Issued: 1996, Revision 1: August 2004, Revision 2. April 2007), and other national laws in the countries where the trial takes place shall constitute the main reference guidelines for ethical and regulatory conduct.

14.2 Liabilities and Insurance

Ferring is, as sponsor, responsible for ensuring appropriate general/product liability insurance and as required in accordance with applicable laws and regulations, country-specific liability insurance coverage for claims made by a trial subject for injury arising from the subject's participation in the trial.

15 ARCHIVING

15.1 Site File

The investigator is responsible for maintaining all the records, which enable the conduct of the trial at the site to be fully understood, in compliance with the GPP filing standard. The trial documentation including all the relevant correspondence should be kept by the investigator for at least 10 years, after the completion of the Non-Interventional Trial Report and if no further instructions are given by the sponsor.

The investigator is responsible for the completion and maintenance of the confidential subject identification code, which provides the sole link between named subject source records and anonymous e-CRF and subject data for the sponsor. The investigator must arrange for the retention of this Subject Identification Log and signed Subject information and non-opposition form for at least 10 years, after the completion of the Non-Interventional Trial Report and if no further instructions are given by the sponsor.

No trial site document may be destroyed without prior written agreement between the investigator and the sponsor. Should the investigator elect to assign the trial documents to another party, or move them to another location, the sponsor must be notified.

15.2 Trial Master File

The sponsor will archive the trial master file in accordance with GPP and applicable regulatory requirements.

16 REFERENCES

[1] F. Zegers-Hochschild et al., “International Committee for Monitoring Assisted Reproductive Technology (ICMART) and the World Health Organization (WHO) revised glossary of (ART) terminology, 2009,” Fertil. Steril., vol. 92, no. 5, pp. 1520–1524, Nov. 2009.
[2] “Diagnostic evaluation of the infertile male: a committee opinion,” Fertil. Steril., vol. 103, no. 3, pp. e18–e25, Mar. 2015.
[3] “Diagnostic evaluation of the infertile female: a committee opinion,” Fertil. Steril., vol. 98, no. 2, pp. 302–307, 2012.
[4] J. Boivin, L. Bunting, J. A. Collins, and K. G. Nygren, “International estimates of infertility prevalence and treatment-seeking: potential need and demand for infertility medical care,” Hum. Reprod., vol. 22, no. 6, pp. 1506–1512, Mar. 2007.
[5] M. N. Mascarenhas, S. R. Flaxman, T. Boerma, S. Vanderpoel, and G. A. Stevens, “National, Regional, and Global Trends in Infertility Prevalence Since 1990: A Systematic Analysis of 277 Health Surveys,” {PLoS} Med., vol. 9, no. 12, p. e1001356, 2012.
[6] J.-C. Arce et al., “Ovarian response to recombinant human follicle-stimulating hormone: a randomized, antimüllerian hormone–stratified, dose–response trial in women undergoing in vitro fertilization/intracytoplasmic sperm injection,” Fertil. Steril., vol. 102, no. 6, p. 1633–1640.e5, Dec. 2014.
[7] “Ovarian hyperstimulation syndrome,” Fertil. Steril., vol. 90, no. 5, pp. S188--S193, Nov. 2008.
[8] M. S. Kupka et al., “Assisted reproductive technology in Europe, 2010: results generated from European registers by ESHRE†.,” Hum. Reprod., vol. 29, no. 10, pp. 2099–113, Oct. 2014.

[9] R. Klemetti, T. Sevón, M. Gissler, and E. Hemminki, "Complications of IVF and ovulation induction," Hum. Reprod., vol. 20, no. 12, pp. 3293–3300, Dec. 2005.
[10] A. La Marca and S. K. Sunkara, "Individualization of controlled ovarian stimulation in IVF using ovarian reserve markers: from theory to practice," Hum. Reprod. Update, vol. 20, no. 1, pp. 124–140, Jan. 2014.
[11] F. J. Broekmans, J. Kwee, D. J. Hendriks, B. W. Mol, and C. B. Lambalk, "A systematic review of tests predicting ovarian reserve and IVF outcome," Hum. Reprod. Update, vol. 12, no. 6, pp. 685–718, Dec. 2006.
[12] A. La Marca et al., "Anti-Mullerian hormone (AMH) as a predictive marker in assisted reproductive technology (ART)," Hum. Reprod. Update, vol. 16, no. 2, pp. 113–130, Mar. 2010.
[13] Anckaert E, Smitz J, Schiettecatte J, Klein BM, Arce JC. The value of anti-Mullerian hormone measurement in the long GnRH agonist protocol: association with ovarian response and gonadotrophin-dose adjustments. Human reproduction (Oxford, England) 2012; 27(6): 1829-39.
[14] S. L. Broer, F. J. M. Broekmans, J. S. E. Laven, and B. C. J. M. Fauser, "Anti-Müllerian hormone: ovarian reserve testing and its potential clinical implications," Hum. Reprod. Update, vol. 20, no. 5, pp. 688–701, Sep. 2014.
[15] A. La Marca, G. Stabile, A. C. Artenisio, and A. Volpe, "Serum anti-Mullerian hormone throughout the human menstrual cycle," Hum. Reprod., vol. 21, no. 12, pp. 3103–3107, Dec. 2006.
[16] D. Gassner and R. Jung, "First fully automated immunoassay for anti-Müllerian hormone," Clin. Chem. Lab. Med., vol. 52, no. 8, Jan. 2014.
[17] A. Nyboe Andersen et al., "Individualized versus conventional ovarian stimulation for in vitro fertilization: a multicenter, randomized, controlled, assessor-blinded, phase 3 noninferiority trial," Fertil. Steril., vol. 107, no. 2, p. 387–396.e4, Feb. 2017.
[18] M. Brandes et al., "When and why do subfertile couples discontinue their fertility care? A longitudinal cohort trial in a secondary care subfertility population," Hum. Reprod., vol. 24, no. 12, pp. 3127–3135, Dec. 2009.
[19] "EMEA/873138/2011 Rev 2: Guideline on good pharmacovigilance practices (GVP). Module VI – Collection, management and submission of reports of suspected adverse reactions to medicinal products (Rev.2).," Aug. 2017.
[20] The general data protection regulation. http://www.consilium.europa.eu/policies/data-protection-reform/data-protection-regulation.
[21] Personalised modulation of ovarian response reduces OHSS risk: a randomised, controlled, assessor-blind trial comparing individualised follitropin delta versus conventional rFSH dosing in Japanese IVF/ICSI patients Ishihara O, Arce JC, for the Japanese Follitropin delta Phase 3 trial (STORK) group. Reproductive BioMedicine. Online DOI:https://doi.org/10.1016/j.rbmo.2021.01.023
[22] The interchangeability of two assays for the measurement of anti-Müllerian hormone when personalizing the dose of FSH in in vitro fertilization cycles. Antonio La Marca, Aarti Deenadayal Tolani & Martina Capuzzo. GYNECOLOGICAL ENDOCRINOLOGY https://doi.org/10.1080/09513590.2020.1810659
[23] Establishing the follitropin delta dose that provides a comparable ovarian response to 150 IU/day follitropin alfa Joan-Carles Arce1,* Per Larsson2, Juan Antonio García-Velasco
[24] A randomised controlled trial to clinically validate follitropin delta in its individualised dosing regimen for ovarian stimulation in Asian IVF/ICSI patients. Jie Qiao, Yunshan Zhang, Xiaoyan Liang, Tuong Ho, Hong-Yuan Huang, Sung-Hoon Kim, Marie Goethberg, Bernadette Mannaerts, and Joan-Carles Arce, on behalf of the Asian Follitropin Delta Phase 3 Trial (GRAPE) Group. Human Reproduction, Vol.00, No.0, pp. 1–16, 2021

[25] EMEA/873138/2011 Rev 2: Guideline on good pharmacovigilance practices (GVP). Module VI – Collection, management, and submission of reports of suspected adverse reactions to medicinal products (Rev.2). Date for coming into force of Revision 2 is 22 November 2017.

17 APPENDICES

Appendix 1 LIST OF CONTACTS

SPONSOR **Ferring**:

Represented by:

Dr. Brigitte Calles, M.D.– VP EU regional head of medical, pricing & reimbursement, public policies

P: +33149088529
M: +33622307761
E: brigitte.CALLES@ferring.com

TRIAL COORDINATOR Dr. Ani Azroyan, Ph.D. - European Medical Affairs Director

M: + 33647756684
E: ani.AZROYAN@ferring.com

Appendix 2 SUBJECT QUESTIONNAIRE

Subject questionnaire to assess overall experience and convenience among subjects using follitropin delta (REKOVELLE® pre-filled pen for injection during COS therapy for ART treatment)

REKOVELLE® is subject to safety monitoring. This will allow quick identification of new safety information. You can help by reporting any side effects you may get.

Reporting of side effects

If you get any side effects, talk to your doctor, pharmacist, or nurse. This includes any possible side effects not listed in the package leaflet.

You can also report side effects directly at
Global Pharmacovigilance, Ferring Pharmaceuticals A/S
mail to: safety.mailbox@ferring.com
Fax: (+45) 88380147
By reporting side effects, you can help provide more information on the safety of this medicine.

Date : Click or tap to enter a date.

1. How many days did you use the REKOVELLE® pen?

   ☐9 days

   ☐10 days

   ☐11 days

   ☐12 days

   ☐Other: (enter number of days) Click or tap here to enter text.

2. Who explained to you the handling of the REKOVELLE® pre-filled ready-to-use pen? (Tick more than one if applicable)

   ☐Physician

   ☐Nurse

   ☐Self-training (Instruction for use leaflet)

   ☐If other (text box) (e.g., company website, social media platforms etc.)

3. Who administered injections? (Tick more than one if applicable)

   ☐Myself

   ☐Partner

☐Both

☐Other: (text box)

		STRONGLY DISAGREE	DISAGREE	NEUTRAL/ NO OPINION	AGREE	STRONGLY AGREE
4.	The instructions material provided with the REKOVELLE® pen (Subject information (IFU)) was clear and helped me to understand how to use the pen correctly	☐	☐	☐	☐	☐
		VERY DIFFICULT	DIFFICULT	NEITHER EASY NOR DIFFICULT	EASY	VERY EASY
5.	Ease to prepare the device for injection (including needle attachment)	☐	☐	☐	☐	☐
6.	Ease to select the right dosage	☐	☐	☐	☐	☐
7.	Easy to correct the dosage if wrong dose is dialled	☐	☐	☐	☐	☐
		EXTREMELY INCONVENIENT	SOMEWHAT INCONVENIENT	NEUTRAL/ NO OPINION	SOMEWHAT CONVENIENT	EXTREMELY CONVENIENT
8.	Convenience of carrying and using while travelling	☐	☐	☐	☐	☐
9.	How would you rate the overall convenience of this device?	☐	☐	☐	☐	☐
		EXTREMELY UNSATISFIED	SOMEWHAT UNSATISFIED	NEUTRAL/NO OPINION	SOMEWHAT SATISFIED	EXTREMELY SATISFIED
10.	Overall satisfaction	☐	☐	☐	☐	☐
