# Supplementary material for: Effectiveness and safety of follitropin delta in routine clinical practice in the Nordics and Switzerland (the NORSOS study): a prospective non-interventional study
Source: Front Endocrinol (Lausanne). 2025 Sep 5;16:1613680. doi: 10.3389/fendo.2025.1613680 (PMC12446022; doi:10.3389/fendo.2025.1613680)
Supplement: Supplementary file 2 [file DataSheet2.pdf]

ANNEX I

SUMMARY OF PRODUCT CHARACTERISTICS

1. NAME OF THE MEDICINAL PRODUCT

REKOVELLE 12 micrograms/0.36 mL solution for injection in a pre-filled pen
REKOVELLE 36 micrograms/1.08 mL solution for injection in a pre-filled pen
REKOVELLE 72 micrograms/2.16 mL solution for injection in a pre-filled pen

2. QUALITATIVE AND QUANTITATIVE COMPOSITION

REKOVELLE 12 micrograms/0.36 mL solution for injection
One pre-filled multidose pen delivers 12 micrograms follitropin delta* in 0.36 mL solution.

REKOVELLE 36 micrograms/1.08 mL solution for injection
One pre-filled multidose pen delivers 36 micrograms follitropin delta* in 1.08 mL solution.

REKOVELLE 72 micrograms/2.16 mL solution for injection
One pre-filled multidose pen delivers 72 micrograms follitropin delta* in 2.16 mL solution.

One mL of solution contains 33.3 micrograms of follitropin delta*
*recombinant human follicle-stimulating hormone (FSH) produced in a human cell line (PER.C6) by recombinant DNA technology.

For the full list of excipients, see section 6.1.

3. PHARMACEUTICAL FORM

Solution for injection in a pre-filled pen (injection).

Clear and colourless solution with a pH of 6.0-7.0.

4. CLINICAL PARTICULARS

4.1 Therapeutic indications

Controlled ovarian stimulation for the development of multiple follicles in women undergoing assisted reproductive technologies (ART) such as an *in vitro f*ertilisation (IVF) or intracytoplasmic sperm injection (ICSI) cycle.

4.2 Posology and method of administration

Treatment should be initiated under the supervision of a physician experienced in the treatment of fertility problems.

Posology
The posology of REKOVELLE is individualised for each patient and aims to obtain an ovarian response which is associated with a favourable safety/efficacy profile, i.e. aims to achieve an adequate number of oocytes retrieved and reduce the interventions to prevent ovarian hyperstimulation syndrome (OHSS). REKOVELLE is dosed in micrograms (see section 5.1). The dosing regimen is specific for REKOVELLE and the microgram dose cannot be applied to other gonadotropins.

For the first treatment cycle, the individual daily dose will be determined on the basis of the woman's serum anti-Müllerian hormone (AMH) concentration and her body weight. The dose should be based on a recent determination of AMH (i.e. within the last 12 months) measured by the following diagnostic tests: ELECSYS AMH Plus immunoassay from Roche (i.e. assay used in clinical development trials), or alternatively the ACCESS AMH Advanced from Beckman Coulter or

LUMIPULSE G AMH from Fujirebio (see section 4.4). The individual daily dose is to be maintained throughout the stimulation period. For women with AMH <15 pmol/L the daily dose is 12 micrograms, irrespective of body weight. For women with AMH ≥15 pmol/L the daily dose decreases from 0.19 to 0.10 micrograms/kg by increasing AMH concentration (Table 1). The dose is to be rounded off to the nearest 0.33 micrograms to match the dosing scale on the injection pen. The maximum daily dose for the first treatment cycle is 12 micrograms.
For calculation of the REKOVELLE dose, the body weight is to be measured without shoes and overcoat just prior to start of stimulation.

Table 1 Dosing regimen

**AMH (pmol/L)**	<15	15-16	17	18	19-20	21-22	23-24	25-27	28-32	33-39	≥40
**Fixed daily dose of REKOVELLE**	12	0.19	0.18	0.17	0.16	0.15	0.14	0.13	0.12	0.11	0.10
	mcg	mcg/kg									

The AMH concentration is to be expressed in pmol/L and is to be rounded off to the nearest integer. If the AMH concentration is in ng/mL, the concentration should be converted to pmol/L by multiplying with 7.14 (ng/mL x 7.14 = pmol/L) before use.
mcg: micrograms

Potential high responders (patients with AMH >35 pmol/L) have not been studied in a protocol using down-regulation with GnRH agonist.

Time of initiating treatment with REKOVELLE depends on the type of protocol.

- in a protocol using a gonadotropin-releasing hormone (GnRH) antagonist, the treatment with REKOVELLE should be initiated on day 2 or 3 after start of menstrual bleeding;
- in a protocol using down-regulation with a GnRH agonist, the treatment with REKOVELLE should be initiated approximately 2 weeks after the start of agonist treatment.

Treatment should continue until adequate follicular development (≥3 follicles ≥17 mm) has been achieved, which on average is by the ninth or tenth day of treatment (range 5 to 20 days). With pituitary desensitisation caused by a GnRH agonist, a longer duration of stimulation and therefore a higher total dose of REKOVELLE may be necessary to achieve adequate follicular response. A single injection of 250 micrograms recombinant human chorionic gonadotropin (hCG) or 5,000 IU hCG is administered to induce final follicular maturation. In patients with excessive follicular development (of ≥25 follicles ≥12 mm), treatment with REKOVELLE should be stopped and triggering of final follicular maturation with hCG should not be performed.

For subsequent treatment cycles, the daily dose of REKOVELLE should be maintained or modified according to the patient’s ovarian response in the previous cycle. If the patient had adequate ovarian response in the previous cycle without developing OHSS, the same daily dose should be used. In case of ovarian hypo-response in the previous cycle, the daily dose in the subsequent cycle should be increased by 25% or 50%, according to the extent of response observed. In case of ovarian hyper-response in the previous cycle, the daily dose in the subsequent cycle should be decreased by 20% or 33%, according to the extent of response observed. In patients who developed OHSS or were at risk of OHSS in a previous cycle, the daily dose for the subsequent cycle is 33% lower than the dose used in the cycle where OHSS or risk of OHSS occurred. The maximum daily dose is 24 micrograms.

Elderly
There is no relevant use of REKOVELLE in the elderly population.

Patients with renal and hepatic impairment
Safety, efficacy and pharmacokinetics of REKOVELLE in patients with renal or hepatic impairment have not been specifically studied in clinical trials. Although limited, data did not indicate a need for a different dosing regimen of REKOVELLE in this patient population (see section 4.4).

Polycystic ovarian syndrome patients with anovulatory disorders
Anovulatory patients with polycystic ovarian syndrome have not been studied. Ovulatory patients with polycystic ovaries have been included in clinical trials (see section 5.1).

Paediatric population
There is no relevant use of REKOVELLE in the paediatric population.

Method of administration
REKOVELLE is intended for subcutaneous use, preferably in the abdominal wall. The first injection should be performed under direct medical supervision. Patients must be educated on how to use the REKOVELLE injection pen and to perform injections. Self-administration should only be performed by patients who are well motivated, adequately trained and have access to expert advice.

For instructions on the administration with the pre-filled pen, see the ”Instructions for Use”.

4.3 Contraindications

- Hypersensitivity to the active substance or to any of the excipients listed in section 6.1
- tumours of the hypothalamus or pituitary gland
- ovarian enlargement or ovarian cyst not due to polycystic ovarian syndrome
- gynaecological haemorrhages of unknown aetiology (see section 4.4)
- ovarian, uterine or mammary carcinoma (see section 4.4)

In the following situations, treatment outcome is unlikely to be favourable, and therefore REKOVELLE should not be administered:
- primary ovarian failure
- malformations of sexual organs incompatible with pregnancy
- fibroid tumours of the uterus incompatible with pregnancy

4.4 Special warnings and precautions for use

Traceability
In order to improve the traceability of biological medicinal products, the name and the batch number of the administered product should be clearly recorded.

REKOVELLE contains a potent gonadotropic substance capable of causing mild to severe adverse reactions, and should only be used by physicians who are thoroughly familiar with infertility problems and their management.

Gonadotropin therapy requires time commitment by physicians and supportive healthcare professionals, as well as the availability of appropriate monitoring facilities. Safe and effective use of REKOVELLE calls for monitoring of ovarian response with ultrasound alone, or in combination with measurement of serum estradiol levels, on a regular basis. The dose of REKOVELLE is individualised for each patient to obtain an ovarian response with favourable safety/efficacy profile. There may be a degree of interpatient variability in response to FSH administration, with poor response to FSH in some patients and exaggerated response in others.

Before starting treatment, the couple’s infertility should be assessed as appropriate and putative contraindications for pregnancy evaluated. In particular, patients should be evaluated for hypothyroidism and hyperprolactinemia, and the appropriate specific treatment should be given.

Use of results obtained with other assays than the ELECSYS AMH Plus immunoassay from Roche, the ACCESS AMH Advanced from Beckman Coulter and LUMIPULSE G AMH from Fujirebio for REKOVELLE dose determination is not recommended, as there currently is no standardisation of available AMH assays.

Patients undergoing stimulation of follicular growth may experience ovarian enlargement and may be at risk of developing OHSS. Adherence to the REKOVELLE dose and regimen of administration and careful monitoring of therapy will minimise the incidence of such events.

Ovarian Hyperstimulation Syndrome (OHSS)
A certain degree of ovarian enlargement is an expected effect of controlled ovarian stimulation. It is more commonly seen in patients with polycystic ovarian syndrome and usually regresses without treatment. In distinction to uncomplicated ovarian enlargement, OHSS is a condition that can manifest itself with increasing degrees of severity. It comprises marked ovarian enlargement, high serum sex steroids, and an increase in vascular permeability which can result in an accumulation of fluid in the peritoneal, pleural and, rarely, in the pericardial cavities.

It is important to stress the value of careful and frequent monitoring of follicular development in order to reduce the risk of OHSS. The following symptoms may be observed in severe cases of OHSS: abdominal pain, discomfort and distension, severe ovarian enlargement, weight gain, dyspnoea, oliguria and gastrointestinal symptoms including nausea, vomiting and diarrhoea. Clinical evaluation may reveal hypovolaemia, haemoconcentration, electrolyte imbalances, ascites, haemoperitoneum, pleural effusions, hydrothorax, or acute pulmonary distress. Very rarely, severe OHSS may be complicated by ovarian torsion or thromboembolic events such as pulmonary embolism, ischaemic stroke or myocardial infarction.

Excessive ovarian response to gonadotropin treatment seldom gives rise to OHSS unless hCG is administered to trigger final follicular maturation. Furthermore, the syndrome may be more severe and more protracted if pregnancy occurs. Therefore, in cases of ovarian hyperstimulation it is prudent to withhold hCG and advise the patient to refrain from coitus or to use barrier contraceptive methods for at least 4 days. OHSS may progress rapidly (within 24 hours) to several days to become a serious medical event. Early OHSS can occur within 9 days after triggering of final follicular maturation. Late OHSS can develop as a consequence of the hormonal changes during pregnancy 10 or more days after triggering of final follicular maturation. Because of the risk of developing OHSS patients should be followed for at least two weeks after hCG administration.

Thromboembolic events
Women with recent or ongoing thromboembolic disease or women with generally recognised risk factors for thromboembolic events, such as personal or family history, severe obesity (body mass index >30 kg/m^2) or thrombophilia may have an increased risk of venous or arterial thromboembolic events, during or following treatment with gonadotropins. Treatment with gonadotropins may further increase the risk for aggravation or occurrence of such events. In these women, the benefits of gonadotropin administration need to be weighed against the risks. It should be noted however that pregnancy itself as well as OHSS also carry an increased risk of thromboembolic events.

Ovarian torsion
Occurrence of ovarian torsion has been reported for ART cycles. It may be associated with other risk factors such as OHSS, pregnancy, previous abdominal surgery, past history of ovarian torsion, previous or current ovarian cyst and polycystic ovaries. Damage to the ovary due to reduced blood supply can be limited by early diagnosis and immediate detorsion.

Multiple pregnancy
Multiple pregnancy carries an increased risk of adverse maternal and perinatal outcomes. In patients undergoing ART procedures the risk of multiple pregnancy is related mainly to the number of embryos replaced, their quality and the patient age, although twin pregnancy can in rare occasions develop from single embryo transfers. The patients should be advised of the potential risk of multiple births before starting treatment.

Pregnancy loss
The incidence of pregnancy loss by miscarriage or abortion is higher in patients undergoing controlled ovarian stimulation for ART than following natural conception.

Ectopic pregnancy
Women with a history of tubal disease are at risk of ectopic pregnancy, whether the pregnancy is obtained by spontaneous conception or with fertility treatments. The prevalence of ectopic pregnancy after ART has been reported to be higher than in the general population.

Reproductive system neoplasms
There have been reports of ovarian and other reproductive system neoplasms, both benign and malignant, in women who have undergone multiple treatment regimens for infertility treatment. It is not established whether or not treatment with gonadotropins increases the risk of these tumours in infertile women.

Congenital malformation
The prevalence of congenital malformations after ART may be slightly higher than after spontaneous conceptions. This is thought to be due to differences in parental characteristics (e.g. maternal age, sperm characteristics) and multiple pregnancy.

Other medical conditions
Medical conditions that contraindicate pregnancy should also be evaluated before starting treatment with REKOVELLE.

Renal and hepatic impairment
REKOVELLE has not been studied in patients with moderate/severe renal or hepatic impairment.

Sodium content
REKOVELLE contains less than 1 mmol sodium (23 mg) per dose, that is to say essentially “sodium-free”.

4.5 Interaction with other medicinal products and other forms of interaction

No interaction studies have been performed with REKOVELLE. Clinically significant interactions with other medicinal products have neither been reported during REKOVELLE therapy, nor are expected.

4.6 Fertility, pregnancy and lactation

Pregnancy
REKOVELLE is not indicated during pregnancy. No teratogenic risk has been reported, following controlled ovarian stimulation, in clinical use with gonadotropins. There are no data from the inadvertent exposure to REKOVELLE in pregnant women. Studies in animals have shown reproductive toxicity with REKOVELLE doses above the recommended maximal dose in humans (section 5.3).

Breast-feeding
REKOVELLE is not indicated during breastfeeding.

Fertility
REKOVELLE is indicated for use in infertility (see section 4.1).

4.7 Effects on ability to drive and use machines

REKOVELLE has no or negligible influence on the ability to drive and use machines.

4.8 Undesirable effects

Summary of safety profile
The most frequently reported adverse reactions during treatment with REKOVELLE are OHSS, headache, pelvic pain, nausea and fatigue. The frequency of these adverse reactions might decrease with repeated treatment cycles, as this has been observed in clinical trials.

Tabulated list of adverse reactions
The table below (Table 2) displays the adverse reactions experienced in clinical trials by patients treated with REKOVELLE using the algorithm-based dosing regimen. Within each frequency grouping, adverse reactions are presented in order of decreasing seriousness.

Table 2 Adverse reactions in pivotal clinical trials

System Organ Class	Common (≥1/100 to <1/10)	Uncommon (≥1/1,000 to <1/100)
Psychiatric disorders		Mood swings
Nervous system disorders	Headache	Somnolence<br>Dizziness
Gastrointestinal disorders	Nausea	Diarrhoea<br>Vomiting<br>Constipation<br>Abdominal discomfort[a]
Reproductive system and breast disorders	OHSS<br>Pelvic pain[b]	Vaginal haemorrhage<br>Breast discomfort[c]
General disorders and administration site conditions	Fatigue	

a Abdominal discomfort includes abdominal pain/distention.
b Pelvic pain includes pelvic discomfort and adnexa uteri pain.
c Breast discomfort includes breast pain, breast swelling, breast tenderness and/or nipple pain.

Description of selected adverse reactions
OHSS is an intrinsic risk of the ovarian stimulation. Known gastrointestinal symptoms associated with OHSS include abdominal pain, discomfort, and distension, nausea, vomiting and diarrhoea. Ovarian torsion and thromboembolic events are known to be rare complications of ovarian stimulation treatment (see section 4.4).
Immunogenicity in terms of development of anti-FSH antibodies is a potential risk of gonadotropin therapy (see section 5.1).

Reporting of suspected adverse reactions
Reporting suspected adverse reactions after authorisation of the medicinal product is important. It allows continued monitoring of the benefit/risk balance of the medicinal product. Healthcare professionals are asked to report any suspected adverse reactions via the national reporting system listed in Appendix V.

4.9 Overdose

The effect of an overdose is unknown, nevertheless, there is a risk that OHSS may occur (see section 4.4).

5. PHARMACOLOGICAL PROPERTIES

5.1 Pharmacodynamic properties

Pharmacotherapeutic group: Sex hormones and modulators of the genital system, gonadotropins, ATC code: G03GA10

Mechanism of action
The most important effect resulting from parenteral administration of FSH is the development of multiple mature follicles.

Follitropin delta is a recombinant human FSH. The amino acid sequences of the two FSH subunits in follitropin delta are identical to the endogenous human FSH sequences. Because follitropin delta is produced in the human cell line PER.C6, the glycosylation profile is different from follitropin alfa and follitropin beta.

Pharmacodynamic effects
Following daily administration of equal IU doses of REKOVELLE and follitropin alfa as determined in the rat *in vivo* bioassay (Steelman-Pohley assay), higher ovarian response (i.e. estradiol, inhibin B and follicular volume) was observed in patients after administration of REKOVELLE compared to follitropin alfa. As the rat bioassay might not fully reflect the potency of the FSH in REKOVELLE in humans, REKOVELLE is dosed in micrograms and not in IU. The clinical trial data suggest that a daily dose of 10.0 [95% CI 9.2; 10.8] micrograms REKOVELLE provides, for the majority of patients, an ovarian response close to that obtained with 150 IU/day follitropin alfa.

The number of oocytes retrieved increases with the dose of REKOVELLE and serum AMH concentration. Conversely, increasing body weight leads to a decrease in the number of oocytes retrieved (only clinically relevant for REKOVELLE doses below 12 micrograms). The resulting REKOVELLE dosing regimen is in section 4.2.

Clinical efficacy and safety
The ESTHER-1 trial was a randomised, assessor-blinded, controlled trial in 1,326 IVF/ICSI patients. The trial compared the individualised dosing regimen of REKOVELLE where the daily dose is established for each patient and fixed throughout stimulation with no adjustments (see section 4.2) to follitropin alfa filled-by-mass at a starting dose of 11 micrograms (150 IU) for the first five days followed by dose adjustments from day 6 of stimulation based on follicular development in a GnRH antagonist protocol. The patients were up to 40 years of age and had regular menstrual cycles presumed to be ovulatory. Single blastocyst transfer on day 5 was compulsory with the exception of patients 38-40 years in whom double blastocyst transfer was performed if no good-quality blastocysts were available. The two co-primary endpoints were ongoing pregnancy rate and ongoing implantation rate in the fresh cycle, defined as at least one intrauterine viable fetus 10-11 weeks after transfer and number of intrauterine viable fetuses 10-11 weeks after transfer divided by number of blastocysts transferred, respectively.

The trial demonstrated that REKOVELLE was at least as effective as follitropin alfa in terms of ongoing pregnancy rate and ongoing implantation rate, as shown in Table 3.

Table 3 Ongoing pregnancy rate and ongoing implantation rate in ESTHER-1 trial

	REKOVELLE in an individualised dosing regimen (N=665)	Follitropin alfa (N=661)	Difference [95% CI]
Ongoing pregnancy rate	30.7%	31.6%	-0.9% [-5.9%; 4.1%]
Ongoing implantation rate	35.2%	35.8%	-0.6% [-6.1%; 4.8%]

Population: all randomised and exposed

The impact of the AMH-based dosing regimen of REKOVELLE was also assessed in secondary endpoints, such as ovarian response and OHSS risk management.

In the overall trial population, the mean number of oocytes retrieved was 10.0 ± 5.6 with REKOVELLE (N=636) in the individualised dosing regimen and 10.4 ± 6.5 with follitropin alfa (N=643) at a starting dose of 150 IU followed by dose adjustments.

Among patients with AMH ≥15 pmol/L, the ovarian response with REKOVELLE (N=355) and follitropin alfa (N=353), respectively, was as follows: mean number of oocytes retrieved 11.6 ± 5.9 and 13.3 ± 6.9, and proportion of patients with ≥20 oocytes 10.1% (36/355) and 15.6% (55/353).

In ovulatory patients with polycystic ovaries undergoing a GnRH antagonist cycle, the incidence of early moderate/severe OHSS and/or preventive interventions for early OHSS was 7.7% with REKOVELLE and 26.7% with follitropin alfa.

In a controlled trial evaluating the ovarian response with individualised REKOVELLE dosing in patients with AMH ≤35 pmol/L, the mean number of oocytes was 11.1 ± 5.9 in a GnRH agonist cycle (N=202) compared to 9.6 ± 5.5 in a GnRH antagonist cycle (N=204), and the mean duration of stimulation with REKOVELLE was 10.4 ± 1.9 days in a GnRH agonist cycle compared to 8.8 ± 1.8 days in a GnRH antagonist cycle.

Safety – immunogenicity
Anti-FSH antibodies were measured pre-dosing and post-dosing in patients undergoing up to three repeated treatment cycles with REKOVELLE (665 patients in cycle 1 in the ESTHER-1 trial as well as 252 patients in cycle 2 and 95 patients in cycle 3 in the ESTHER-2 trial). The incidence of anti-FSH antibodies after treatment with REKOVELLE was 1.1% in cycle 1, 0.8% in cycle 2 and 1.1% in cycle 3. These rates were similar to the incidence of pre-existing anti-FSH antibodies before exposure to REKOVELLE in cycle 1 which was 1.4%, and comparable to the incidences of anti-FSH antibodies after treatment with follitropin alfa. In all patients with anti-FSH antibodies, titres were undetectable or very low and without neutralising capacity. Repeated treatment with REKOVELLE of patients with pre-existing or treatment-induced anti-FSH antibodies did not increase the antibody titre, was not associated with decreased ovarian response, and did not induce immune-related adverse events.

Clinical trial experience with REKOVELLE in the long GnRH agonist protocol is limited.

5.2 Pharmacokinetic properties

The pharmacokinetic profile of follitropin delta has been investigated in healthy female subjects and in IVF/ICSI patients undergoing COS. Following repeated daily subcutaneous administrations, REKOVELLE reaches steady-state within 6 to 7 days with a threefold higher concentration compared with the concentration after the first dose. Circulating levels of follitropin delta are inversely related to the body weight, which supports individualised dosing based on body weight. Follitropin delta leads to greater exposure than follitropin alfa.

Absorption
After daily subcutaneous administration of REKOVELLE, the time to maximum serum concentration is 10 hours. The absolute bioavailability is about 64%.

Distribution
The apparent volume of distribution is about 25 L after subcutaneous administration and the volume of distribution at steady state is 9 L after intravenous administration. Within the therapeutic dose range, exposure to follitropin delta increases proportionally with the dose.

Elimination

Following subcutaneous administration, the apparent clearance of follitropin delta is 0.6 L/h and the clearance after intravenous is 0.3 L/h. The terminal elimination half-life after single subcutaneous administration is 40 hours and after multiple subcutaneous administration is 28 hours. The apparent clearance for follitropin delta is low, i.e. 0.6 L/h after multiple subcutaneous administration, leading to high exposure. Follitropin delta is expected to be eliminated similarly to other follitropins, i.e. mainly by the kidneys. The fraction of follitropin delta excreted unchanged in the urine was estimated to 9%.

5.3 Preclinical safety data

Non-clinical data reveal no special hazard for humans based on conventional studies of safety pharmacology, repeated dose toxicity and local tolerance. The overdose of follitropin delta resulted in pharmacological or exaggerated pharmacological actions. Follitropin delta had a negative effect on fertility and early embryonic development in rats when administered in doses ≥0.8 micrograms/kg/day which is above the recommended maximal dose in humans. The relevance of these findings for the clinical use of REKOVELLE is limited.

6. PHARMACEUTICAL PARTICULARS

6.1 List of excipients

Phenol
Polysorbate 20
L-methionine
Sodium sulphate decahydrate
Disodium phosphate dodecahydrate
Phosphoric acid, concentrated (for pH-adjustment)
Sodium hydroxide (for pH-adjustment)
Water for injections

6.2 Incompatibilities

Not applicable.

6.3 Shelf life

3 years

In use: 28 days when stored at or below 25 °C.

6.4 Special precautions for storage

Store in a refrigerator (2 °C – 8 °C).
Do not freeze.
Store in the original package in order to protect from light.

REKOVELLE may be removed from the refrigerator, without being refrigerated again, and stored at or below 25 °C for up to 3 months including the period after first use. It must be discarded afterwards.

For storage conditions after first use of the medicinal product, see section 6.3.

6.5 Nature and contents of container

<u>REKOVELLE 12 micrograms/0.36 mL solution for injection</u>
3 mL multidose cartridge (Type I glass) with a plunger (halobutyl rubber) and a crimp cap (aluminium) with an inlay (rubber). Each cartridge contains 0.36 mL of solution.
Pack size of 1 pre-filled pen and 3 injection needles (stainless steel).

REKOVELLE 36 micrograms/1.08 mL solution for injection
3 mL multidose cartridge (Type I glass) with a plunger (halobutyl rubber) and a crimp cap (aluminium) with an inlay (rubber). Each cartridge contains 1.08 mL of solution.
Pack size of 1 pre-filled pen and 9 injection needles (stainless steel).

REKOVELLE 72 micrograms/2.16 mL solution for injection
3 mL multidose cartridge (Type I glass) with a plunger (halobutyl rubber) and a crimp cap (aluminium) with an inlay (rubber). Each cartridge contains 2.16 mL of solution.
Pack size of 1 pre-filled pen and 15 injection needles (stainless steel).

6.6 Special precautions for disposal and other handling

The solution should not be administered if it contains particles or is not clear.

The instructions for use of the pen must be followed. Discard used needles immediately after injection.

Any unused medicinal product or waste material should be disposed of in accordance with local requirements.

7. MARKETING AUTHORISATION HOLDER

Ferring Pharmaceuticals A/S
Amager Strandvej 405
2770 Kastrup
Denmark

8. MARKETING AUTHORISATION NUMBER(S)

EU/1/16/1150/004
EU/1/16/1150/005
EU/1/16/1150/006

9. DATE OF FIRST AUTHORISATION/RENEWAL OF THE AUTHORISATION

Date of first authorisation: 12 December 2016
Date of latest renewal: 16 July 2021

10. DATE OF REVISION OF THE TEXT

Detailed information on this medicinal product is available on the website of the European Medicines Agency http://www.ema.europa.eu.

ANNEX II

A. MANUFACTURER OF THE BIOLOGICAL ACTIVE SUBSTANCE AND MANUFACTURER RESPONSIBLE FOR BATCH RELEASE

B. CONDITIONS OR RESTRICTIONS REGARDING SUPPLY AND USE

C. OTHER CONDITIONS AND REQUIREMENTS OF THE MARKETING AUTHORISATION

D. CONDITIONS OR RESTRICTIONS WITH REGARD TO THE SAFE AND EFFECTIVE USE OF THE MEDICINAL PRODUCT

A. MANUFACTURER OF THE BIOLOGICAL ACTIVE SUBSTANCE AND MANUFACTURER RESPONSIBLE FOR BATCH RELEASE

Name and address of the manufacturer of the biological active substance

Bio-Technology General (Israel) Ltd.
Be'er Tuvia Industrial Zone
POB 571
Kiryat Malachi 8310402
Israel

Name and address of the manufacturer responsible for batch release

Ferring GmbH
Wittland 11
D-24109 Kiel
Germany

B. CONDITIONS OR RESTRICTIONS REGARDING SUPPLY AND USE

Medicinal product subject to restricted medical prescription (see Annex I: Summary of Product Characteristics, section 4.2)

C. OTHER CONDITIONS AND REQUIREMENTS OF THE MARKETING AUTHORISATION

- **Periodic safety update reports (PSURs)**

The requirements for submission of PSURs for this medicinal product are set out in the list of Union reference dates (EURD list) provided for under Article 107c(7) of Directive 2001/83/EC and any subsequent updates published on the European medicines web-portal.

The marketing authorisation holder (MAH) shall submit the first PSUR for this product within 6 months following authorisation.

D. CONDITIONS OR RESTRICTIONS WITH REGARD TO THE SAFE AND EFFECTIVE USE OF THE MEDICINAL PRODUCT

- **Risk management plan (RMP)**

The marketing authorisation holder (MAH) shall perform the required pharmacovigilance activities and interventions detailed in the agreed RMP presented in Module 1.8.2 of the marketing authorisation and any agreed subsequent updates of the RMP.

An updated RMP should be submitted:

- At the request of the European Medicines Agency;
- Whenever the risk management system is modified, especially as the result of new information being received that may lead to a significant change to the benefit/risk profile or as the result of an important (pharmacovigilance or risk minimisation) milestone being reached.

ANNEX III

LABELLING AND PACKAGE LEAFLET

A. LABELLING

PARTICULARS TO APPEAR ON THE OUTER PACKAGING

OUTER CARTON

1. NAME OF THE MEDICINAL PRODUCT

REKOVELLE 12 micrograms/0.36 mL solution for injection in a pre-filled pen
follitropin delta

2. STATEMENT OF ACTIVE SUBSTANCE(S)

Each pre-filled multidose pen delivers 12 micrograms follitropin delta in 0.36 mL
One mL of solution contains 33.3 micrograms of follitropin delta

3. LIST OF EXCIPIENTS

Excipients: phenol, polysorbate 20, L-methionine, sodium sulphate decahydrate, disodium phosphate dodecahydrate, concentrated phosphoric acid, sodium hydroxide, water for injections

4. PHARMACEUTICAL FORM AND CONTENTS

Solution for injection in a pre-filled pen
1 multidose pre-filled pen with 3 pen injection needles

5. METHOD AND ROUTE(S) OF ADMINISTRATION

Read the package leaflet before use.
Subcutaneous use.

6. SPECIAL WARNING THAT THE MEDICINAL PRODUCT MUST BE STORED OUT OF THE SIGHT AND REACH OF CHILDREN

Keep out of the sight and reach of children.

7. OTHER SPECIAL WARNING(S), IF NECESSARY

8. EXPIRY DATE

EXP
After first use: Use within 28 days. Store at or below 25 °C.

9. SPECIAL STORAGE CONDITIONS

Store in a refrigerator. Do not freeze.
Store in the original package in order to protect from light.
The medicine may be stored at or below 25 °C for up to 3 months including the period after first use. It must be discarded afterwards.

10. SPECIAL PRECAUTIONS FOR DISPOSAL OF UNUSED MEDICINAL PRODUCTS OR WASTE MATERIALS DERIVED FROM SUCH MEDICINAL PRODUCTS, IF APPROPRIATE

11. NAME AND ADDRESS OF THE MARKETING AUTHORISATION HOLDER

Ferring Pharmaceuticals A/S
Amager Strandvej 405
2770 Kastrup
Denmark

12. MARKETING AUTHORISATION NUMBER(S)

EU/1/16/1150/004

13. BATCH NUMBER

Batch

14. GENERAL CLASSIFICATION FOR SUPPLY

15. INSTRUCTIONS ON USE

16. INFORMATION IN BRAILLE

REKOVELLE 12 micrograms/0.36 mL

17. UNIQUE IDENTIFIER – 2D BARCODE

2D barcode carrying the unique identifier included.

18. UNIQUE IDENTIFIER - HUMAN READABLE DATA

PC
SN
NN

MINIMUM PARTICULARS TO APPEAR ON SMALL IMMEDIATE PACKAGING UNITS

PRE-FILLED PEN

1. NAME OF THE MEDICINAL PRODUCT AND ROUTE(S) OF ADMINISTRATION

REKOVELLE 12 micrograms/0.36 mL injection
follitropin delta
Subcutanous use

2. METHOD OF ADMINISTRATION

3. EXPIRY DATE

EXP
After first use: Use within 28 days. Store at or below 25 °C.

4. BATCH NUMBER

Batch

5. CONTENTS BY WEIGHT, BY VOLUME OR BY UNIT

12 mcg/0.36 mL

6. OTHER

PARTICULARS TO APPEAR ON THE OUTER PACKAGING

OUTER CARTON

1. NAME OF THE MEDICINAL PRODUCT

REKOVELLE 36 micrograms/1.08 mL solution for injection in a pre-filled pen
follitropin delta

2. STATEMENT OF ACTIVE SUBSTANCE(S)

Each pre-filled multidose pen delivers 36 micrograms follitropin delta in 1.08 mL
One mL of solution contains 33.3 micrograms of follitropin delta

3. LIST OF EXCIPIENTS

Excipients: phenol, polysorbate 20, L-methionine, sodium sulphate decahydrate, disodium phosphate dodecahydrate, concentrated phosphoric acid, sodium hydroxide, water for injections

4. PHARMACEUTICAL FORM AND CONTENTS

Solution for injection in a pre-filled pen
1 multidose pre-filled pen with 9 pen injection needles

5. METHOD AND ROUTE(S) OF ADMINISTRATION

Read the package leaflet before use.
Subcutaneous use.

6. SPECIAL WARNING THAT THE MEDICINAL PRODUCT MUST BE STORED OUT OF THE SIGHT AND REACH OF CHILDREN

Keep out of the sight and reach of children.

7. OTHER SPECIAL WARNING(S), IF NECESSARY

8. EXPIRY DATE

EXP
After first use: Use within 28 days. Store at or below 25 °C.

9. SPECIAL STORAGE CONDITIONS

Store in a refrigerator. Do not freeze.
Store in the original package in order to protect from light.
The medicine may be stored at or below 25 °C for up to 3 months including the period after first use. It must be discarded afterwards.

10. SPECIAL PRECAUTIONS FOR DISPOSAL OF UNUSED MEDICINAL PRODUCTS OR WASTE MATERIALS DERIVED FROM SUCH MEDICINAL PRODUCTS, IF APPROPRIATE

11. NAME AND ADDRESS OF THE MARKETING AUTHORISATION HOLDER

Ferring Pharmaceuticals A/S
Amager Strandvej 405
2770 Kastrup
Denmark

12. MARKETING AUTHORISATION NUMBER(S)

EU/1/16/1150/005

13. BATCH NUMBER

Batch

14. GENERAL CLASSIFICATION FOR SUPPLY

15. INSTRUCTIONS ON USE

16. INFORMATION IN BRAILLE

REKOVELLE 36 micrograms/1.08 mL

17. UNIQUE IDENTIFIER – 2D BARCODE

2D barcode carrying the unique identifier included.

18. UNIQUE IDENTIFIER - HUMAN READABLE DATA

PC
SN
NN

MINIMUM PARTICULARS TO APPEAR ON SMALL IMMEDIATE PACKAGING UNITS

PRE-FILLED PEN

1. NAME OF THE MEDICINAL PRODUCT AND ROUTE(S) OF ADMINISTRATION

REKOVELLE 36 micrograms/1.08 mL injection
follitropin delta
Subcutanous use

2. METHOD OF ADMINISTRATION

3. EXPIRY DATE

EXP
After first use: Use within 28 days. Store at or below 25 °C.

4. BATCH NUMBER

Batch

5. CONTENTS BY WEIGHT, BY VOLUME OR BY UNIT

36 mcg/1.08 mL

6. OTHER

PARTICULARS TO APPEAR ON THE OUTER PACKAGING

OUTER CARTON

1. NAME OF THE MEDICINAL PRODUCT

REKOVELLE 72 micrograms/2.16 mL solution for injection in a pre-filled pen
follitropin delta

2. STATEMENT OF ACTIVE SUBSTANCE(S)

Each pre-filled multidose pen delivers 72 micrograms follitropin delta in 2.16 mL
One mL of solution contains 33.3 micrograms of follitropin delta

3. LIST OF EXCIPIENTS

Excipients: phenol, polysorbate 20, L-methionine, sodium sulphate decahydrate, disodium phosphate dodecahydrate, concentrated phosphoric acid, sodium hydroxide, water for injections

4. PHARMACEUTICAL FORM AND CONTENTS

Solution for injection in a pre-filled pen
1 multidose pre-filled pen with 15 pen injection needles

5. METHOD AND ROUTE(S) OF ADMINISTRATION

Read the package leaflet before use.
Subcutaneous use.

6. SPECIAL WARNING THAT THE MEDICINAL PRODUCT MUST BE STORED OUT OF THE SIGHT AND REACH OF CHILDREN

Keep out of the sight and reach of children.

7. OTHER SPECIAL WARNING(S), IF NECESSARY

8. EXPIRY DATE

EXP
After first use: Use within 28 days. Store at or below 25 °C.

9. SPECIAL STORAGE CONDITIONS

Store in a refrigerator. Do not freeze.
Store in the original package in order to protect from light.
The medicine may be stored at or below 25 °C for up to 3 months including the period after first use. It must be discarded afterwards.

10. SPECIAL PRECAUTIONS FOR DISPOSAL OF UNUSED MEDICINAL PRODUCTS OR WASTE MATERIALS DERIVED FROM SUCH MEDICINAL PRODUCTS, IF APPROPRIATE

11. NAME AND ADDRESS OF THE MARKETING AUTHORISATION HOLDER

Ferring Pharmaceuticals A/S
Amager Strandvej 405
2770 Kastrup
Denmark

12. MARKETING AUTHORISATION NUMBER(S)

EU/1/16/1150/006

13. BATCH NUMBER

Batch

14. GENERAL CLASSIFICATION FOR SUPPLY

15. INSTRUCTIONS ON USE

16. INFORMATION IN BRAILLE

REKOVELLE 72 micrograms/2.16 mL

17. UNIQUE IDENTIFIER – 2D BARCODE

2D barcode carrying the unique identifier included.

18. UNIQUE IDENTIFIER - HUMAN READABLE DATA

PC
SN
NN

MINIMUM PARTICULARS TO APPEAR ON SMALL IMMEDIATE PACKAGING UNITS

PRE-FILLED PEN

1. NAME OF THE MEDICINAL PRODUCT AND ROUTE(S) OF ADMINISTRATION

REKOVELLE 72 micrograms/2.16 mL injection
follitropin delta
Subcutanous use

2. METHOD OF ADMINISTRATION

3. EXPIRY DATE

EXP
After first use: Use within 28 days. Store at or below 25 °C.

4. BATCH NUMBER

Batch

5. CONTENTS BY WEIGHT, BY VOLUME OR BY UNIT

72 mcg/2.16 mL

6. OTHER

B. PACKAGE LEAFLET

Package leaflet: Information for the user

REKOVELLE 12 micrograms/0.36 mL solution for injection in a pre-filled pen
follitropin delta

Read all of this leaflet carefully before you start using this medicine because it contains important information for you.
- Keep this leaflet. You may need to read it again.
- If you have any further questions, ask your doctor or pharmacist.
- This medicine has been prescribed for you only. Do not pass it on to others. It may harm them, even if their signs of illness are the same as yours.
- If you get any side effects, talk to your doctor or pharmacist. This includes any possible side effects not listed in this leaflet. See section 4.

What is in this leaflet

1. What REKOVELLE is and what it is used for
2. What you need to know before you use REKOVELLE
3. How to use REKOVELLE
4. Possible side effects
5. How to store REKOVELLE
6. Contents of the pack and other information

1. What REKOVELLE is and what it is used for

REKOVELLE contains follitropin delta, a follicle stimulating hormone which belongs to the family of hormones called gonadotropins. Gonadotropins are involved in reproduction and fertility.

REKOVELLE is used in the treatment of female infertility and in women undergoing assisted reproduction programmes such as *in vitro* fertilisation (IVF) and intracytoplasmic sperm injection (ICSI). REKOVELLE stimulates the ovaries to grow and develop many egg sacs (‘follicles’), from which eggs are collected and fertilised in the laboratory.

2. What you need to know before you use REKOVELLE

Before starting treatment with this medicine, a doctor should check you and your partner for possible causes of your fertility problems.

Do not use REKOVELLE
- if you are allergic to follicle stimulating hormone or any of the other ingredients of this medicine (listed in section 6)
- if you have a tumour of the uterus, ovaries, breasts, pituitary gland or hypothalamus
- if you have enlarged ovaries or cysts on your ovaries (unless caused by polycystic ovarian disease)
- if you suffer from bleeding from the vagina without any known cause
- if you have had an early menopause
- if you have malformations of the sexual organs which make a normal pregnancy impossible
- if you have fibroids of the uterus which make a normal pregnancy impossible.

Warnings and precautions
Talk to your doctor before using REKOVELLE.

Ovarian hyperstimulation syndrome
Gonadotropins like this medicine may cause ovarian hyperstimulation syndrome. This is when your follicles develop too much and become large cysts.
Talk to your doctor if you:

- have abdominal pain, discomfort or swelling
- have nausea
- are vomiting
- get diarrhoea
- gain weight
- have difficulty in breathing

Your doctor may ask you to stop using this medicine (see section 4).
If the recommended dose and schedule of administration are followed, ovarian hyperstimulation syndrome is less likely.

Blood clotting problems (thromboembolic events)
Clots in the blood vessels (veins or arteries) are more likely in women who are pregnant. Infertility treatment can increase the risk of this happening, especially if you are overweight or you or someone in your family (blood relative) have a known blood clotting disease (thrombophilia). Tell your doctor if you think this applies to you.

Twisting of ovaries
There have been reports of twisting of ovaries (ovarian torsion) following assisted reproductive technology treatment. Twisting of the ovary could cut off the blood flow to the ovary.

Multiple pregnancy and birth defects
When undergoing assisted reproductive technology treatment the possibility of having a multiple pregnancy (such as twins) is mainly related to the number of embryos placed inside your womb, the quality of the embryos, and your age. Multiple pregnancy may lead to medical complications for you and your babies. Furthermore, the risk of birth defects may be slightly higher following infertility treatment, which is thought to be due to characteristics of the parents (such as your age, and your partner’s sperm characteristics) and multiple pregnancy.

Pregnancy loss
When undergoing assisted reproductive technology treatment, you are more likely to have a miscarriage than if you conceive naturally.

Pregnancy outside the uterus (ectopic pregnancy)
When undergoing assisted reproductive technology treatment, you are more likely to have a pregnancy outside the uterus (ectopic pregnancy) than if you conceive naturally. If you have a history of tubal disease, you have an increased risk of ectopic pregnancy.

Ovarian and other reproductive system tumours
There have been reports of ovarian and other reproductive system tumours in women who had undergone infertility treatment. It is not known if treatment with fertility medicines increase the risk of these tumours in infertile women.

Other medical conditions
Before starting to use this medicine, tell your doctor if:

- you have been told by another doctor that pregnancy would be dangerous for you
- you have kidney or liver disease

Children and adolescents (under 18 years of age)
This medicine is not indicated in children and adolescents.

Other medicines and REKOVELLE
Tell your doctor if you are using, have recently used or might use any other medicines.

Pregnancy and breast-feeding
Do not use this medicine if you are pregnant or breast-feeding.

Driving and using machines
This medicine does not affect your ability to drive and use machines.

REKOVELLE contains sodium
This medicine contains less than 1 mmol sodium (23 mg) per dose, that is to say essentially "sodium-free".

3. How to use REKOVELLE

Always use this medicine exactly as your doctor has told you and at the dose your doctor has told you. Check with your doctor if you are not sure.

The REKOVELLE dose for your first treatment cycle will be calculated by your doctor using the level of anti-Müllerian hormone (AMH, a marker of how your ovaries will respond to stimulation with gonadotropins) in your blood and your body weight. Therefore the AMH result from a blood sample (taken within the last 12 months) should be available before you start treatment. Your body weight will also be measured before you start treatment. The REKOVELLE dose is stated in micrograms.

The REKOVELLE dose is fixed for the whole treatment period with no adjustments to increase or decrease your daily dose. Your doctor will monitor the effect of REKOVELLE treatment, and treatment is stopped when an appropriate number of egg sacs are present. In general, you will be given a single injection of a medicine called human chorionic gonadotrophin (hCG) at a dose of 250 micrograms or 5,000 IU for final development of the follicles.

If your body´s response to treatment is too weak or too strong, your doctor may decide to stop treatment with REKOVELLE. For the next treatment cycle, your doctor will in this case give you either a higher or a lower daily dose of REKOVELLE than before.

How are injections given
The instructions for using the pre-filled pen must be followed carefully. Do not use the pre-filled pen if the solution contains particles or if the solution does not look clear.

The first injection of this medicine should be given under the supervision of a doctor or a nurse. Your doctor will decide if you can give yourself further doses of this medicine at home, but only after receiving adequate training.

This medicine is to be given by injection just under the skin (subcutaneously) usually in the abdomen. The pre-filled pen may be used for several injections.

If you use more REKOVELLE than you should
The effects of taking too much of this medicine are unknown. Ovarian hyperstimulation syndrome may possibly occur, which is described in section 4.

If you forget to use REKOVELLE
Do not take a double dose to make up for a forgotten dose. Please contact your doctor as soon as you notice that you forgot a dose.

If you have any further questions on the use of this medicine, ask your doctor.

4. Possible side effects

Like all medicines, this medicine can cause side effects, although not everybody gets them.

Serious side effects:
Hormones used in the treatment of infertility such as this medicine may cause a high level of activity in the ovaries (ovarian hyperstimulation syndrome). Symptoms may include pain, discomfort or swelling of the abdomen, nausea, vomiting, diarrhoea, weight gain or difficulty breathing. If you have any of these symptoms you should contact a doctor immediately.

The risk of having a side effect is described by the following categories:

Common (may affect up to 1 in 10 people):
- Headache
- Nausea
- Ovarian hyperstimulation syndrome (see above)
- Pelvic pain and discomfort, including of ovarian origin
- Tiredness (fatigue)

Uncommon (may affect up to 1 in 100 people):
- Mood swings
- Sleepiness/drowsiness
- Dizziness
- Diarrhoea
- Vomiting
- Constipation
- Discomfort of the abdomen
- Vaginal bleeding
- Breast discomfort (including breast pain, breast swelling, breast tenderness and/or nipple pain)

Reporting of side effects
If you get any side effects, talk to your doctor or pharmacist. This includes any possible side effects not listed in this leaflet. You can also report side effects directly via the national reporting system listed in Appendix V. By reporting side effects you can help provide more information on the safety of this medicine.

5. How to store REKOVELLE

Keep this medicine out of the sight and reach of children.

Do not use this medicine after the expiry date which is stated on the pre-filled pen label and carton after EXP. The expiry date refers to the last day of that month.
Store in refrigerator (2 °C - 8 °C). Do not freeze.
Store in the original package in order to protect from light.
REKOVELLE may be stored at or below 25 °C for up to 3 months including the period after first use. It must not be refrigerated again and must be discarded if it has not been used after 3 months.

After first use: 28 days when stored at or below 25 °C.

At the end of the treatment any unused solution must be discarded.

Do not throw away any medicines via wastewater or household waste. Ask your pharmacist how to throw away medicines you no longer use. These measures will help protect the environment.

6. Contents of the pack and other information

What REKOVELLE contains

- The active substance is follitropin delta.
  Each pre-filled pen with multidose cartridge contains 12 micrograms of follitropin delta in 0.36 millilitre of solution. One millilitre of solution contains 33.3 micrograms of follitropin delta in each millilitre of solution.
- The other ingredients are phenol, polysorbate 20, L-methionine, sodium sulphate decahydrate, disodium phosphate dodecahydrate, concentrated phosphoric acid, sodium hydroxide and water for injections.

What REKOVELLE looks like and contents of the pack

REKOVELLE is a clear and colourless solution for injection in a pre-filled pen (injection). It is available in packs of 1 pre-filled pen and 3 pen injection needles.

Marketing Authorisation Holder

Ferring Pharmaceuticals A/S
Amager Strandvej 405
2770 Kastrup
Denmark

Manufacturer

Ferring GmbH
Wittland 11
D-24109 Kiel
Germany

For any information about this medicine, please contact the local representative of the Marketing Authorisation Holder:

België/Belgique/Belgien
Ferring N.V.
Tel/Tél: +32 53 72 92 00
ferringnvsa@ferring.be

България
Фармонт ЕООД
Тел: +359 2 807 5022
farmont@farmont.bg

Česká republika
Ferring Pharmaceuticals CZ s.r.o.
Tel: +420 234 701 333
cz1-info@ferring.com

Danmark
Ferring Lægemidler A/S
Tlf: +45 88 16 88 17

Deutschland
Ferring Arzneimittel GmbH
Tel: +49 431 5852 0
info-service@ferring.de

Lietuva
CentralPharma Communications UAB
Tel: +370 5 243 0444
centralpharma@centralpharma.lt

Luxembourg/Luxemburg
Ferring N.V.
Belgique/Belgien
Tel/Tél: +32 53 72 92 00
ferringnvsa@ferring.be

Magyarország
Ferring Magyarország Gyógyszerkereskedelmi Kft.
Tel: +36 1 236 3800
ferring@ferring.hu

Malta
E.J. Busuttil Ltd.
Tel: +356 21447184
info@ejbusuttil.com

Nederland
Ferring B.V.
Tel: +31 235680300
infoNL@ferring.com

Eesti
CentralPharma Communications OÜ
Tel: +372 601 5540
centralpharma@centralpharma.ee

Ελλάδα
Ferring Ελλάς ΜΕΠΕ
Τηλ: +30 210 68 43 449

España
Ferring S.A.U.
Tel: +34 91 387 70 00
Registros@ferring.com

France
Ferring S.A.S.
Tél: +33 1 49 08 67 60
information.medicale@ferring.com

Hrvatska
Clinres farmacija d.o.o.
Tel: +385 1 2396 900
info@clinres-farmacija.hr

Ireland
Ferring Ireland Ltd.
Tel: +353 1 4637355
EnquiriesIrelandMailbox@ferring.com

Ísland
Vistor hf.
Sími: +354 535 70 00

Italia
Ferring S.p.A.
Tel: +39 02 640 00 11

Κύπρος
A.Potamitis Medicare Ltd
Τηλ: +357 22583333
a.potamitismedicare@cytanet.com.cy

Latvija
CentralPharma Communications SIA
Tālr: +371 674 50497
centralpharma@centralpharma.lv

Norge
Ferring Legemidler AS
Tlf: +47 22 02 08 80
mail@oslo.ferring.com

Österreich
Ferring Arzneimittel Ges.m.b.H
Tel: +43 1 60 8080
office@ferring.at

Polska
Ferring Pharmaceuticals Poland Sp. z o.o.
Tel: +48 22 246 06 80
PL0-Recepcja@ferring.com

Portugal
Ferring Portuguesa – Produtos Farmacêuticos,
Sociedade Unipessoal, Lda.
Tel: +351 21 940 51 90

România
Ferring Pharmaceuticals Romania SRL
Tel: +40 356 113 270

Slovenija
SALUS, Veletrgovina, d.o.o.
Tel: +386 1 5899 100
regulatory@salus.si

Slovenská republika
Ferring Slovakia s.r.o.
Tel: +421 2 54 416 010
SK0-Recepcia@ferring.com

Suomi/Finland
Ferring Lääkkeet Oy
Puh/Tel: +358 207 401 440
info@ferring.fi

Sverige
Ferring Läkemedel AB
Tel: +46 40 691 69 00
info@ferring.se

United Kingdom (Northern Ireland)
Ferring Ireland Ltd.
Tel.: +353 14637355
EnquiriesIrelandMailbox@ferring.com

This leaflet was last revised in .

Detailed information on this medicine is available on the European Medicines Agency web site: http://www.ema.europa.eu.

Instructions For Use

REKOVELLE pre-filled pen
follitropin delta

Your healthcare provider should show you how to prepare and inject REKOVELLE the right way before you inject it for the first time.
Do not try to inject yourself until you have been shown the right way to give your injections by your healthcare provider.
Read this booklet completely before using your REKOVELLE pre-filled pen and each time you get a new pen. There may be new information. Follow the instructions carefully even if you have used a similar injection pen before. Using the pen incorrectly could result in receiving an incorrect dose of medicine.
Call your healthcare provider (doctor, nurse or pharmacist) if you have any questions about how to give your REKOVELLE injection.

The REKOVELLE pre-filled pen is a disposable, dial-a-dose pen that can be used to give more than 1 dose of REKOVELLE. The pen is available in 3 different strengths:

- 12 micrograms/0.36 mL
- 36 micrograms/1.08 mL
- 72 micrograms/2.16 mL

REKOVELLE pre-filled pen and its parts

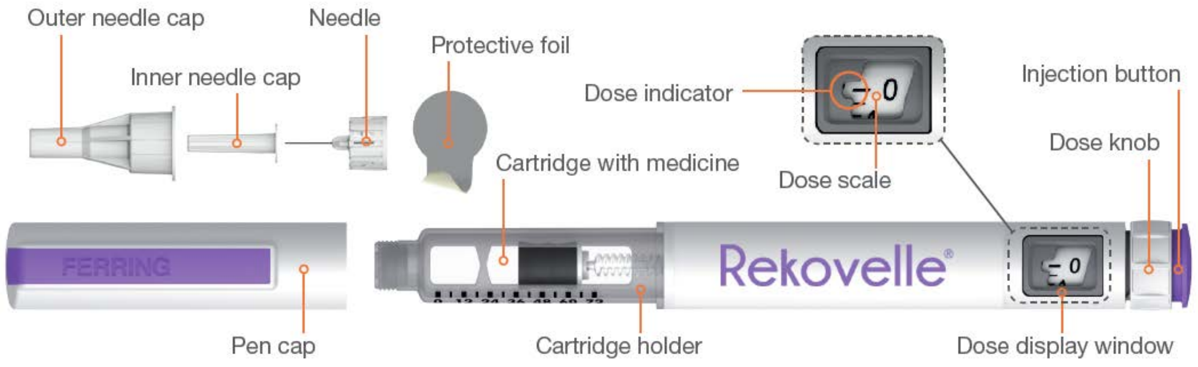

Instructions for use – REKOVELLE (follitropin delta) pre-filled pen

<u>Important information</u>

- The REKOVELLE pre-filled pen and the needles are for use by only one person and should not be shared with others.
- Use the pen only for the medical condition it is prescribed for and as directed by your healthcare provider.
- If you are blind or have poor eyesight and cannot read the dose scale on the pen, do not use this pen without help. Get help from a person with good eyesight who is trained to use the pen.
- Call your healthcare provider or local representative of the marketing authorisation holder (please refer to package leaflet for contact details) before giving your REKOVELLE injection if you have questions.

<u>Information about your REKOVELLE pre-filled pen</u>
The pen can be dialed to give doses from 0.33 micrograms to 20 micrograms of REKOVELLE in marked increments of 0.33 micrograms. See “Examples of how to dial a dose” at Page 20 to 21[1].

- The dose scale of the pen is numbered from 0 to 20 micrograms.
- Each number is separated by two lines, each line is equal to one increment of 0.33 micrograms.

- When turning the dial to your dose, you will hear a click sound and feel resistance on the dial for each increment to help you dial the correct dose.

Cleaning
- If needed, the outside of your pen may be cleaned with a cloth moistened with water.
- Do not put the pen in water or any other liquid.

Storage
- Always store the pen with the pen cap on and without a needle attached.
- Do not use the pen after the expiry date (EXP) printed on the pen label.
- Do not store the pen in extreme temperatures, direct sunlight or very cold conditions, such as in a car or freezer.
- Store the pen out of the reach of children and anyone who has not been trained to use the pen.

Before use:
- Store the pen in a refrigerator at 2 °C to 8 °C. Do not freeze.
- If stored outside the refrigerator (at or below 25 °C), the pen will last up to 3 months including the in-use period. Throw away (dispose of) the pen if it has not been used after 3 months.

After first use (in-use period):
- The pen may be stored for up to 28 days at or below 25°C. Do not freeze.

Supplies you will need to give your REKOVELLE injection

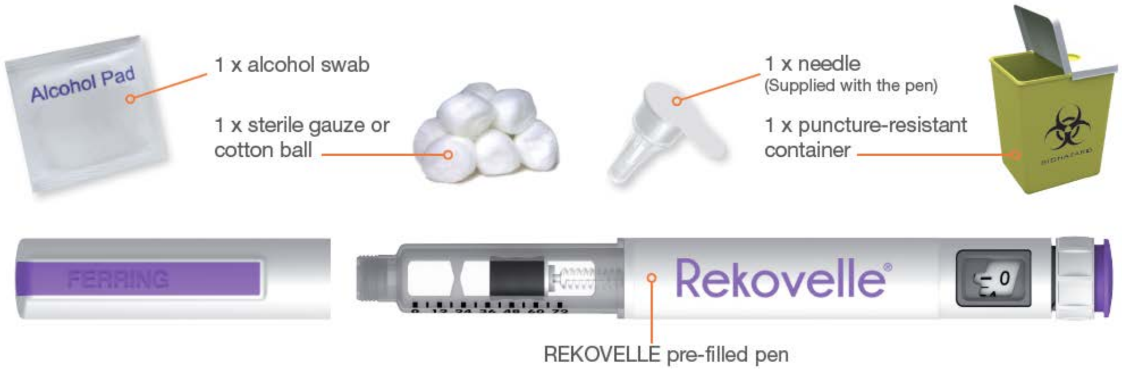

Before use – (Step 1)

Step 1:

- Wash your hands.
- Check the pen to see that it is not damaged. Do not use the pen if it is damaged.
- Check the pen (cartridge) to see that the medicine is clear and does not contain particles. Do not use a pen with particles or unclear medicine in the cartridge.
- Make sure you have the correct pen with correct strength.
- Check the expiration on the pen label.

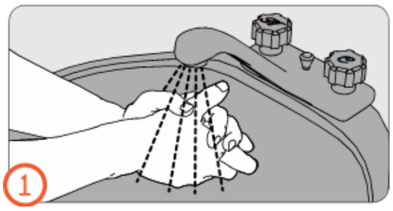

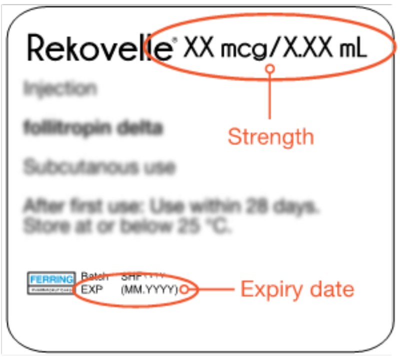

Attaching needle – (Step 2 to 6)

Important:

- Always use a new needle for each injection.
- Only use the single-use click-on needles supplied with the pen.

Step 2:

- Pull off the pen cap.

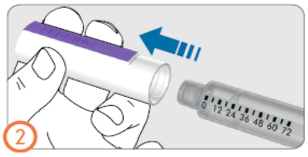

Step 3:

- Pull off the protective foil from the needle.

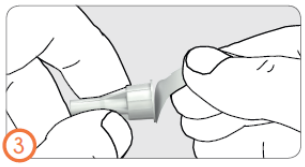

Step 4:

- Click on the needle.
- You will hear or feel a click when the needle is safely on.
- You may also screw on the needle. When you feel a light resistance it is safely on.

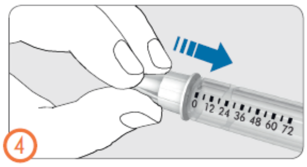

Step 5:

- Pull off the outer needle cap.
- Do not throw the outer needle cap away. You will need it to throw away (dispose of) the needle after injecting the medicine.

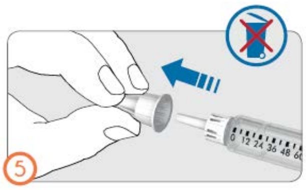

Step 6:

- Pull off the inner needle cap and throw it away.

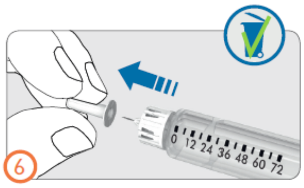

Priming – (Step 7 to 9)

- Before using the pen for the first time, you need to remove air bubbles from the cartridge (Priming) to receive the correct dose of medicine.
- Only prime your pen the first time you use it.
- Perform Step 7 to 9 even if you do not see air bubbles.
- If the pen has already been used proceed directly to Step 10.

<u>Step 7:</u>
- Turn the dose knob clockwise until a symbol of a droplet lines up with the dose indicator.
- If you dial the incorrect priming dose, the priming dose can be corrected either up or down without loss of medicine by turning the dose knob in either direction until the symbol of a droplet lines up with the dose indicator.

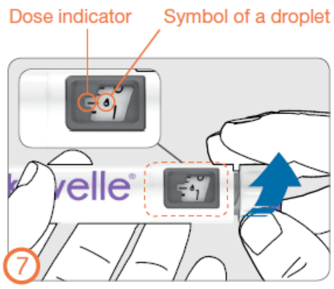

<u>Step 8:</u>
- Hold the pen with the needle pointing upwards.
- Tap with your finger on the cartridge holder to make any air bubbles in the cartridge rise to the top of the cartridge.

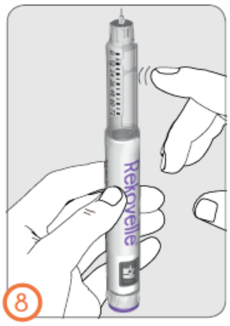

<u>Step 9:</u>
- With the needle still pointing upwards (away from the face) press in the injection button all the way in until you see the number ‘0’ lined up with the dose indicator.
- Check that a droplet of liquid appears at the tip of the needle.
- If no droplet(s) appear repeat Steps 7 to 9 (Priming) until a droplet appears.
- If no droplet appears after 5 tries, remove the needle (See Step 13), attach a new needle (See Steps 3 to 6), and repeat priming (See Steps 7 to 9).
- If you still do not see a droplet after using a new needle, try a new pen.

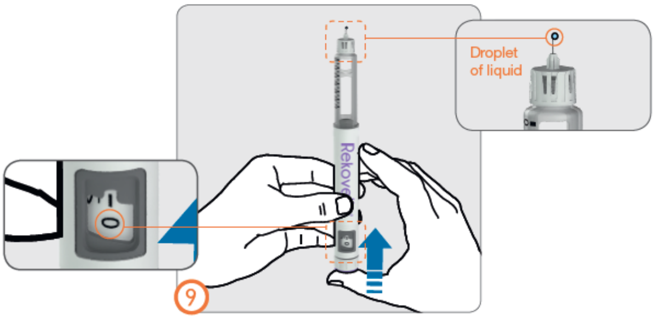

Dialing the dose – (Step 10)

See "Examples of how to dial a dose" at Page 20 to 21[1].

Step 10:
- Turn the dose knob clockwise until the prescribed dose lines up with the dose indicator in the dose display window.
- The dose can be corrected either up or down without loss of medicine by turning the dose knob in either direction until the correct dose lines up with the dose indicator.
- Do not press the injection button when dialing the dose to avoid loss of medicine.

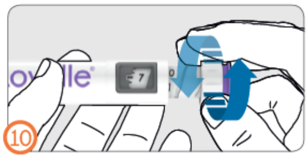

Split-dosing:
- You may need more than one pen to complete your prescribed dose.
- If you are not able to dial your complete dose, this means there is not enough medicine left in the pen. You will need to give a split-dose injection or throw away (dispose of) your current pen and use a new pen for your injection.

See "Giving a split-dose of REKOVELLE" at Page 22 to 23[1] for examples of how to calculate and record your split dose.

Injecting the dose – (Step 11 to 12)

Important:
- Do not use the pen if the medicine contains particles or if the medicine is not clear.
- Read Step 11 and 12 at Page 14 to 15[1] before giving your injection.
- This medicine should be given by injection just under the skin (subcutaneously) in the stomach-area (abdomen).
- Use a new injection site for each injection to lower the risk of skin reactions as redness and irritation.
- Do not inject into an area that is sore (tender), bruised, red, hard, scarred or where you have stretch marks.

Step 11 and 12:
- Wipe the skin of your injection site with an alcohol swab to clean it. Do not touch this area again before you give your injection.
- Hold the pen so the dose display window is visible during injection.
- Pinch your skin and insert the needle straight into your skin as shown by your healthcare provider. Do not touch the injection button yet.
- After the needle is inserted, place your thumb on the injection button.

- Press the injection button all the way in and hold.
- Keep pressing the injection button in and when you see the number ‘0’ lined up with the dose indicator, wait for 5 seconds (slowly count to 5). This will make sure you get your full dose.

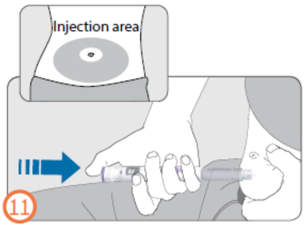

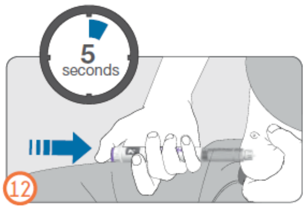

- After pressing in the injection button for 5 seconds, release the injection button. Then slowly remove the needle from the injection site by pulling it straight out of the skin.
- If blood appears at the injection site, press a gauze pad or cotton ball lightly to the injection site.

Note:

- Do not tilt the pen during injection and removal from skin.
- Tilting the pen can cause the needle to bend or break off.
- If a broken needle remains stuck in the body or remains under the skin, get medical help right away.

Disposal of Needle – (Step 13)

<u>Step 13:</u>

- Carefully replace the outer needle cap over the needle with a firm push (A).
- Unscrew the needle in counter-clockwise direction to remove the needle from the pen (B+C).
- Throw away (dispose of) the used needle carefully (D).
- See “Disposal” at Page 18[1].

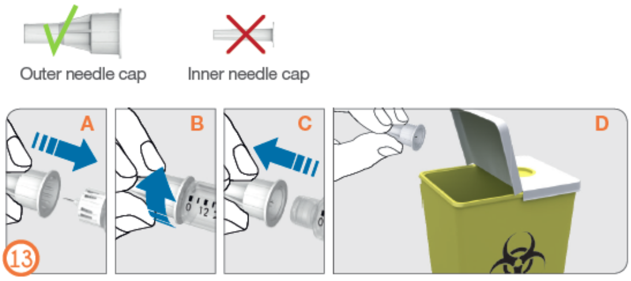

Note:

- Always remove the needle after every use. The needles are for single-use only.
- Do not store the pen with the needle attached.

Replace pen cap – (Step 14)

<u>Step 14:</u>

- Firmly replace the pen cap on the pen for protection between injections.

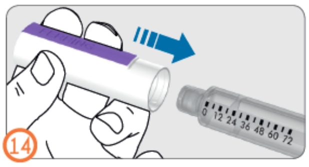

Note:

- The pen cap will not fit over a needle.
- If you will give a split-dose injection, only throw away (dispose of) the pen when it is empty.
- If you will use a new pen to give your full prescribed dose instead of giving a split-dose injection, throw away (dispose of) your pen when there is not enough medicine in the pen for a full dose.
- Keep the pen cap on the pen when it is not in use.

Disposal

Needles:
Put your used needles in a puncture-resistant container, such as a sharps disposal container right away after use. Do not throw away (dispose of) your used sharps disposal container in your household trash.

If you do not have a sharps disposal container, you may use a household container that is:
- made of a heavy-duty plastic,
- can be closed with a tight-fitting, puncture-resistant lid, without sharps being able to come out,
- upright and stable during use,
- leak-resistant, and
- properly labeled to warn of hazardous waste inside the container.

REKOVELLE pre-filled pens:
- Throw away (dispose of) your used pens in accordance with local waste regulations.

Examples of how to dial a dose

<u>Examples of how to dial a dose using your REKOVELLE pre-filled pen</u>
The chart below shows examples of prescribed doses, how to dial the examples of prescribed doses, and what the dose display window looks like for the prescribed doses.

**Examples of prescribed dose (in micrograms)**	**Dose to dial on pen**	**Dose display window for example of prescribed dose**
0.33	0 and 1 line<br>(Dial to 0 plus 1 click)	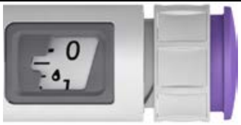
0.66 (priming dose)	0 and 2 lines<br>(Dial to 0 plus 2 clicks)	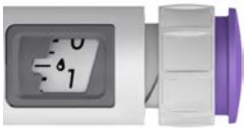
2.33	2 and 1 line<br>(Dial to 2 plus 1 click)	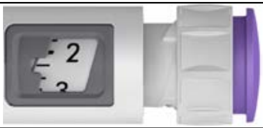
11.00	11<br>(Dial to 11)	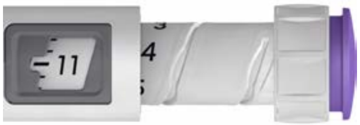
12.33	12 and 1 line<br>(Dial to 12 plus 1 click)	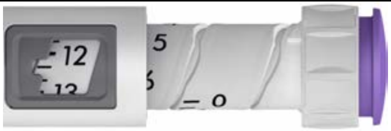
18.66	18 and 2 lines<br>(Dial to 18 plus 2 clicks)	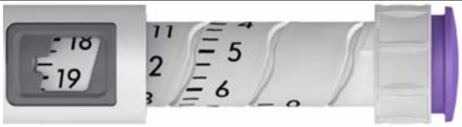
20.00	20 (Dial to 20)	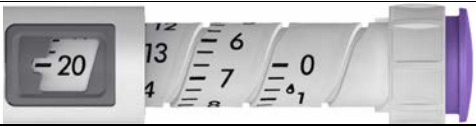

Giving a split-dose of REKOVELLE

If you are not able to dial the full prescribed dose in your pen, this means that there is not enough medicine left in the pen to give the full dose. You will need to give part of your prescribed dose using

your current pen and the remainder of the dose using a new pen (split-dose injection) or you may throw away (dispose of) the pen you are using and use a new pen to give your full prescribed dose in 1 injection. If you decide to give a split-dose injection, follow these instructions and write down how much medicine to give using the split-dose diary on Page 23[1].

- Column A shows an example of a prescribed dose. Write down your prescribed dose in column A.
- Column B shows an example of the dose that is left in the pen (this is equal to what you are able to dial).
- Write down the dose that is left in your pen in column B. Give the injection using the rest of the medicine that is left in your pen.
- Prepare and prime a new pen (Step 1 to 9).
- Calculate and write down the remaining dose to inject in column C by subtracting the number in column B from the number in column A. Use a calculator to check your math if needed.
- See "Examples of how to dial a dose" at Page 20 to 21[1] if needed.
- Doses should be rounded off to nearest increment, X.00, X.33 or X.66 micrograms. For example, if the number in column C is 5.34, round your remaining dose to 5.33. If the number in column C is 9.67, round your remaining dose to 9.66.
- Call your healthcare provider if you have questions about how to calculate your split-dose.
- Inject the remaining dose of medicine (the number in column C) using your new pen to complete your prescribed dose.

<u>Split-dose diary</u>

A<br>Prescribed Dose	B<br>Dose left in pen<br>(Dose shown at dose indicator in dose display window)	C = A minus B<br>Dose to inject on new pen<br>(Dose shown at dose indicator in dose display window)
11.33	*4.00 (4)*	*7.33 (7 and 1 line (Dial to 7 plus 1 click))*
12.66	*12.33 (12 and 1 line (12 plus 1 click))*	*0.33 (0 and 1 line (Dial to 0 plus 1 click))*
11.00	*3.00 (3)*	*8.00 (8 (Dial to 8))*
12.00	*6.66 (6 and 2 lines (6 plus 2 clicks))*	*Round 5.34 to 5.33 (5 and 1 line (Dial to 5 and 1 click))*
18.33	*8.66 (8 and 2 lines (8 plus 2 clicks))*	*Round 9.67 to 9.66 (9 and 2 lines (Dial to 9 plus 2 clicks))*

Frequently Asked Questions (FAQ)

1. Is the priming step necessary before each injection?
   - No. Priming must be performed only before giving the first injection with a new pen.

2. How do I know that the injection is complete?
   - The injection button is firmly pushed in all the way until it stops.
   - The number ‘0’ is lined up with the dose indicator.
   - You have slowly counted to 5 while you are still holding the injection button in and the needle is still in your skin.

3. Why do I have to count to 5 while holding the injection button?
   - Holding the injection button for 5 seconds allows for the full dose to be injected and absorbed under your skin.

4. What if the dose knob cannot be turned to the required dose?
   - The cartridge in the pen may not have enough medicine left to deliver the prescribed dose.
   - The pen does not allow you to dial a larger dose than the dose that is left in the cartridge.
   - You can inject the medicine left in the pen and complete the prescribed dose with a new pen (split-dose) or use a new pen to give the full prescribed dose.

Warnings

- Do not use a pen if it has been dropped or hit against hard surfaces.
- If the injection button is not easy to push in, do not use force. Change the needle. If the injection button still is not easy to push in after changing the needle, use a new pen.
- Do not try to repair a damaged pen. If a pen is damaged, contact your healthcare provider or local representative of the marketing authorisation holder (please refer to package leaflet for contact details).

Additional information

<u>Needles</u>
Needles are supplied with your pen. If you need additional needles contact your healthcare provider. Use only needles that come with your REKOVELLE pre-filled pen or that your healthcare provider prescribes.

<u>Contact</u>
If you have any questions or problems related to the pen, contact your healthcare provider or local representative of the marketing authorisation holder (please refer to package leaflet for contact details).

1. Page numbers refer to the printed Instructions For Use booklet and not to actual page numbers in this document.

Package leaflet: Information for the user

REKOVELLE 36 micrograms/1.08 mL solution for injection in a pre-filled pen
follitropin delta

Read all of this leaflet carefully before you start using this medicine because it contains important information for you.
- Keep this leaflet. You may need to read it again.
- If you have any further questions, ask your doctor or pharmacist.
- This medicine has been prescribed for you only. Do not pass it on to others. It may harm them, even if their signs of illness are the same as yours.
- If you get any side effects, talk to your doctor or pharmacist. This includes any possible side effects not listed in this leaflet. See section 4.

What is in this leaflet

1. What REKOVELLE is and what it is used for
2. What you need to know before you use REKOVELLE
3. How to use REKOVELLE
4. Possible side effects
5. How to store REKOVELLE
6. Contents of the pack and other information

1. What REKOVELLE is and what it is used for

REKOVELLE contains follitropin delta, a follicle stimulating hormone which belongs to the family of hormones called gonadotropins. Gonadotropins are involved in reproduction and fertility.

REKOVELLE is used in the treatment of female infertility and in women undergoing assisted reproduction programmes such as *in vitro* fertilisation (IVF) and intracytoplasmic sperm injection (ICSI). REKOVELLE stimulates the ovaries to grow and develop many egg sacs (‘follicles’), from which eggs are collected and fertilised in the laboratory.

2. What you need to know before you use REKOVELLE

Before starting treatment with this medicine, a doctor should check you and your partner for possible causes of your fertility problems.

Do not use REKOVELLE
- if you are allergic to follicle stimulating hormone or any of the other ingredients of this medicine (listed in section 6)
- if you have a tumour of the uterus, ovaries, breasts, pituitary gland or hypothalamus
- if you have enlarged ovaries or cysts on your ovaries (unless caused by polycystic ovarian disease)
- if you suffer from bleeding from the vagina without any known cause
- if you have had an early menopause
- if you have malformations of the sexual organs which make a normal pregnancy impossible
- if you have fibroids of the uterus which make a normal pregnancy impossible.

Warnings and precautions
Talk to your doctor before using REKOVELLE.

Ovarian hyperstimulation syndrome
Gonadotropins like this medicine may cause ovarian hyperstimulation syndrome. This is when your follicles develop too much and become large cysts.
Talk to your doctor if you:
- have abdominal pain, discomfort or swelling
- have nausea
- are vomiting
- get diarrhoea
- gain weight
- have difficulty in breathing

Your doctor may ask you to stop using this medicine (see section 4).
If the recommended dose and schedule of administration are followed, ovarian hyperstimulation syndrome is less likely.

Blood clotting problems (thromboembolic events)
Clots in the blood vessels (veins or arteries) are more likely in women who are pregnant. Infertility treatment can increase the risk of this happening, especially if you are overweight or you or someone in your family (blood relative) have a known blood clotting disease (thrombophilia). Tell your doctor if you think this applies to you.

Twisting of ovaries
There have been reports of twisting of ovaries (ovarian torsion) following assisted reproductive technology treatment. Twisting of the ovary could cut off the blood flow to the ovary.

Multiple pregnancy and birth defects
When undergoing assisted reproductive technology treatment the possibility of having a multiple pregnancy (such as twins) is mainly related to the number of embryos placed inside your womb, the quality of the embryos, and your age. Multiple pregnancy may lead to medical complications for you and your babies. Furthermore, the risk of birth defects may be slightly higher following infertility treatment, which is thought to be due to characteristics of the parents (such as your age, and your partner's sperm characteristics) and multiple pregnancy.

Pregnancy loss
When undergoing assisted reproductive technology treatment, you are more likely to have a miscarriage than if you conceive naturally.

Pregnancy outside the uterus (ectopic pregnancy)
When undergoing assisted reproductive technology treatment, you are more likely to have a pregnancy outside the uterus (ectopic pregnancy) than if you conceive naturally. If you have a history of tubal disease, you have an increased risk of ectopic pregnancy.

Ovarian and other reproductive system tumours
There have been reports of ovarian and other reproductive system tumours in women who had undergone infertility treatment. It is not known if treatment with fertility medicines increase the risk of these tumours in infertile women.

Other medical conditions
Before starting to use this medicine, tell your doctor if:
- you have been told by another doctor that pregnancy would be dangerous for you
- you have kidney or liver disease

Children and adolescents (under 18 years of age)
This medicine is not indicated in children and adolescents.

Other medicines and REKOVELLE
Tell your doctor if you are using, have recently used or might use any other medicines.

Pregnancy and breast-feeding
Do not use this medicine if you are pregnant or breast-feeding.

Driving and using machines
This medicine does not affect your ability to drive and use machines.

REKOVELLE contains sodium
This medicine contains less than 1 mmol sodium (23 mg) per dose, that is to say essentially "sodium-free".

3. How to use REKOVELLE

Always use this medicine exactly as your doctor has told you and at the dose your doctor has told you. Check with your doctor if you are not sure.

The REKOVELLE dose for your first treatment cycle will be calculated by your doctor using the level of anti-Müllerian hormone (AMH, a marker of how your ovaries will respond to stimulation with gonadotropins) in your blood and your body weight. Therefore the AMH result from a blood sample (taken within the last 12 months) should be available before you start treatment. Your body weight will also be measured before you start treatment. The REKOVELLE dose is stated in micrograms.

The REKOVELLE dose is fixed for the whole treatment period with no adjustments to increase or decrease your daily dose. Your doctor will monitor the effect of REKOVELLE treatment, and treatment is stopped when an appropriate number of egg sacs are present. In general, you will be given a single injection of a medicine called human chorionic gonadotrophin (hCG) at a dose of 250 micrograms or 5,000 IU for final development of the follicles.

If your body´s response to treatment is too weak or too strong, your doctor may decide to stop treatment with REKOVELLE. For the next treatment cycle, your doctor will in this case give you either a higher or a lower daily dose of REKOVELLE than before.

How are injections given
The instructions for using the pre-filled pen must be followed carefully. Do not use the pre-filled pen if the solution contains particles or if the solution does not look clear.

The first injection of this medicine should be given under the supervision of a doctor or a nurse. Your doctor will decide if you can give yourself further doses of this medicine at home, but only after receiving adequate training.

This medicine is to be given by injection just under the skin (subcutaneously) usually in the abdomen. The pre-filled pen may be used for several injections.

If you use more REKOVELLE than you should
The effects of taking too much of this medicine are unknown. Ovarian hyperstimulation syndrome may possibly occur, which is described in section 4.

If you forget to use REKOVELLE
Do not take a double dose to make up for a forgotten dose. Please contact your doctor as soon as you notice that you forgot a dose.

If you have any further questions on the use of this medicine, ask your doctor.

4. Possible side effects

Like all medicines, this medicine can cause side effects, although not everybody gets them.

Serious side effects:
Hormones used in the treatment of infertility such as this medicine may cause a high level of activity in the ovaries (ovarian hyperstimulation syndrome). Symptoms may include pain, discomfort or swelling of the abdomen, nausea, vomiting, diarrhoea, weight gain or difficulty breathing. If you have any of these symptoms you should contact a doctor immediately.

The risk of having a side effect is described by the following categories:

Common (may affect up to 1 in 10 people):
- Headache
- Nausea
- Ovarian hyperstimulation syndrome (see above)
- Pelvic pain and discomfort, including of ovarian origin
- Tiredness (fatigue)

Uncommon (may affect up to 1 in 100 people):
- Mood swings
- Sleepiness/drowsiness
- Dizziness
- Diarrhoea
- Vomiting
- Constipation
- Discomfort of the abdomen
- Vaginal bleeding
- Breast discomfort (including breast pain, breast swelling, breast tenderness and/or nipple pain)

Reporting of side effects
If you get any side effects, talk to your doctor or pharmacist. This includes any possible side effects not listed in this leaflet. You can also report side effects directly via the national reporting system listed in Appendix V. By reporting side effects you can help provide more information on the safety of this medicine.

5. How to store REKOVELLE

Keep this medicine out of the sight and reach of children.

Do not use this medicine after the expiry date which is stated on the pre-filled pen label and carton after EXP. The expiry date refers to the last day of that month.

Store in refrigerator (2 °C - 8 °C). Do not freeze.
Store in the original package in order to protect from light.
REKOVELLE may be stored at or below 25 °C for up to 3 months including the period after first use. It must not be refrigerated again and must be discarded if it has not been used after 3 months.

After first use: 28 days when stored at or below 25 °C.

At the end of the treatment any unused solution must be discarded.

Do not throw away any medicines via wastewater or household waste. Ask your pharmacist how to throw away medicines you no longer use. These measures will help protect the environment.

6. Contents of the pack and other information

What REKOVELLE contains

- The active substance is follitropin delta.
  Each pre-filled pen with multidose cartridge contains 36 micrograms of follitropin delta in 1.08 millilitre of solution. One millilitre of solution contains 33.3 micrograms of follitropin delta in each millilitre of solution.
- The other ingredients are phenol, polysorbate 20, L-methionine, sodium sulphate decahydrate, disodium phosphate dodecahydrate, concentrated phosphoric acid, sodium hydroxide and water for injections.

What REKOVELLE looks like and contents of the pack

REKOVELLE is a clear and colourless solution for injection in a pre-filled pen (injection). It is available in packs of 1 pre-filled pen and 9 pen injection needles.

Marketing Authorisation Holder

Ferring Pharmaceuticals A/S
Amager Strandvej 405
2770 Kastrup
Denmark

Manufacturer

Ferring GmbH
Wittland 11
D-24109 Kiel
Germany

For any information about this medicine, please contact the local representative of the Marketing Authorisation Holder:

België/Belgique/Belgien
Ferring N.V.
Tel/Tél: +32 53 72 92 00
ferringnvsa@ferring.be

България
Фармонт ЕООД
Тел: +359 2 807 5022
farmont@farmont.bg

Česká republika
Ferring Pharmaceuticals CZ s.r.o.
Tel: +420 234 701 333
cz1-info@ferring.com

Danmark
Ferring Lægemidler A/S
Tlf: +45 88 16 88 17

Deutschland
Ferring Arzneimittel GmbH
Tel: +49 431 5852 0
info-service@ferring.de

Lietuva
CentralPharma Communications UAB
Tel: +370 5 243 0444
centralpharma@centralpharma.lt

Luxembourg/Luxemburg
Ferring N.V.
Belgique/Belgien
Tel/Tél: +32 53 72 92 00
ferringnvsa@ferring.be

Magyarország
Ferring Magyarország Gyógyszerkereskedelmi Kft.
Tel: +36 1 236 3800
ferring@ferring.hu

Malta
E.J. Busuttil Ltd.
Tel: +356 21447184
info@ejbusuttil.com

Nederland
Ferring B.V.
Tel: +31 235680300
infoNL@ferring.com

Eesti
CentralPharma Communications OÜ
Tel: +372 601 5540
centralpharma@centralpharma.ee

Ελλάδα
Ferring Ελλάς ΜΕΠΕ
Τηλ: +30 210 68 43 449

España
Ferring S.A.U.
Tel: +34 91 387 70 00
Registros@ferring.com

France
Ferring S.A.S.
Tél: +33 1 49 08 67 60
information.medicale@ferring.com

Hrvatska
Clinres farmacija d.o.o.
Tel: +385 1 2396 900
info@clinres-farmacija.hr

Ireland
Ferring Ireland Ltd.
Tel: +353 1 4637355
EnquiriesIrelandMailbox@ferring.com

Ísland
Vistor hf.
Sími: +354 535 70 00

Italia
Ferring S.p.A.
Tel: +39 02 640 00 11

Κύπρος
A.Potamitis Medicare Ltd
Τηλ: +357 22583333
a.potamitismedicare@cytanet.com.cy

Latvija
CentralPharma Communications SIA
Tālr: +371 674 50497
centralpharma@centralpharma.lv

Norge
Ferring Legemidler AS
Tlf: +47 22 02 08 80
mail@oslo.ferring.com

Österreich
Ferring Arzneimittel Ges.m.b.H
Tel: +43 1 60 8080
office@ferring.at

Polska
Ferring Pharmaceuticals Poland Sp. z o.o.
Tel: +48 22 246 06 80
PL0-Recepcja@ferring.com

Portugal
Ferring Portuguesa – Produtos Farmacêuticos,
Sociedade Unipessoal, Lda.
Tel: +351 21 940 51 90

România
Ferring Pharmaceuticals Romania SRL
Tel: +40 356 113 270

Slovenija
SALUS, Veletrgovina, d.o.o.
Tel: +386 1 5899 100
regulatory@salus.si

Slovenská republika
Ferring Slovakia s.r.o.
Tel: +421 2 54 416 010
SK0-Recepcia@ferring.com

Suomi/Finland
Ferring Lääkkeet Oy
Puh/Tel: +358 207 401 440
info@ferring.fi

Sverige
Ferring Läkemedel AB
Tel: +46 40 691 69 00
info@ferring.se

United Kingdom (Northern Ireland)
Ferring Ireland Ltd.
Tel: +353 1 4637355
EnquiriesIrelandMailbox@ferring.com

This leaflet was last revised in .

Detailed information on this medicine is available on the European Medicines Agency web site: http://www.ema.europa.eu.

Instructions For Use

REKOVELLE pre-filled pen
follitropin delta

Your healthcare provider should show you how to prepare and inject REKOVELLE the right way before you inject it for the first time.
Do not try to inject yourself until you have been shown the right way to give your injections by your healthcare provider.
Read this booklet completely before using your REKOVELLE pre-filled pen and each time you get a new pen. There may be new information. Follow the instructions carefully even if you have used a similar injection pen before. Using the pen incorrectly could result in receiving an incorrect dose of medicine.
Call your healthcare provider (doctor, nurse or pharmacist) if you have any questions about how to give your REKOVELLE injection.

The REKOVELLE pre-filled pen is a disposable, dial-a-dose pen that can be used to give more than 1 dose of REKOVELLE. The pen is available in 3 different strengths:

- 12 micrograms/0.36 mL
- 36 micrograms/1.08 mL
- 72 micrograms/2.16 mL

REKOVELLE pre-filled pen and its parts

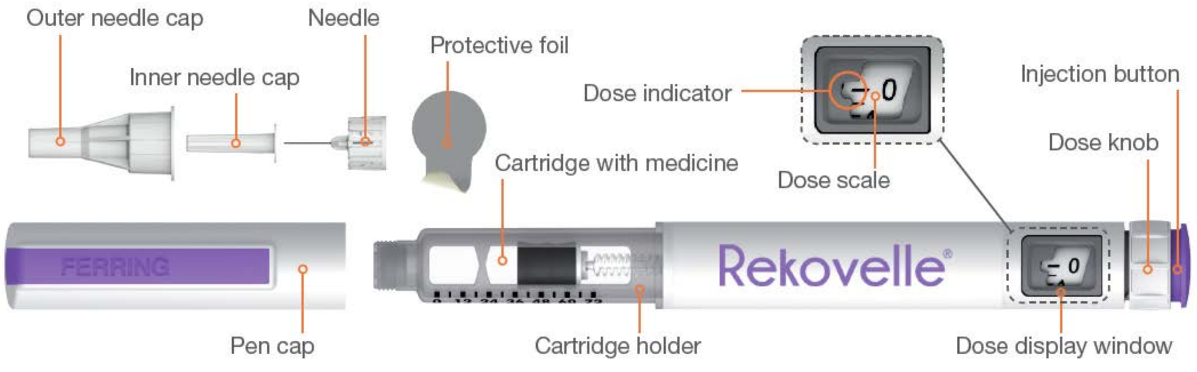

Instructions for use – REKOVELLE (follitropin delta) pre-filled pen

Important information

- The REKOVELLE pre-filled pen and the needles are for use by only one person and should not be shared with others.
- Use the pen only for the medical condition it is prescribed for and as directed by your healthcare provider.
- If you are blind or have poor eyesight and cannot read the dose scale on the pen, do not use this pen without help. Get help from a person with good eyesight who is trained to use the pen.
- Call your healthcare provider or local representative of the marketing authorisation holder (please refer to package leaflet for contact details) before giving your REKOVELLE injection if you have questions.

Information about your REKOVELLE pre-filled pen
The pen can be dialed to give doses from 0.33 micrograms to 20 micrograms of REKOVELLE in marked increments of 0.33 micrograms. See “Examples of how to dial a dose” at Page 20 to 21[1].

- The dose scale of the pen is numbered from 0 to 20 micrograms.
- Each number is separated by two lines, each line is equal to one increment of 0.33 micrograms.

- When turning the dial to your dose, you will hear a click sound and feel resistance on the dial for each increment to help you dial the correct dose.

Cleaning

- If needed, the outside of your pen may be cleaned with a cloth moistened with water.
- Do not put the pen in water or any other liquid.

Storage

- Always store the pen with the pen cap on and without a needle attached.
- Do not use the pen after the expiry date (EXP) printed on the pen label.
- Do not store the pen in extreme temperatures, direct sunlight or very cold conditions, such as in a car or freezer.
- Store the pen out of the reach of children and anyone who has not been trained to use the pen.

Before use:

- Store the pen in a refrigerator at 2 °C to 8 °C. Do not freeze.
- If stored outside the refrigerator (at or below 25 °C), the pen will last up to 3 months including the in-use period. Throw away (dispose of) the pen if it has not been used after 3 months.

After first use (in-use period):

- The pen may be stored for up to 28 days at or below 25°C. Do not freeze.

Supplies you will need to give your REKOVELLE injection

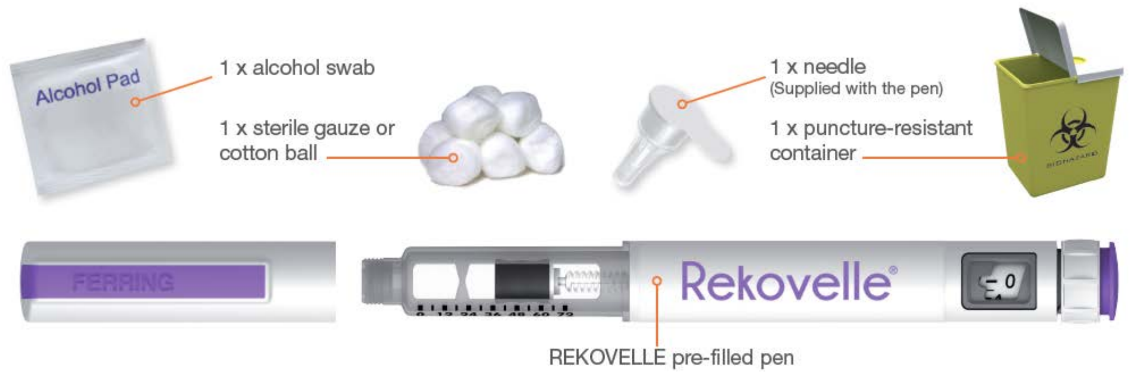

Before use – (Step 1)

Step 1:

- Wash your hands.
- Check the pen to see that it is not damaged. Do not use the pen if it is damaged.
- Check the pen (cartridge) to see that the medicine is clear and does not contain particles. Do not use a pen with particles or unclear medicine in the cartridge.
- Make sure you have the correct pen with correct strength.
- Check the expiration on the pen label.

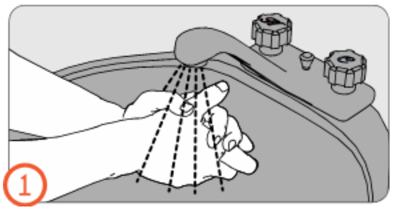

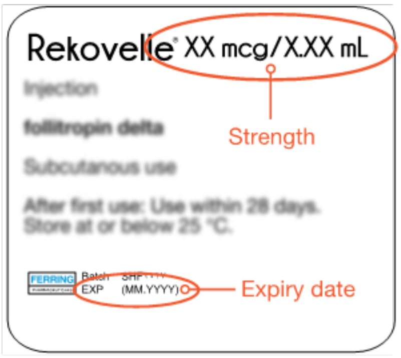

Attaching needle – (Step 2 to 6)

Important:

- Always use a new needle for each injection.
- Only use the single-use click-on needles supplied with the pen.

Step 2:

- Pull off the pen cap.

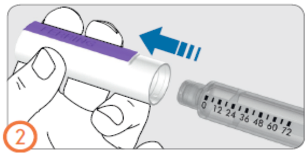

Step 3:

- Pull off the protective foil from the needle.

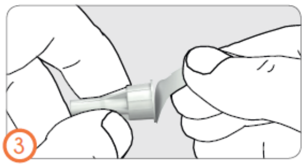

Step 4:

- Click on the needle.
- You will hear or feel a click when the needle is safely on.
- You may also screw on the needle. When you feel a light resistance it is safely on.

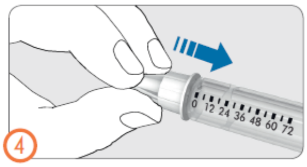

Step 5:

- Pull off the outer needle cap.
- Do not throw the outer needle cap away. You will need it to throw away (dispose of) the needle after injecting the medicine.

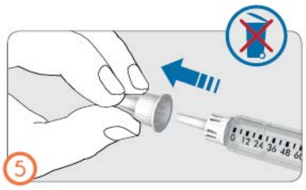

Step 6:

- Pull off the inner needle cap and throw it away.

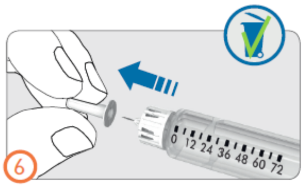

Priming – (Step 7 to 9)

- Before using the pen for the first time, you need to remove air bubbles from the cartridge (Priming) to receive the correct dose of medicine.
- Only prime your pen the first time you use it.
- Perform Step 7 to 9 even if you do not see air bubbles.
- If the pen has already been used proceed directly to Step 10.

Step 7:
- Turn the dose knob clockwise until a symbol of a droplet lines up with the dose indicator.
- If you dial the incorrect priming dose, the priming dose can be corrected either up or down without loss of medicine by turning the dose knob in either direction until the symbol of a droplet lines up with the dose indicator.

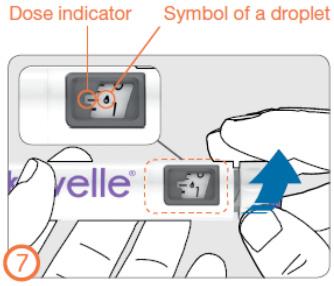

Step 8:
- Hold the pen with the needle pointing upwards.
- Tap with your finger on the cartridge holder to make any air bubbles in the cartridge rise to the top of the cartridge.

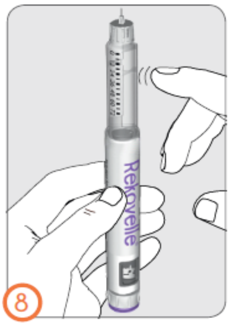

Step 9:
- With the needle still pointing upwards (away from the face) press in the injection button all the way in until you see the number ‘0’ lined up with the dose indicator.
- Check that a droplet of liquid appears at the tip of the needle.
- If no droplet(s) appear repeat Steps 7 to 9 (Priming) until a droplet appears.
- If no droplet appears after 5 tries, remove the needle (See Step 13), attach a new needle (See Steps 3 to 6), and repeat priming (See Steps 7 to 9).
- If you still do not see a droplet after using a new needle, try a new pen.

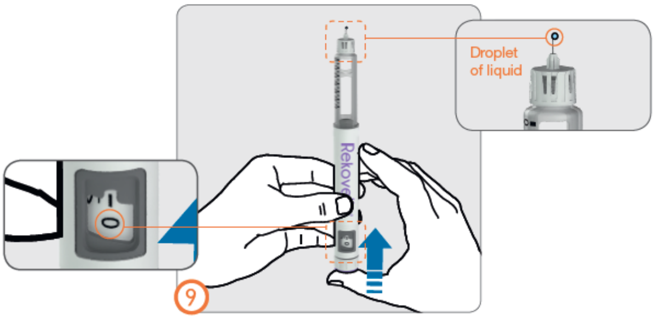

Dialing the dose – (Step 10)

See “Examples of how to dial a dose” at Page 20 to 21[1].

Step 10:

- Turn the dose knob clockwise until the prescribed dose lines up with the dose indicator in the dose display window.
- The dose can be corrected either up or down without loss of medicine by turning the dose knob in either direction until the correct dose lines up with the dose indicator.
- Do not press the injection button when dialing the dose to avoid loss of medicine.

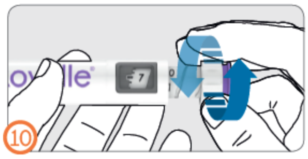

Split-dosing:

- You may need more than one pen to complete your prescribed dose.
- If you are not able to dial your complete dose, this means there is not enough medicine left in the pen. You will need to give a split-dose injection or throw away (dispose of) your current pen and use a new pen for your injection.

See “Giving a split-dose of REKOVELLE“ at Page 22 to 23[1] for examples of how to calculate and record your split dose.

Injecting the dose – (Step 11 to 12)

Important:

- Do not use the pen if the medicine contains particles or if the medicine is not clear.
- Read Step 11 and 12 at Page 14 to 15[1] before giving your injection.
- This medicine should be given by injection just under the skin (subcutaneously) in the stomach-area (abdomen).
- Use a new injection site for each injection to lower the risk of skin reactions as redness and irritation.
- Do not inject into an area that is sore (tender), bruised, red, hard, scarred or where you have stretch marks.

Step 11 and 12:

- Wipe the skin of your injection site with an alcohol swab to clean it. Do not touch this area again before you give your injection.
- Hold the pen so the dose display window is visible during injection.
- Pinch your skin and insert the needle straight into your skin as shown by your healthcare provider. Do not touch the injection button yet.
- After the needle is inserted, place your thumb on the injection button.

- Press the injection button all the way in and hold.
- Keep pressing the injection button in and when you see the number ‘0’ lined up with the dose indicator, wait for 5 seconds (slowly count to 5). This will make sure you get your full dose.

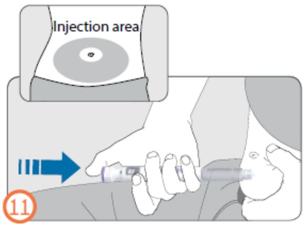

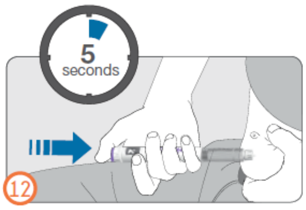

- After pressing in the injection button for 5 seconds, release the injection button. Then slowly remove the needle from the injection site by pulling it straight out of the skin.
- If blood appears at the injection site, press a gauze pad or cotton ball lightly to the injection site.

Note:
- Do not tilt the pen during injection and removal from skin.
- Tilting the pen can cause the needle to bend or break off.
- If a broken needle remains stuck in the body or remains under the skin, get medical help right away.

Disposal of Needle – (Step 13)

<u>Step 13:</u>
- Carefully replace the outer needle cap over the needle with a firm push (A).
- Unscrew the needle in counter-clockwise direction to remove the needle from the pen (B+C).
- Throw away (dispose of) the used needle carefully (D).
- See “Disposal” at Page 18[1].

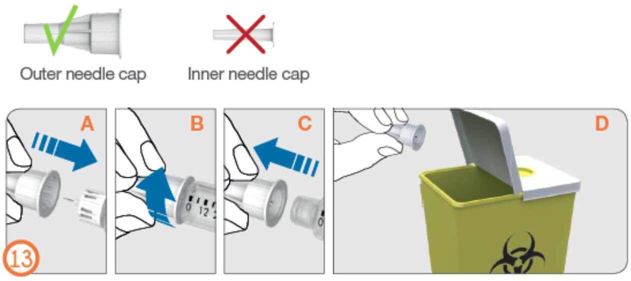

Note:
- Always remove the needle after every use. The needles are for single-use only.
- Do not store the pen with the needle attached.

Replace pen cap – (Step 14)

<u>Step 14:</u>
- Firmly replace the pen cap on the pen for protection between injections.

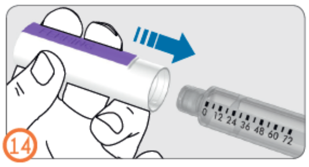

Note:

- The pen cap will not fit over a needle.
- If you will give a split-dose injection, only throw away (dispose of) the pen when it is empty.
- If you will use a new pen to give your full prescribed dose instead of giving a split-dose injection, throw away (dispose of) your pen when there is not enough medicine in the pen for a full dose.
- Keep the pen cap on the pen when it is not in use.

Disposal

Needles:
Put your used needles in a puncture-resistant container, such as a sharps disposal container right away after use. Do not throw away (dispose of) your used sharps disposal container in your household trash.

If you do not have a sharps disposal container, you may use a household container that is:
- made of a heavy-duty plastic,
- can be closed with a tight-fitting, puncture-resistant lid, without sharps being able to come out,
- upright and stable during use,
- leak-resistant, and
- properly labeled to warn of hazardous waste inside the container.

REKOVELLE pre-filled pens:
- Throw away (dispose of) your used pens in accordance with local waste regulations.

Examples of how to dial a dose

<u>Examples of how to dial a dose using your REKOVELLE pre-filled pen</u>
The chart below shows examples of prescribed doses, how to dial the examples of prescribed doses, and what the dose display window looks like for the prescribed doses.

**Examples of prescribed dose (in micrograms)**	**Dose to dial on pen**	**Dose display window for example of prescribed dose**
0.33	0 and 1 line<br>(Dial to 0 plus 1 click)	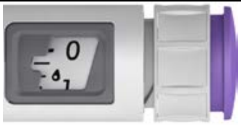
0.66 (priming dose)	0 and 2 lines<br>(Dial to 0 plus 2 clicks)	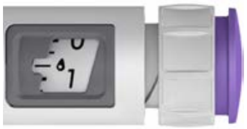
2.33	2 and 1 line<br>(Dial to 2 plus 1 click)	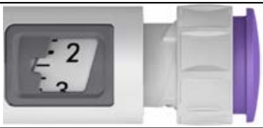
11.00	11<br>(Dial to 11)	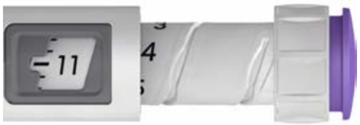
12.33	12 and 1 line<br>(Dial to 12 plus 1 click)	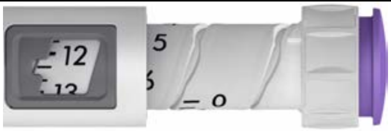
18.66	18 and 2 lines<br>(Dial to 18 plus 2 clicks)	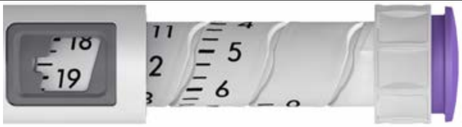
20.00	20 (Dial to 20)	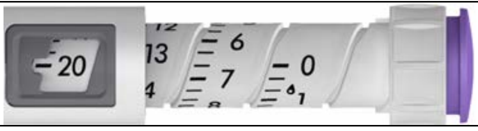

Giving a split-dose of REKOVELLE

If you are not able to dial the full prescribed dose in your pen, this means that there is not enough medicine left in the pen to give the full dose. You will need to give part of your prescribed dose using

your current pen and the remainder of the dose using a new pen (split-dose injection) or you may throw away (dispose of) the pen you are using and use a new pen to give your full prescribed dose in 1 injection. If you decide to give a split-dose injection, follow these instructions and write down how much medicine to give using the split-dose diary on Page 23[1].

- Column A shows an example of a prescribed dose. Write down your prescribed dose in column A.
- Column B shows an example of the dose that is left in the pen (this is equal to what you are able to dial).
- Write down the dose that is left in your pen in column B. Give the injection using the rest of the medicine that is left in your pen.
- Prepare and prime a new pen (Step 1 to 9).
- Calculate and write down the remaining dose to inject in column C by subtracting the number in column B from the number in column A. Use a calculator to check your math if needed.
- See "Examples of how to dial a dose" at Page 20 to 21[1] if needed.
- Doses should be rounded off to nearest increment, X.00, X.33 or X.66 micrograms. For example, if the number in column C is 5.34, round your remaining dose to 5.33. If the number in column C is 9.67, round your remaining dose to 9.66.
- Call your healthcare provider if you have questions about how to calculate your split-dose.
- Inject the remaining dose of medicine (the number in column C) using your new pen to complete your prescribed dose.

<u>Split-dose diary</u>

A<br>Prescribed Dose	B<br>Dose left in pen<br>(Dose shown at dose indicator in dose display window)	C = A minus B<br>Dose to inject on new pen<br>(Dose shown at dose indicator in dose display window)
11.33	*4.00 (4)*	*7.33 (7 and 1 line (Dial to 7 plus 1 click))*
12.66	*12.33 (12 and 1 line (12 plus 1 click))*	*0.33 (0 and 1 line (Dial to 0 plus 1 click))*
11.00	*3.00 (3)*	*8.00 (8 (Dial to 8))*
12.00	*6.66 (6 and 2 lines (6 plus 2 clicks))*	*Round 5.34 to 5.33 (5 and 1 line (Dial to 5 and 1 click))*
18.33	*8.66 (8 and 2 lines (8 plus 2 clicks))*	*Round 9.67 to 9.66 (9 and 2 lines (Dial to 9 plus 2 clicks))*

Frequently Asked Questions (FAQ)

1. Is the priming step necessary before each injection?
   - No. Priming must be performed only before giving the first injection with a new pen.

2. How do I know that the injection is complete?
   - The injection button is firmly pushed in all the way until it stops.
   - The number ‘0’ is lined up with the dose indicator.
   - You have slowly counted to 5 while you are still holding the injection button in and the needle is still in your skin.

3. Why do I have to count to 5 while holding the injection button?
   - Holding the injection button for 5 seconds allows for the full dose to be injected and absorbed under your skin.

4. What if the dose knob cannot be turned to the required dose?
   - The cartridge in the pen may not have enough medicine left to deliver the prescribed dose.
   - The pen does not allow you to dial a larger dose than the dose that is left in the cartridge.
   - You can inject the medicine left in the pen and complete the prescribed dose with a new pen (split-dose) or use a new pen to give the full prescribed dose.

Warnings

- Do not use a pen if it has been dropped or hit against hard surfaces.
- If the injection button is not easy to push in, do not use force. Change the needle. If the injection button still is not easy to push in after changing the needle, use a new pen.
- Do not try to repair a damaged pen. If a pen is damaged, contact your healthcare provider or local representative of the marketing authorisation holder (please refer to package leaflet for contact details).

Additional information

<u>Needles</u>
Needles are supplied with your pen. If you need additional needles contact your healthcare provider. Use only needles that come with your REKOVELLE pre-filled pen or that your healthcare provider prescribes.

<u>Contact</u>
If you have any questions or problems related to the pen, contact your healthcare provider or local representative of the marketing authorisation holder (please refer to patient package for contact details).

1. Page numbers refer to the printed Instructions For Use booklet and not to actual page numbers in this document.

Package leaflet: Information for the user

REKOVELLE 72 micrograms/2.16 mL solution for injection in a pre-filled pen
follitropin delta

Read all of this leaflet carefully before you start using this medicine because it contains important information for you.

- Keep this leaflet. You may need to read it again.
- If you have any further questions, ask your doctor or pharmacist.
- This medicine has been prescribed for you only. Do not pass it on to others. It may harm them, even if their signs of illness are the same as yours.
- If you get any side effects, talk to your doctor or pharmacist. This includes any possible side effects not listed in this leaflet. See section 4.

What is in this leaflet

1. What REKOVELLE is and what it is used for
2. What you need to know before you use REKOVELLE
3. How to use REKOVELLE
4. Possible side effects
5. How to store REKOVELLE
6. Contents of the pack and other information

1. What REKOVELLE is and what it is used for

REKOVELLE contains follitropin delta, a follicle stimulating hormone which belongs to the family of hormones called gonadotropins. Gonadotropins are involved in reproduction and fertility.

REKOVELLE is used in the treatment of female infertility and in women undergoing assisted reproduction programmes such as *in vitro* fertilisation (IVF) and intracytoplasmic sperm injection (ICSI). REKOVELLE stimulates the ovaries to grow and develop many egg sacs (‘follicles’), from which eggs are collected and fertilised in the laboratory.

2. What you need to know before you use REKOVELLE

Before starting treatment with this medicine, a doctor should check you and your partner for possible causes of your fertility problems.

Do not use REKOVELLE

- if you are allergic to follicle stimulating hormone or any of the other ingredients of this medicine (listed in section 6)
- if you have a tumour of the uterus, ovaries, breasts, pituitary gland or hypothalamus
- if you have enlarged ovaries or cysts on your ovaries (unless caused by polycystic ovarian disease)
- if you suffer from bleeding from the vagina without any known cause
- if you have had an early menopause
- if you have malformations of the sexual organs which make a normal pregnancy impossible
- if you have fibroids of the uterus which make a normal pregnancy impossible.

Warnings and precautions
Talk to your doctor before using REKOVELLE.

Ovarian hyperstimulation syndrome
Gonadotropins like this medicine may cause ovarian hyperstimulation syndrome. This is when your follicles develop too much and become large cysts.
Talk to your doctor if you:
- have abdominal pain, discomfort or swelling
- have nausea
- are vomiting
- get diarrhoea
- gain weight
- have difficulty in breathing

Your doctor may ask you to stop using this medicine (see section 4).
If the recommended dose and schedule of administration are followed, ovarian hyperstimulation syndrome is less likely.

Blood clotting problems (thromboembolic events)
Clots in the blood vessels (veins or arteries) are more likely in women who are pregnant. Infertility treatment can increase the risk of this happening, especially if you are overweight or you or someone in your family (blood relative) have a known blood clotting disease (thrombophilia). Tell your doctor if you think this applies to you.

Twisting of ovaries
There have been reports of twisting of ovaries (ovarian torsion) following assisted reproductive technology treatment. Twisting of the ovary could cut off the blood flow to the ovary.

Multiple pregnancy and birth defects
When undergoing assisted reproductive technology treatment the possibility of having a multiple pregnancy (such as twins) is mainly related to the number of embryos placed inside your womb, the quality of the embryos, and your age. Multiple pregnancy may lead to medical complications for you and your babies. Furthermore, the risk of birth defects may be slightly higher following infertility treatment, which is thought to be due to characteristics of the parents (such as your age, and your partner's sperm characteristics) and multiple pregnancy.

Pregnancy loss
When undergoing assisted reproductive technology treatment, you are more likely to have a miscarriage than if you conceive naturally.

Pregnancy outside the uterus (ectopic pregnancy)
When undergoing assisted reproductive technology treatment, you are more likely to have a pregnancy outside the uterus (ectopic pregnancy) than if you conceive naturally. If you have a history of tubal disease, you have an increased risk of ectopic pregnancy.

Ovarian and other reproductive system tumours
There have been reports of ovarian and other reproductive system tumours in women who had undergone infertility treatment. It is not known if treatment with fertility medicines increase the risk of these tumours in infertile women.

Other medical conditions
Before starting to use this medicine, tell your doctor if:
- you have been told by another doctor that pregnancy would be dangerous for you
- you have kidney or liver disease

Children and adolescents (under 18 years of age)
This medicine is not indicated in children and adolescents.

Other medicines and REKOVELLE
Tell your doctor if you are using, have recently used or might use any other medicines.

Pregnancy and breast-feeding
Do not use this medicine if you are pregnant or breast-feeding.

Driving and using machines
This medicine does not affect your ability to drive and use machines.

REKOVELLE contains sodium
This medicine contains less than 1 mmol sodium (23 mg) per dose, that is to say essentially "sodium-free".

3. How to use REKOVELLE

Always use this medicine exactly as your doctor has told you and at the dose your doctor has told you. Check with your doctor if you are not sure.

The REKOVELLE dose for your first treatment cycle will be calculated by your doctor using the level of anti-Müllerian hormone (AMH, a marker of how your ovaries will respond to stimulation with gonadotropins) in your blood and your body weight. Therefore the AMH result from a blood sample (taken within the last 12 months) should be available before you start treatment. Your body weight will also be measured before you start treatment. The REKOVELLE dose is stated in micrograms.

The REKOVELLE dose is fixed for the whole treatment period with no adjustments to increase or decrease your daily dose. Your doctor will monitor the effect of REKOVELLE treatment, and treatment is stopped when an appropriate number of egg sacs are present. In general, you will be given a single injection of a medicine called human chorionic gonadotrophin (hCG) at a dose of 250 micrograms or 5,000 IU for final development of the follicles.

If your body´s response to treatment is too weak or too strong, your doctor may decide to stop treatment with REKOVELLE. For the next treatment cycle, your doctor will in this case give you either a higher or a lower daily dose of REKOVELLE than before.

How are injections given
The instructions for using the pre-filled pen must be followed carefully. Do not use the pre-filled pen if the solution contains particles or if the solution does not look clear.

The first injection of this medicine should be given under the supervision of a doctor or a nurse. Your doctor will decide if you can give yourself further doses of this medicine at home, but only after receiving adequate training.

This medicine is to be given by injection just under the skin (subcutaneously) usually in the abdomen. The pre-filled pen may be used for several injections.

If you use more REKOVELLE than you should
The effects of taking too much of this medicine are unknown. Ovarian hyperstimulation syndrome may possibly occur, which is described in section 4.

If you forget to use REKOVELLE
Do not take a double dose to make up for a forgotten dose. Please contact your doctor as soon as you notice that you forgot a dose.

If you have any further questions on the use of this medicine, ask your doctor.

4. Possible side effects

Like all medicines, this medicine can cause side effects, although not everybody gets them.

Serious side effects:
Hormones used in the treatment of infertility such as this medicine may cause a high level of activity in the ovaries (ovarian hyperstimulation syndrome). Symptoms may include pain, discomfort or swelling of the abdomen, nausea, vomiting, diarrhoea, weight gain or difficulty breathing. If you have any of these symptoms you should contact a doctor immediately.

The risk of having a side effect is described by the following categories:

Common (may affect up to 1 in 10 people):
- Headache
- Nausea
- Ovarian hyperstimulation syndrome (see above)
- Pelvic pain and discomfort, including of ovarian origin
- Tiredness (fatigue)

Uncommon (may affect up to 1 in 100 people):
- Mood swings
- Sleepiness/drowsiness
- Dizziness
- Diarrhoea
- Vomiting
- Constipation
- Discomfort of the abdomen
- Vaginal bleeding
- Breast discomfort (including breast pain, breast swelling, breast tenderness and/or nipple pain)

Reporting of side effects
If you get any side effects, talk to your doctor or pharmacist. This includes any possible side effects not listed in this leaflet. You can also report side effects directly via the national reporting system listed in Appendix V. By reporting side effects you can help provide more information on the safety of this medicine.

5. How to store REKOVELLE

Keep this medicine out of the sight and reach of children.

Do not use this medicine after the expiry date which is stated on the pre-filled pen label and carton after EXP. The expiry date refers to the last day of that month.

Store in refrigerator (2 °C - 8 °C). Do not freeze.
Store in the original package in order to protect from light.
REKOVELLE may be stored at or below 25 °C for up to 3 months including the period after first use. It must not be refrigerated again and must be discarded if it has not been used after 3 months.

After first use: 28 days when stored at or below 25 °C.

At the end of the treatment any unused solution must be discarded.

Do not throw away any medicines via wastewater or household waste. Ask your pharmacist how to throw away medicines you no longer use. These measures will help protect the environment.

6. Contents of the pack and other information

What REKOVELLE contains

- The active substance is follitropin delta.
  Each pre-filled pen with multidose cartridge contains 72 micrograms of follitropin delta in 2.16 millilitre of solution. One millilitre of solution contains 33.3 micrograms of follitropin delta in each millilitre of solution.
- The other ingredients are phenol, polysorbate 20, L-methionine, sodium sulphate decahydrate, disodium phosphate dodecahydrate, concentrated phosphoric acid, sodium hydroxide and water for injections.

What REKOVELLE looks like and contents of the pack

REKOVELLE is a clear and colourless solution for injection in a pre-filled pen (injection). It is available in packs of 1 pre-filled pen and 15 pen injection needles.

Marketing Authorisation Holder
Ferring Pharmaceuticals A/S
Amager Strandvej 405
2770 Kastrup
Denmark

Manufacturer
Ferring GmbH
Wittland 11
D-24109 Kiel
Germany

For any information about this medicine, please contact the local representative of the Marketing Authorisation Holder:

België/Belgique/Belgien
Ferring N.V.
Tel/Tél: +32 53 72 92 00
ferringnvsa@ferring.be

България
Фармонт ЕООД
Тел: +359 2 807 5022
farmont@farmont.bg

Česká republika
Ferring Pharmaceuticals CZ s.r.o.
Tel: +420 234 701 333
cz1-info@ferring.com

Danmark
Ferring Lægemidler A/S
Tlf: +45 88 16 88 17

Deutschland
Ferring Arzneimittel GmbH
Tel: +49 431 5852 0
info-service@ferring.de

Lietuva
CentralPharma Communications UAB
Tel: +370 5 243 0444
centralpharma@centralpharma.lt

Luxembourg/Luxemburg
Ferring N.V.
Belgique/Belgien
Tel/Tél: +32 53 72 92 00
ferringnvsa@ferring.be

Magyarország
Ferring Magyarország Gyógyszerkereskedelmi Kft.
Tel: +36 1 236 3800
ferring@ferring.hu

Malta
E.J. Busuttil Ltd.
Tel: +356 21447184
info@ejbusuttil.com

Nederland
Ferring B.V.
Tel: +31 235680300
infoNL@ferring.com

Eesti
CentralPharma Communications OÜ
Tel: +372 601 5540
centralpharma@centralpharma.ee

Ελλάδα
Ferring Ελλάς ΜΕΠΕ
Τηλ: +30 210 68 43 449

España
Ferring S.A.U.
Tel: +34 91 387 70 00
Registros@ferring.com

France
Ferring S.A.S.
Tél: +33 1 49 08 67 60
information.medicale@ferring.com

Hrvatska
Clinres farmacija d.o.o.
Tel: +385 1 2396 900
info@clinres-farmacija.hr

Ireland
Ferring Ireland Ltd.
Tel: +353 1 4637355
EnquiriesIrelandMailbox@ferring.com

Ísland
Vistor hf.
Sími: +354 535 70 00

Italia
Ferring S.p.A.
Tel: +39 02 640 00 11

Κύπρος
A.Potamitis Medicare Ltd
Τηλ: +357 22583333
a.potamitismedicare@cytanet.com.cy

Latvija
CentralPharma Communications SIA
Tālr: +371 674 50497
centralpharma@centralpharma.lv

Norge
Ferring Legemidler AS
Tlf: +47 22 02 08 80
mail@oslo.ferring.com

Österreich
Ferring Arzneimittel Ges.m.b.H
Tel: +43 1 60 8080
office@ferring.at

Polska
Ferring Pharmaceuticals Poland Sp. z o.o.
Tel: +48 22 246 06 80
PL0-Recepcja@ferring.com

Portugal
Ferring Portuguesa – Produtos Farmacêuticos, Sociedade Unipessoal, Lda.
Tel: +351 21 940 51 90

România
Ferring Pharmaceuticals Romania SRL
Tel: +40 356 113 270

Slovenija
SALUS, Veletrgovina, d.o.o.
Tel: +386 1 5899 100
regulatory@salus.si

Slovenská republika
Ferring Slovakia s.r.o.
Tel: +421 2 54 416 010
SK0-Recepcia@ferring.com

Suomi/Finland
Ferring Lääkkeet Oy
Puh/Tel: +358 207 401 440
info@ferring.fi

Sverige
Ferring Läkemedel AB
Tel: +46 40 691 69 00
info@ferring.se

United Kingdom (Northern Ireland)
Ferring Ireland Ltd.
Tel: +353 1 4637355
EnquiriesIrelandMailbox@ferring.com

This leaflet was last revised in .

Detailed information on this medicine is available on the European Medicines Agency web site: http://www.ema.europa.eu.

Instructions For Use

REKOVELLE pre-filled pen
follitropin delta

Your healthcare provider should show you how to prepare and inject REKOVELLE the right way before you inject it for the first time.
Do not try to inject yourself until you have been shown the right way to give your injections by your healthcare provider.
Read this booklet completely before using your REKOVELLE pre-filled pen and each time you get a new pen. There may be new information. Follow the instructions carefully even if you have used a similar injection pen before. Using the pen incorrectly could result in receiving an incorrect dose of medicine.
Call your healthcare provider (doctor, nurse or pharmacist) if you have any questions about how to give your REKOVELLE injection.

The REKOVELLE pre-filled pen is a disposable, dial-a-dose pen that can be used to give more than 1 dose of REKOVELLE. The pen is available in 3 different strengths:

- 12 micrograms/0.36 mL
- 36 micrograms/1.08 mL
- 72 micrograms/2.16 mL

REKOVELLE pre-filled pen and its parts

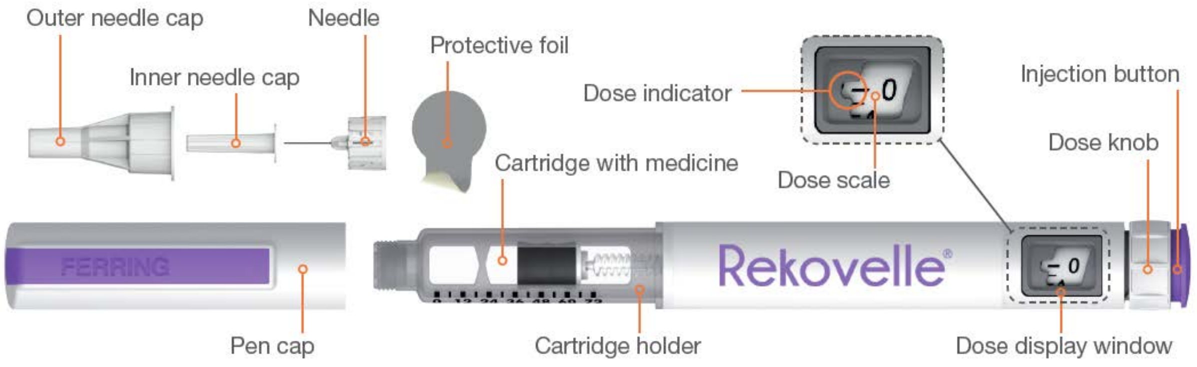

Instructions for use – REKOVELLE (follitropin delta) pre-filled pen

Important information

- The REKOVELLE pre-filled pen and the needles are for use by only one person and should not be shared with others.
- Use the pen only for the medical condition it is prescribed for and as directed by your healthcare provider.
- If you are blind or have poor eyesight and cannot read the dose scale on the pen, do not use this pen without help. Get help from a person with good eyesight who is trained to use the pen.
- Call your healthcare provider or local representative of the marketing authorisation holder (please refer to package leaflet for contact details) before giving your REKOVELLE injection if you have questions.

Information about your REKOVELLE pre-filled pen
The pen can be dialed to give doses from 0.33 micrograms to 20 micrograms of REKOVELLE in marked increments of 0.33 micrograms. See "Examples of how to dial a dose" at Page 20 to 21[1].

- The dose scale of the pen is numbered from 0 to 20 micrograms.
- Each number is separated by two lines, each line is equal to one increment of 0.33 micrograms.

- When turning the dial to your dose, you will hear a click sound and feel resistance on the dial for each increment to help you dial the correct dose.

Cleaning
- If needed, the outside of your pen may be cleaned with a cloth moistened with water.
- Do not put the pen in water or any other liquid.

Storage
- Always store the pen with the pen cap on and without a needle attached.
- Do not use the pen after the expiry date (EXP) printed on the pen label.
- Do not store the pen in extreme temperatures, direct sunlight or very cold conditions, such as in a car or freezer.
- Store the pen out of the reach of children and anyone who has not been trained to use the pen.

Before use:
- Store the pen in a refrigerator at 2 °C to 8 °C. Do not freeze.
- If stored outside the refrigerator (at or below 25 °C), the pen will last up to 3 months including the in-use period. Throw away (dispose of) the pen if it has not been used after 3 months.

After first use (in-use period):
- The pen may be stored for up to 28 days at or below 25°C. Do not freeze.

Supplies you will need to give your REKOVELLE injection

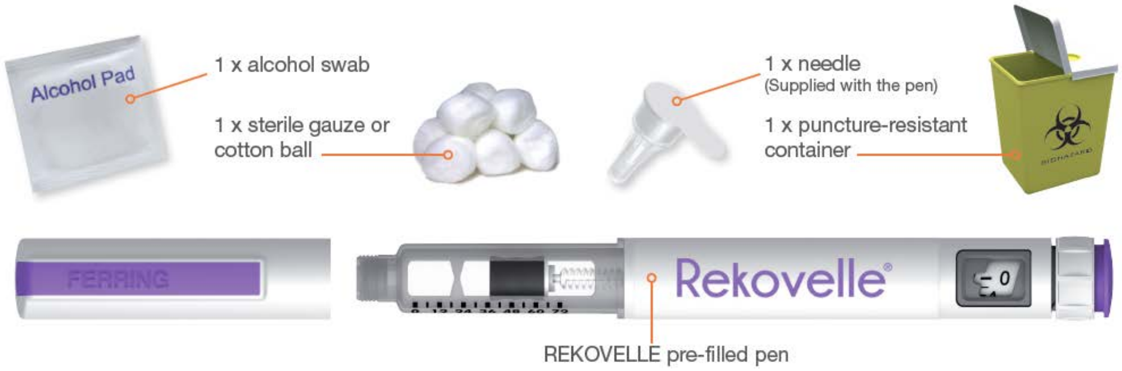

Before use – (Step 1)

<u>Step 1:</u>

- Wash your hands.
- Check the pen to see that it is not damaged. Do not use the pen if it is damaged.
- Check the pen (cartridge) to see that the medicine is clear and does not contain particles. Do not use a pen with particles or unclear medicine in the cartridge.
- Make sure you have the correct pen with correct strength.
- Check the expiration on the pen label.

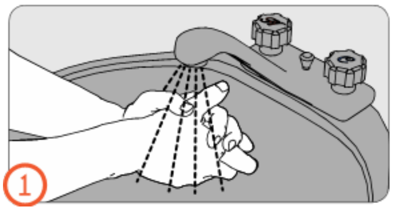

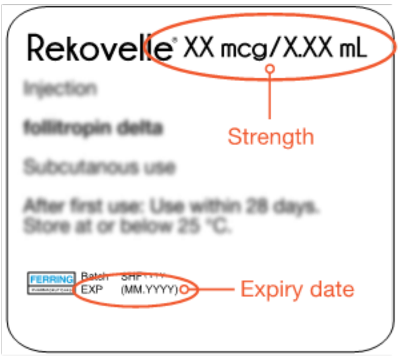

Attaching needle – (Step 2 to 6)

Important:

- Always use a new needle for each injection.
- Only use the single-use click-on needles supplied with the pen.

<u>Step 2:</u>

- Pull off the pen cap.

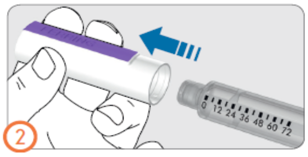

<u>Step 3:</u>

- Pull off the protective foil from the needle.

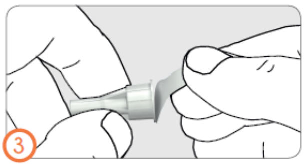

<u>Step 4:</u>

- Click on the needle.
- You will hear or feel a click when the needle is safely on.
- You may also screw on the needle. When you feel a light resistance it is safely on.

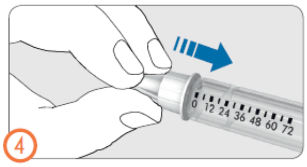

Step 5:

- Pull off the outer needle cap.
- Do not throw the outer needle cap away. You will need it to throw away (dispose of) the needle after injecting the medicine.

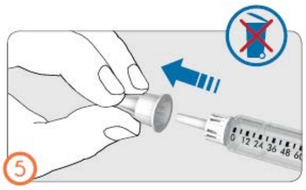

Step 6:

- Pull off the inner needle cap and throw it away.

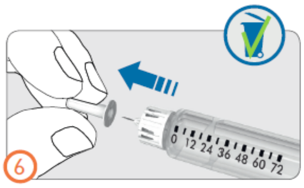

Priming – (Step 7 to 9)

- Before using the pen for the first time, you need to remove air bubbles from the cartridge (Priming) to receive the correct dose of medicine.
- Only prime your pen the first time you use it.
- Perform Step 7 to 9 even if you do not see air bubbles.
- If the pen has already been used proceed directly to Step 10.

Step 7:

- Turn the dose knob clockwise until a symbol of a droplet lines up with the dose indicator.
- If you dial the incorrect priming dose, the priming dose can be corrected either up or down without loss of medicine by turning the dose knob in either direction until the symbol of a droplet lines up with the dose indicator.

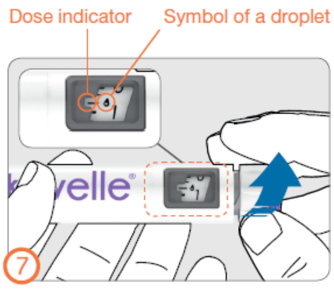

Step 8:

- Hold the pen with the needle pointing upwards.
- Tap with your finger on the cartridge holder to make any air bubbles in the cartridge rise to the top of the cartridge.

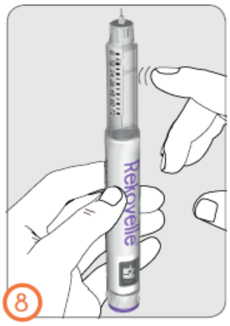

Step 9:

- With the needle still pointing upwards (away from the face) press in the injection button all the way in until you see the number ‘0’ lined up with the dose indicator.
- Check that a droplet of liquid appears at the tip of the needle.
- If no droplet(s) appear repeat Steps 7 to 9 (Priming) until a droplet appears.
- If no droplet appears after 5 tries, remove the needle (See Step 13), attach a new needle (See Steps 3 to 6), and repeat priming (See Steps 7 to 9).
- If you still do not see a droplet after using a new needle, try a new pen.

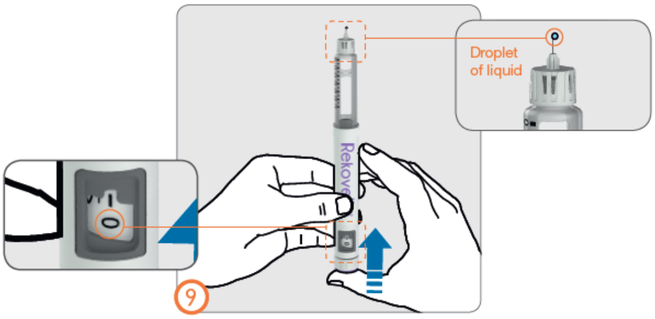

Dialing the dose – (Step 10)

See “Examples of how to dial a dose” at Page 20 to 21[1].

Step 10:

- Turn the dose knob clockwise until the prescribed dose lines up with the dose indicator in the dose display window.
- The dose can be corrected either up or down without loss of medicine by turning the dose knob in either direction until the correct dose lines up with the dose indicator.
- Do not press the injection button when dialing the dose to avoid loss of medicine.

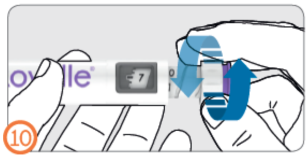

Split-dosing:

- You may need more than one pen to complete your prescribed dose.
- If you are not able to dial your complete dose, this means there is not enough medicine left in the pen. You will need to give a split-dose injection or throw away (dispose of) your current pen and use a new pen for your injection.

See “Giving a split-dose of REKOVELLE“ at Page 22 to 23[1] for examples of how to calculate and record your split dose.

Injecting the dose – (Step 11 to 12)

Important:

- Do not use the pen if the medicine contains particles or if the medicine is not clear.
- Read Step 11 and 12 at Page 14 to 15[1] before giving your injection.
- This medicine should be given by injection just under the skin (subcutaneously) in the stomach-area (abdomen).
- Use a new injection site for each injection to lower the risk of skin reactions as redness and irritation.
- Do not inject into an area that is sore (tender), bruised, red, hard, scarred or where you have stretch marks.

Step 11 and 12:

- Wipe the skin of your injection site with an alcohol swab to clean it. Do not touch this area again before you give your injection.
- Hold the pen so the dose display window is visible during injection.
- Pinch your skin and insert the needle straight into your skin as shown by your healthcare provider. Do not touch the injection button yet.
- After the needle is inserted, place your thumb on the injection button.

- Press the injection button all the way in and hold.
- Keep pressing the injection button in and when you see the number ‘0’ lined up with the dose indicator, wait for 5 seconds (slowly count to 5). This will make sure you get your full dose.

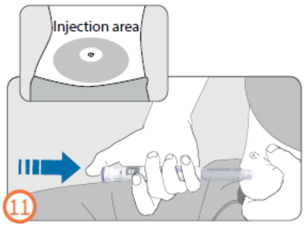

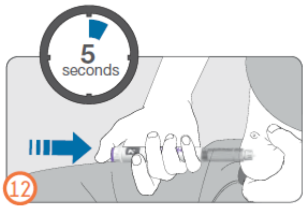

- After pressing in the injection button for 5 seconds, release the injection button. Then slowly remove the needle from the injection site by pulling it straight out of the skin.
- If blood appears at the injection site, press a gauze pad or cotton ball lightly to the injection site.

Note:
- Do not tilt the pen during injection and removal from skin.
- Tilting the pen can cause the needle to bend or break off.
- If a broken needle remains stuck in the body or remains under the skin, get medical help right away.

Disposal of Needle – (Step 13)

<u>Step 13:</u>
- Carefully replace the outer needle cap over the needle with a firm push (A).
- Unscrew the needle in counter-clockwise direction to remove the needle from the pen (B+C).
- Throw away (dispose of) the used needle carefully (D).
- See “Disposal” at Page 18[1].

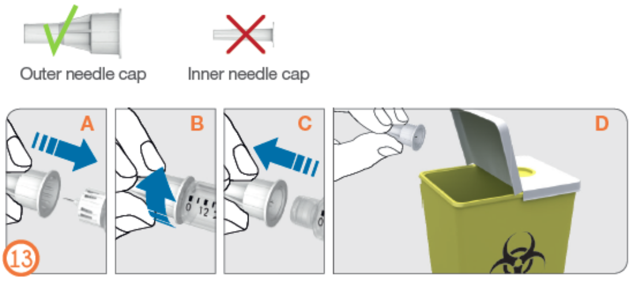

Note:
- Always remove the needle after every use. The needles are for single-use only.
- Do not store the pen with the needle attached.

Replace pen cap – (Step 14)

<u>Step 14:</u>
- Firmly replace the pen cap on the pen for protection between injections.

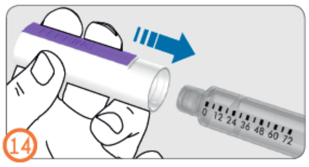

Note:

- The pen cap will not fit over a needle.
- If you will give a split-dose injection, only throw away (dispose of) the pen when it is empty.
- If you will use a new pen to give your full prescribed dose instead of giving a split-dose injection, throw away (dispose of) your pen when there is not enough medicine in the pen for a full dose.
- Keep the pen cap on the pen when it is not in use.

Disposal

Needles:
Put your used needles in a puncture-resistant container, such as a sharps disposal container right away after use. Do not throw away (dispose of) your used sharps disposal container in your household trash.

If you do not have a sharps disposal container, you may use a household container that is:
- made of a heavy-duty plastic,
- can be closed with a tight-fitting, puncture-resistant lid, without sharps being able to come out,
- upright and stable during use,
- leak-resistant, and
- properly labeled to warn of hazardous waste inside the container.

REKOVELLE pre-filled pens:
- Throw away (dispose of) your used pens in accordance with local waste regulations.

Examples of how to dial a dose

<u>Examples of how to dial a dose using your REKOVELLE pre-filled pen</u>
The chart below shows examples of prescribed doses, how to dial the examples of prescribed doses, and what the dose display window looks like for the prescribed doses.

**Examples of prescribed dose (in micrograms)**	**Dose to dial on pen**	**Dose display window for example of prescribed dose**
0.33	0 and 1 line<br>(Dial to 0 plus 1 click)	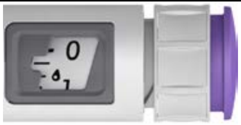
0.66 (priming dose)	0 and 2 lines<br>(Dial to 0 plus 2 clicks)	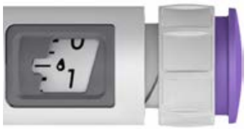
2.33	2 and 1 line<br>(Dial to 2 plus 1 click)	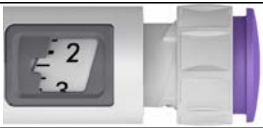
11.00	11<br>(Dial to 11)	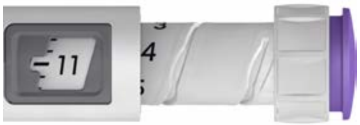
12.33	12 and 1 line<br>(Dial to 12 plus 1 click)	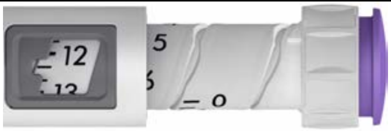
18.66	18 and 2 lines<br>(Dial to 18 plus 2 clicks)	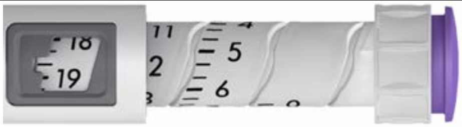
20.00	20 (Dial to 20)	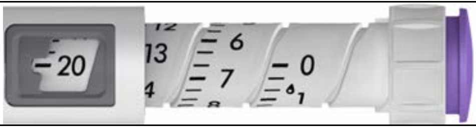

Giving a split-dose of REKOVELLE

If you are not able to dial the full prescribed dose in your pen, this means that there is not enough medicine left in the pen to give the full dose. You will need to give part of your prescribed dose using

your current pen and the remainder of the dose using a new pen (split-dose injection) or you may throw away (dispose of) the pen you are using and use a new pen to give your full prescribed dose in 1 injection. If you decide to give a split-dose injection, follow these instructions and write down how much medicine to give using the split-dose diary on Page 23[1].

- Column A shows an example of a prescribed dose. Write down your prescribed dose in column A.
- Column B shows an example of the dose that is left in the pen (this is equal to what you are able to dial).
- Write down the dose that is left in your pen in column B. Give the injection using the rest of the medicine that is left in your pen.
- Prepare and prime a new pen (Step 1 to 9).
- Calculate and write down the remaining dose to inject in column C by subtracting the number in column B from the number in column A. Use a calculator to check your math if needed.
- See “Examples of how to dial a dose” at Page 20 to 21[1] if needed.
- Doses should be rounded off to nearest increment, X.00, X.33 or X.66 micrograms. For example, if the number in column C is 5.34, round your remaining dose to 5.33. If the number in column C is 9.67, round your remaining dose to 9.66.
- Call your healthcare provider if you have questions about how to calculate your split-dose.
- Inject the remaining dose of medicine (the number in column C) using your new pen to complete your prescribed dose.

Split-dose diary

A<br>Prescribed Dose	B<br>Dose left in pen<br>(Dose shown at dose indicator in dose display window)	C = A minus B<br>Dose to inject on new pen<br>(Dose shown at dose indicator in dose display window)
11.33	*4.00 (4)*	*7.33 (7 and 1 line (Dial to 7 plus 1 click))*
12.66	*12.33 (12 and 1 line (12 plus 1 click))*	*0.33 (0 and 1 line (Dial to 0 plus 1 click))*
11.00	*3.00 (3)*	*8.00 (8 (Dial to 8))*
12.00	*6.66 (6 and 2 lines (6 plus 2 clicks))*	*Round 5.34 to 5.33 (5 and 1 line (Dial to 5 and 1 click))*
18.33	*8.66 (8 and 2 lines (8 plus 2 clicks))*	*Round 9.67 to 9.66 (9 and 2 lines (Dial to 9 plus 2 clicks))*

Frequently Asked Questions (FAQ)

1. Is the priming step necessary before each injection?
   - No. Priming must be performed only before giving the first injection with a new pen.

2. How do I know that the injection is complete?
   - The injection button is firmly pushed in all the way until it stops.
   - The number ‘0’ is lined up with the dose indicator.
   - You have slowly counted to 5 while you are still holding the injection button in and the needle is still in your skin.

3. Why do I have to count to 5 while holding the injection button?
   - Holding the injection button for 5 seconds allows for the full dose to be injected and absorbed under your skin.

4. What if the dose knob cannot be turned to the required dose?
   - The cartridge in the pen may not have enough medicine left to deliver the prescribed dose.
   - The pen does not allow you to dial a larger dose than the dose that is left in the cartridge.
   - You can inject the medicine left in the pen and complete the prescribed dose with a new pen (split-dose) or use a new pen to give the full prescribed dose.

Warnings

- Do not use a pen if it has been dropped or hit against hard surfaces.
- If the injection button is not easy to push in, do not use force. Change the needle. If the injection button still is not easy to push in after changing the needle, use a new pen.
- Do not try to repair a damaged pen. If a pen is damaged, contact your healthcare provider or local representative of the marketing authorisation holder (please refer to package leaflet for contact details).

Additional information

Needles
Needles are supplied with your pen. If you need additional needles contact your healthcare provider. Use only needles that come with your REKOVELLE pre-filled pen or that your healthcare provider prescribes.

Contact
If you have any questions or problems related to the pen, contact your healthcare provider or local representative of the marketing authorisation holder (please refer to package leaflet for contact details).

---

1. Page numbers refer to the printed Instructions For Use booklet and not to actual page numbers in this document.
