# Supplementary material for: Effectiveness and safety of follitropin delta in routine clinical practice in the Nordics and Switzerland (the NORSOS study): a prospective non-interventional study
Source: Front Endocrinol (Lausanne). 2025 Sep 5;16:1613680. doi: 10.3389/fendo.2025.1613680 (PMC12446022; doi:10.3389/fendo.2025.1613680)
Supplement: Supplementary file 3 [file Table1.docx]

Supplementary Tables

# Supplementary Table 1. Patient questionnaire

Subject questionnaire to assess overall experience and convenience among subjects using follitropin delta (REKOVELLE^®^ pre-filled pen for injection during COS therapy for ART treatment).

REKOVELLE^®^ is subject to safety monitoring. This will allow quick identification of new safety information. You can help by reporting any side effects you may get.

**1.** How many days did you use the REKOVELLE^®^ pen?

☐ 9 days

☐ 10 days

☐ 11 days

☐ 12 days

☐ Other: (enter number of days)

**2.** Who explained to you the handling of the REKOVELLE^®^ pre-filled ready-to-use pen? (Tick more than one if applicable)

☐ Physician

☐ Nurse

☐ Self-training (Instruction for use leaflet)

☐ If other (text box) (e.g., company website, social media platforms etc.)

**3.** Who administered injections? (Tick more than one if applicable)

☐ Myself

☐ Partner

☐ Both

☐ Other: (text box)


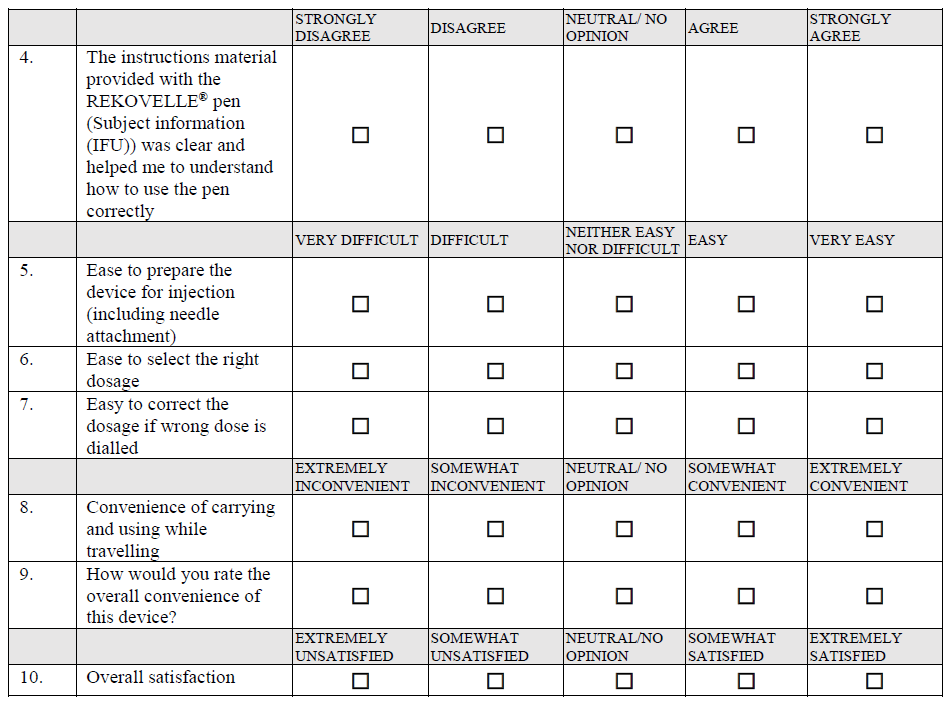
ART, assisted reproductive technology; COS, controlled ovarian stimulation; IFU, instructions for use.

# Supplementary Table 2. Oocyte retrieval per AMH subgroup

|  | **Overall** | **By AMH level** | | | |
| --- | --- | --- | --- | --- | --- |
|  |  | **<7 pmol/L** | **≥7 and <15 pmol/L** | **≥15 and ≤35 pmol/L** | **>35 pmol/L** |
| **Number of participants, N** | 199 | 15 | 54 | 104 | 26 |
| **Mean total of oocytes retrieved** | 12.1 ± 6.9 | 7.0 ± 5.1 | 9.6 ± 4.8 | 13.4 ± 6.3 | 15.1 ± 10.3 |
| **Total number of oocytes retrieved**^a^ | 194 | 14 | 53 | 103 | 24 |
| Poor response (<4 oocytes) | 10 (5.2) | 2 (14.3) | 5 (9.4) | 2 (1.9) | 1 (4.2) |
| Suboptimal response (4–7 oocytes) | 36 (18.6) | 8 (57.1) | 13 (24.5) | 11 (10.7) | 4 (16.7) |
| Normal response (8–14 oocytes) | 93 (47.9) | 3 (21.4) | 27 (50.9) | 52 (50.5) | 11 (45.8) |
| Potential high response (15–19 oocytes) | 33 (17.0) | 1 (7.1) | 5 (9.4) | 25 (24.3) | 2 (8.3) |
| High response (≥20 oocytes) | 22 (11.3) | 0 (0) | 3 (5.7) | 13 (12.6) | 6 (25.0) |
| **Total number of frozen embryos** | 3.2 ± 3.8 | 1.1 ± 1.9 | 2.6 ± 3.0 | 3.6 ± 3.7 | 4.1 ± 5.6 |

^a^Percentages calculated among participants with no cycle cancellation before oocyte pickup.

AMH, anti-Müllerian hormone.

# Supplementary Table 3. Cycle and transfer cancellation per age category

|  | **Overall** | **By age group (years)** | | | |
| --- | --- | --- | --- | --- | --- |
|  |  | **<35** | **≥35 and ≤37** | **>37 and ≤40** | **>40** |
| **Number of participants, N** | 199 | 147 | 31 | 18 | 3 |
| **Women with cycle cancellation (before oocyte pickup)^a^** | 5 (2.5) | 3 (2.0) | 2 (6.5) | 0 | 0 |
| **Reason for cycle cancellation^b,c^** |  |  |  |  |  |
| Poor ovarian response | 2 (40.0) | 2 (66.7) | 0 | 0 | 0 |
| Excessive ovarian response | 0 | 0 | 0 | 0 | 0 |
| Participant not taking triggering method at the correct time | 1 (20.0) | 0 | 1 (50.0) | 0 | 0 |
| Participant choice | 2 (40.0) | 1 (33.3) | 1 (50.0) | 0 | 0 |
| Any illness that prevents oocyte collection procedure | 0 | 0 | 0 | 0 | 0 |
| Other | 0 | 0 | 0 | 0 | 0 |
| **Women with transfer cancellation (after oocyte pickup)^a^** | 88 (44.2) | 70 (47.6) | 10 (32.3) | 7 (38.9) | 1 (33.3) |
| **Reason for transfer cancellation^d^** |  |  |  |  |  |
| No oocytes fertilized | 3 (3.4) | 3 (4.3) | 0 | 0 | 0 |
| Abnormal fertilization | 1 (1.1) | 1 (1.4) | 0 | 0 | 0 |
| Abnormal embryo development | 6 (6.8) | 3 (4.3) | 2 (20.0) | 1 (14.3) | 0 |
| No embryo development | 10 (11.4) | 7 (10.0) | 2 (20.0) | 0 | 1 (100.0) |
| OHSS | 6 (6.8) | 6 (8.6) | 0 | 0 | 0 |
| Other^e^ | 62 (70.5) | 50 (71.4) | 6 (60.0) | 6 (85.7) | 0 |
| Missing | 5 | 3 | 2 | 0 | 0 |

Values represented as n (%), unless stated otherwise.

^a^Percentages calculated from all participants (N = 199).

^b^Percentages calculated from participants with cycle cancellation before oocyte pickup.

^c^Displayed reasons for cancellation are for fresh transfer cancellation only, as eCRF did not allow the collection of frozen transfer cancellation and their reasons.

^d^Percentages calculated from participants with transfer cancellation after oocyte pickup.

^e^This category includes additional reasons specified by the investigating clinician, such as implementation of a freeze-all strategy as a preventive measure for OHSS, failure of the blastocyst to reach day 5, or pending results from embryo genetic testing.

eCRF, electronic case-report form; N, number of participants; n, number of participants per category; OHSS, ovarian hyperstimulation syndrome.

# Supplementary Table 4. Luteal-phase support by age group

|  | **Overall** | **By age group (years)** | | | |
| --- | --- | --- | --- | --- | --- |
|  |  | **<35** | **≥35 and ≤37** | **>37 and ≤40** | **>40** |
| **Number of participants, N** | 194 | 144 | 29 | 18 | 3 |
| **Luteal-phase support (Yes)^a^** | 125 (64.4) | 87 (60.4) | 23 (79.3) | 12 (66.7) | 3 (100.0) |
| Progesterone | 124 (99.2) | 86 (98.9) | 23 (100.0) | 12 (100.0) | 3 (100.0) |
| Estrogen | 0 | 0 | 0 | 0 | 0 |
| hCG | 6 (4.8) | 5 (5.7) | 0 | 1 (8.3) | 0 |
| Other | 7 (5.6) | 7 (8.0) | 0 | 0 | 0 |

Values represented as n (%), unless stated otherwise.

^a^Percentages calculated based on the total number of women with at least one luteal-phase support drug used. More than one support drug can be selected, so percentages may not add up to 100%.

hCG, human chorionic gonadotropin; N, number of participants; n, number of participants per category.
